# Trade‐Offs Between Carbon and Water Fluxes Along a Land Use Intensity Gradient in Southeast Asian Forests and Plantations

**DOI:** 10.1111/gcb.70753

**Published:** 2026-02-24

**Authors:** Bayu Budi Hanggara, Christian Stiegler, Yoshiaki Hata, Lulie Melling, Tania June, Tomo'omi Kumagai, Takashi Hirano, Alexander Knohl

**Affiliations:** ^1^ University of Göttingen, Bioclimatology Göttingen Germany; ^2^ Department Biogeochemical Integration, Max Planck Institute for Biogeochemistry Jena Germany; ^3^ Earth Sciences New Zealand Wellington New Zealand; ^4^ Graduate School of Agricultural and Life Sciences The University of Tokyo Bunkyo‐ku Tokyo Japan; ^5^ Sarawak Tropical Peat Research Institute Kota Samarahan Malaysia; ^6^ Department of Geophysics and Meteorology IPB University Bogor Indonesia; ^7^ Water Resources Research Center University of Hawaiʻi at Mānoa Honolulu Hawaii USA; ^8^ Research Faculty of Agriculture Hokkaido University Sapporo Japan

**Keywords:** carbon fluxes, eddy covariance, forests, land use intensity, plantations

## Abstract

Land use intensity (LUI) significantly influences the biophysical and biogeochemical properties of the global landscape. The impact of LUI is exceptionally strong in Southeast Asia (SEA), where forests are increasingly being replaced by intensively managed plantations. Despite these transformations, comprehensive studies on how different LUI regulate carbon, energy, and water fluxes in this region remain scarce. In this study, we examine data from 16 eddy covariance (EC) flux tower sites in SEA, representing a total of 112 years of measurements. We aim to assess trade‐offs in carbon fluxes, light use efficiency (LUE), and water use efficiency (WUE) across a gradient of LUI: low (primary forests), medium (secondary forests), and high (plantations). Our findings reveal that mature high LUI sites on mineral soil act as carbon sinks; however, their high evapotranspiration rates often exceed site‐specific precipitation, making them susceptible to water stress. For example, mean annual carbon uptake at high LUI sites (ranged from −1.19 to −0.74 kg C m^−2^ year^−1^) outperformed low LUI sites (−0.85 to −0.02 kg C m^−2^ year^−1^). The strong carbon uptake on high LUI had an exception when the ecosystem was in the establishment phase and managed on peat soil (0.98 kg C m^−2^ year^−1^). However, WUE was greater at low LUI (mean annual: 2.63 to 6.50 g C kg^−1^ H_2_O^−1^) compared to high LUI sites (2.08 to 3.53 g C kg^−1^ H_2_O^−1^), illustrating a trade‐off between carbon uptake and water use. Additionally, while high LUI sites required less radiation to achieve maximum gross primary productivity, their mean daily LUE was not higher compared to low LUI sites. These findings underscore the importance of carefully balancing carbon sequestration goals with water resource considerations and drought resilience when promoting plantation systems. Conversely, forest conservation offers advantages for water security and ecosystem resilience to face climate change.

## Graphical abstract

Increase land use intensity may enhance net carbon uptake in fast‐growing species on mineral soil but concurrently increases the risk of water scarcity. Net carbon uptake and evapotranspiration in high land use intensity sites are more affected by water availability compared to low land use intensity sites.

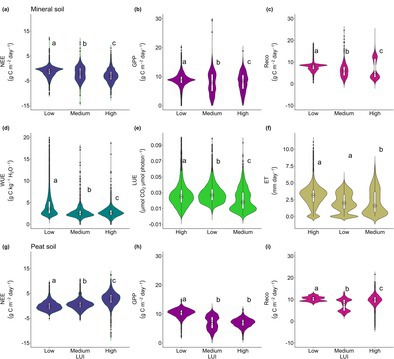

## 1 Introduction

Southeast Asia (SEA) plays an important role in global biogeochemical cycles, and it is home to approximately 15% of the world's tropical forests (Ometto 2022). However, this region has experienced rapid land use and land cover change (LULCC) in recent decades (Vadrevu and Ohara 2020), leading to extensive deforestation and landscape change (Austin et al. 2019). SEA has emerged as a major hotspot of deforestation (Jamaludin et al. 2022), with annual forest losses of about 1.75 Mha (0.6% year^−1^ of total forest area) in the 1990s and 1.45 Mha (0.59% year^−1^) in the 2000s (Stibig et al. 2014). In parallel, the expansion of fast‐growing tree plantations, i.e., oil palm, rubber, and pulpwood, has skyrocketed, increasing to 10.6 Mha in the 1990s and 7.1 Mha in the 2000s, respectively (Stibig et al. 2014; Xu et al. 2020). The impact of LULCC in SEA is extensive, affecting land surface properties, vegetation structure, and thus biogeochemical cycles (Sabajo et al. 2017). On the other hand, primary forests in SEA provide essential ecosystem services, including carbon sequestration and water regulation, which are critical for ecosystem resilience (Carnol et al. 2014). However, intensive land management practices have altered these landscapes, changing carbon and water cycles and decreasing their ability to sustain these services (Calle et al. 2016; Guillaume et al. 2018).

To better understand the impacts of LULCC on ecosystem functions, the eddy covariance (EC) method has been widely used to quantify carbon, water, and energy fluxes in the Asia region (see http://asiaflux.net/). Previous studies indicate that the dynamics of carbon fluxes in Asia vary at different temporal and spatial scales (Hirano et al. 2024; Ichii et al. 2017; Kondo et al. 2017; Takamura et al. 2023; Yu et al. 2014) and are influenced by various factors such as: vegetation age, biomass productivity, and ecological conditions (Ma et al. 2020). Those factors regulate the carbon sequestration capacity of ecosystems (Kondo et al. 2017). On the other hand, water fluxes, which are critical for plant growth and carbon uptake (Peddinti et al. 2019), are strongly affected by land use change, with monocultures often proving more vulnerable to hydroclimatic and ecological disturbances (Levia et al. 2020).

The coupling of water and energy fluxes with carbon uptake can be expressed through functional properties of ecosystems derived from EC measurements, such as water‐use efficiency (WUE) and light‐use efficiency (LUE) (Musavi et al. 2015). Land use intensity (LUI), which can vary from small to large‐scale changes, such as extensive agriculture practices, can lead to trade‐offs between carbon, energy, and water fluxes (Beckmann et al. 2019). Effective silvicultural practices often play an important role in maximize WUE and ecosystem productivity (Aguirre and Trilleras 2024). In tropical forests, land cover changes generally had a negative impact on WUE (Zeng et al. 2025). Meanwhile, old‐growing forests usually showed less carbon assimilation due to a slow turnover rate, as a consequence of the domination of old trees (Kunert and Aparecido 2024). These contrasting relationships across different LUI underscore the complexity of ecosystem response and highlight the importance of investigating the interactions between carbon and water fluxes concerning LUI.

Although research on land‐atmosphere interactions in Asia regions has grown, the majority of studies still focus on carbon fluxes within primary forests (Kondo et al. 2017; Takamura et al. 2023). Research into carbon fluxes within intensive managed systems, such as oil palm or rubber plantations is emerging, though it remains concentrated in specific areas, with notable studies conducted in Indonesia (Deshmukh et al. 2023; Meijide et al. 2018; Stiegler et al. 2019), Thailand (Kumagai et al. 2015; Wang et al. 2022), and Malaysia (Kiew et al. 2020). Although previous studies have explored how the impact of logging intensity affects carbon stock and fluxes in Sabah and Sarawak, Malaysia (Mills et al. 2023; Riutta et al. 2021, 2018), there is still a knowledge gap on the effects of high LUI practices in cash crop plantations, where regular fertilization, weeding, and water management are implemented. To date, research on particularly the trade‐offs between carbon and water fluxes across different land ecosystems in SEA, remains underexplored.

Therefore, this study aims to improve our understanding of land‐atmosphere exchange processes under different LUI in SEA, focusing on carbon and water fluxes. Our objectives are threefold: first, to assess the impact of LUI on ecosystem carbon fluxes; second, to investigate the pattern of LUI gradient on canopy light response curve; and third, to evaluate the impact of LUI on WUE. This research is designed to understand the trade‐offs of LUI gradients in WUE and carbon uptake, providing insights into potential risks and anticipating future land use intensification in SEA.

## 2 Materials and Methods

### 2.1 Study Sites

**FIGURE 1:**
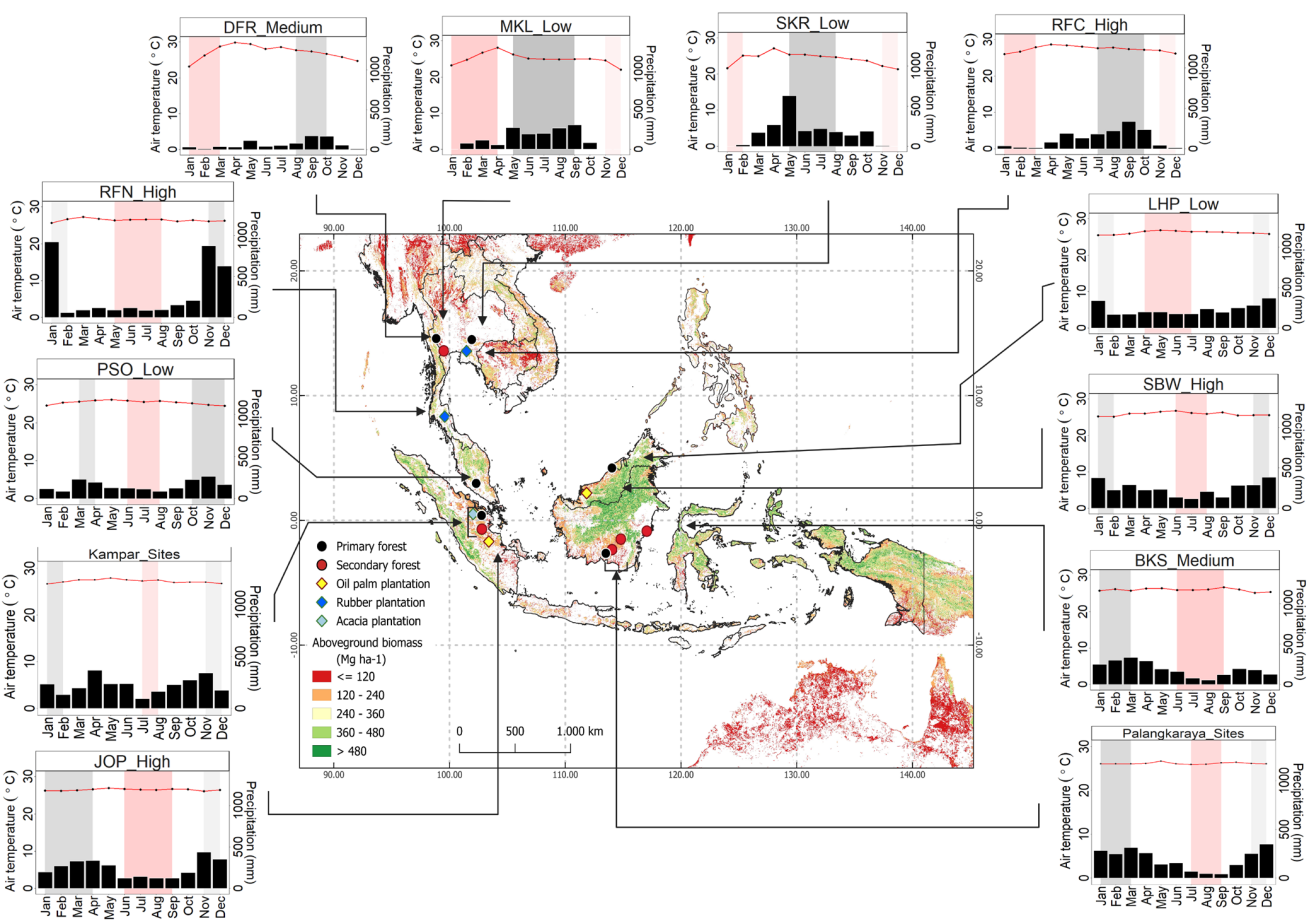
The distribution of 16 EC sites (site code refers to Table 1) consists of low LUI (black dots: Primary forests), medium LUI (red dots: Secondary forests), and high LUI (yellow dots: Oil palm plantations, dark blue dots: Rubber plantations, light blue dot: Acacia plantation). The background map shows the aboveground biomass illustrated according to Avitabile et al. (2016). Additional figures represent the monthly average of air temperature (red line) and precipitation (bar plot) for each site. The grey background represents the wet season, while the light red background refers to the dry season based on precipitation patterns. Map lines delineate study areas and do not necessarily depict accepted national boundaries.

Ecosystem carbon, water fluxes, and meteorological parameters of 16 eddy covariance (EC) sites across SEA were compiled from the Asiaflux research network (http://asiaflux.net/) and relevant literature (Table 1). All datasets used in this study (e.g., half‐hourly or daily carbon fluxes) were publicly available and had been published for respective study sites. Each study site is located in tropical regions characterized by a tropical humid climate (Af) and tropical monsoon climate (Am) according to the Köppen climate classification (Kottek et al. 2006). The location and precipitation pattern of each study site are depicted in Figure 1, with the mean annual climatic characteristics for every site listed in Table S7.

**TABLE 1:** Site description, location, elevation, climatic condition, ecosystem type, and data availability.

Land use intensity	Site name (Code)	Code	Country	Location	Ecosystem type	Mean annual temperature (°C)	Annual precipitation (mm)	Soil type	Data availability	References
Low (1)	Lambir Hills National Park	LHP	Malaysia	4° 12′ 3.6″ N, 114° 02′ 20.7″ E	Mixed dipterocarp forest	26.2 ± 1.0	2751.1 ± 449.6	Mineral soil	2010–2019	Asiaflux (Kumagai et al. 2005, 2009; Takamura et al. 2023)
	Mae Klong	MKL	Thailand	14° 34′ 34.6″ N, 98° 50′ 38.0″ E	Seasonal deciduous forest	25.1 ± 1.9	1223.0 ± 685.9	Mineral soil	2003–2004	Asiaflux (Saigusa et al. 2013)
	Pasoh Forest Reserve	PSO	Malaysia	2° 58′ N, 102° 18′ E	Mixed dipterocarp forest	25.3 ± 1.0	1864.8 ± 288.0	Mineral soil	2003–2009	Asiaflux (Kosugi et al. 2012)
	Sakaerat	SKR	Thailand	14° 29′ 32.5″ N, 101° 54′ 58.7″ E	Tropical seasonal evergreen forest	24.2 ± 2.3	2004.9 ± 963.2	Mineral soil	2001–2003	Asiaflux (Saigusa et al. 2013)
	Kampar Intact Forest	KIF	Indonesia	0° 23′ 42.735″ N, 102° 45′ 52.38″ E	Tropical intact peatland	26.9 ± 1.0	1530 ± 437	Peat soil	2017–2022	Deshmukh et al. (2021, 2023)
	Palangkaraya undrained forest	PUF	Indonesia	2.32° S, 113.9° E	Undrained tropical peatland	26.10 ± 0.43	2688.71 ± 462.69	Peat soil	2005–2017	Hirano et al. (2024)
Medium (2)	Bukit Soeharto	BKS	Indonesia	0°51′41″ S, 117°02′41″ E	Tropical secondary forest	26.0 ± 3.2	2275.5 ± 368.4	Mineral soil	2001–2002	Asiaflux (Kato and Tang 2008)
	Kampar degraded forest	KDF	Indonesia	0° 41′ 58.17″ N, 102° 47′ 35.89″ E	Tropical degraded peatland	27.5 ± 1.2	1942 ± 347	Peat soil	2016–2020	Deshmukh et al. (2021, 2023)
	Dry dipterocarp forest Ratchaburi	DFR	Thailand	13° 35′ 13.3″ N, 99° 30′ 3.9″ E	Dry dipterocarp secondary forest	27.3 ± 2.2	1055 ± 192	Mineral soil	2015–2017	Asiaflux Wang et al. (2022)
	Palangkaraya drained forest	PDF	Indonesia	2° 20′ 42″ S, 11 4°2′11″ E	Drained peat secondary forest	26.2 ± 2.9	2163.9 ± 487.8	Peat soil	2002–2016	Asiaflux (Hirano et al. 2024)
	Palangkaraya drained burned forest	PDB	Indonesia	2.34° S, 114.04° E	Drained burned peat forest	26.36 ± 1.17	2649.93 ± 473.66	Peat soil	2005–2016	Hirano et al. (2024)
High (3)	Jambi oil palm plantation	JOP	Indonesia	1° 41′ 35.0″ S, 103° 23′ 29.0″ E	Oil palm plantation	26.5 ± 0.9	2569.5 ± 1118.9	Mineral soil	2014–2020	Asiaflux (Meijide et al. 2017)
	Sibu Station	SBW	Malaysia	2° 11′ 12″ N, 111° 50′ 35.7″ E	Oil palm plantation	25.8 ± 0.6	2898 ± 68	Peat soil	2011–2014	Asiaflux (Kiew et al. 2020)
	Kampar Acacia plantation	KAP	Indonesia	0° 30′ 57.221″ N, 102° 2′ 11.090″ E	Acacia plantation	27.0 ± 1.0	2228 ± 437	Peat soil	2016–2020	Deshmukh et al. (2021, 2023)
	Rubber flux Chachoengsao	RFC	Thailand	13° 34′ N, 101° 28′ E	Rubber plantation	27.4 ± 1.6	1510.2 ± 852.7	Mineral soil	2015–2018	Wang et al. (2022)
	Rubber flux Nakhon si Thammarat	RFN	Thailand	8° 19′ N, 99° 35′ E	Rubber plantation	26.4 ± 1.1	3744.0 ± 776.4	Mineral soil	2017–2018	Wang et al. (2022)

*Note:* Values are means ± standard deviation.

### 2.2 Land Use Intensity

There are several ways to develop a LUI index. One approach involves a quantitative method, which calculates the square root‐transformed sum of standardized intensity values for activities like mowing, fertilization, and grazing or other practices, as demonstrated by Neff et al. (2021). Alternatively, a qualitative assessment can be used, based on information about intervention types, as done in woodland systems (Beckmann et al. 2019) or the global land system (Chaudhary and Brooks 2018). A quantitative LUI index could integrate with structural equation modeling; however, it requires detailed information on frequency and intensity from each input variable (Neff et al. 2021). In this study, a qualitative LUI index method is more suitable considering that we did not have information on the frequency and intensity of management activities for each site.

We developed our LUI index based on the type of intervention following Beckmann et al. (2019). The interventions considered were artificial drainage or irrigation, the application of chemical fertilizers, and the management of understory vegetation. We also considered historical land use, recognizing that rehabilitation after disturbance or degradation often results in uniform age structure and affects ecosystem recovery processes (Mills et al. 2023). After collecting all relevant information, we classified LUI into three levels: low, medium, and high. Low LUI refers to primary forests that have remained undisturbed, with no sign of artificial drainage, irrigation, chemical fertilizers application, or understory management. Medium LUI consists of degraded ecosystems that have experienced past or historical logging activities, forest fire, or other disturbances and are currently managed or protected with the aims of restoration or rehabilitation. This category mainly consists of secondary forests that are undergoing natural regeneration and systematically planted to restore them to healthy conditions. High LUI is associated with plantation systems that frequently use chemical fertilizers, influenced by artificial drainage, and have regular understory management. These plantation sites include monoculture of oil palm, rubber and acacia. Further details on the LUI classification are available in Table 2. In SEA, high LUI sites are often established in two soil type; mineral soil and peat soil. Due to data limitation (Table S3) and the varying vegetation stages of plantation (e.g., SBW represents a young oil palm plantation with a relatively open canopy), peat soil sites were not included in the statistical analysis (see section 2.7). Apart from excluding sites on peat soil, we also emphasize that all sites on mineral soil were at mature stage (Table 1).

**TABLE 2:** Land use intensity classification based on intervention types adapted from Beckmann et al. (2019).

Land use intensity	Code	Dominant species	Age (years)	LAI (m^2^ m^−2^)	Canopy height (m)	Post disturbances	Drainage experienced	Chemical fertilizer usage	Regular clearing of understory	Note
Low (1)	LHP	Mixed species	Intact forest	6.2	40 – 50	No	No	No	No	
	MKL	*Shorea siamensis, Vitex peduncularis, Xylia xylocarpa*	30	2–3	30	No	No	No	No	
	PSO	*Dipterocarpaceae, Leguminosae, Burseraceae*	Intact forest	6.52	35	No	No	No	No	
	SKR	*Hopea ferrea Pierre*	Mature forest	3.5–4.0	35	No	No	No	No	
	KIF	*Shorea uliginosa, Calophyllum ferrugineum, Syzygium sp, etc*	—	—	32 ± 6	No	No	No	No	Could have some long‐term regional effects of hydrological management of surrounding plantations
	PUF	Mixed species	—	—	—	Yes	No	No	No	
Medium (2)	BKS	*Macaranga gigantea*	—	3.0	10.6	Yes	No	No	No	In 1998, this forest was highly degraded by forest fire and naturally regenerated.
	DFR	*Dipterocarpus intricatus, D. obtusifolius, D* *. tuberculatus* , *Shorea obtuse, and S* *. siamensis*	17–19 (2015)	4.55	7	Yes	No	No	No	Before 2005, this forest had been heavily degraded by illegal logging. There were remaining waterways for wood harvesting and two ponds near the EC tower.
	KDF	*Syzygium claviflorum, Shorea teysmanniana, Stemonurus secundiflorus, etc*	—	—	19 ± 6	Yes	Yes	No	No	The degraded site was selectively logged and drained in the late 1990s and early 2000s
	PDF	*Combretocarpus rotundatus, Cratoxylum arborescens, Buchanania sessifolia and Tetramerista glabra*	—	5.0	26	Yes	Yes	No	No	Peat swamp forests drained in the 1990s.
	PDB	Mixed species	—	—	—	Yes	Yes	No	No	Several times burned in 1997, 2002, 2009, and 2014
High (3)	JOP	*Elaeis guineensis* *Jacq*	19 (2022)	3.64	15	—	Yes	Yes	Yes	Fertilizers and herbicides were applied regularly.
	SBW	*Elaeis guineensis* *Jacq*	3–7 (2011)	—	8	—	Yes	Yes	Yes	Peat soil. Fertilizer was applied four times per year.
	KAP	*Acacia crassicarpa*	—	—	17 ± 6	—	Yes	Yes	Yes	Typically plantation rotation period is 4–5 years
	RFC	*Hevea brasiliensis*	27 (2013)	1.78–6.28	20	—	No	Yes	Yes	Fertilizer was applied two times per year in June and October.
	RFN	*Hevea brasiliensis*	19 (2019)	1.90–6.40	22	—	No	Yes	Yes	

*Note:* Age, LAI, and canopy height correspond to the year of data availability.

### 2.3 Estimation of Carbon Fluxes

Net ecosystem carbon exchange (NEE) was measured using the eddy covariance (EC) method and partitioned into gross primary productivity (GPP) and ecosystem respiration (Reco). The EC systems consisted of a three‐dimensional sonic anemometer‐thermometer and an infrared gas analyzer for CO_2_ and H_2_O concentration measurements, with most data recorded at 10 Hz and averaged at 30‐min intervals. Detailed sensor specifications and types can be found in the respective literature (see Table 1). Most sites utilized a similar marginal distribution sampling (MDS) gap‐filling method based on Reichstein et al. (2005). However, a site‐specific gap‐filling approach was used for the PSO site, adapting the techniques from Reichstein et al. (2005) to better fit the narrow temperature ranges, as explained in Kosugi et al. (2012). The percentage gap‐filled and root mean square error (RMSE) between original and gap‐filled NEE are available in Table 3. In addition, due to inconsistent data compiled from databases and repositories, such as some sites reporting both original and gap‐filled values (i.e., LHP, MKL, BKS, PSO, SKR, JOP, SBW), others only providing gap‐filled values (i.e., DFR, RFC, RFN, PDF, PDB, PUF), and some offering only daily values (e.g., KAP, KIF, KDF), not all sites have percentage gap‐filled and RMSE values. Furthermore, CO_2_ flux partitioning (into GPP and Reco) for some sites uses nighttime partitioning while others use daytime partitioning. The detailed flux partitioning method for each site can be found in Table S4 and respective literature in Table 1. Following the micrometeorological convention, negative NEE indicates carbon uptake, while positive NEE represents carbon release.

**TABLE 3:** Percentage NEE gap‐filled, root mean square errors (RMSE), and flux processing package for each site. For detailed year‐to‐year values, please refer to Table S5.

LUI	Ecosystem	Site	Measurement period	Percentage of gap‐filled data	RMSE (μ mol m^−2^ s^−1^)	Flux processing package and software
Low	Primary forest	LHP	2010–2019	80.22–93.25	6.03–9.37	ONEflux (Pastorello et al. 2020); EddyPro (LI‐COR, Lincoln, USA)
Low	Primary forest	MKL	2003–2004	73.08–74.89	8.51–34.44	ReddyProc R package (Wutzler et al. 2018); EddyPro (LI‐COR, Lincoln, USA)
Low	Primary forest	PSO	2003–2009	29.67–71.74	4.51–6.53	Kosugi et al. 2012
Low	Primary forest	SKR	2001–2003	17.88–36.95	2.12–2.52	ReddyProc R package (Wutzler et al. 2018); EddyPro (LI‐COR, Lincoln, USA)
Low	Primary forest	KIF^a^	2017–2022			ReddyProc R package (Wutzler et al. 2018); EddyPro (LI‐COR, Lincoln, USA)
Low	Primary forest	PUF^a^	2005–2017	65.28–75.42		Flux Calculator (Ueyama et al. 2012)
Medium	Secondary forest	KDF^a^	2001–2002			ReddyProc R package (Wutzler et al. 2018); EddyPro (LI‐COR, Lincoln, USA)
Medium	Secondary forest	DFR	2016–2020			ReddyProc R package (Wutzler et al. 2018); EddyPro (LI‐COR, Lincoln, USA)
Medium	Secondary forest	BKS	2015–2017	68.63	73.45	ReddyProc R package (Wutzler et al. 2018); EddyPro (LI‐COR, Lincoln, USA)
Medium	Secondary forest	PDF^a^	2001–2016	68.43–96.14		Flux Calculator (Ueyama et al. 2012)
Medium	Secondary forest	PDB^a^	2005–2016	34.42–68.09		Flux Calculator (Ueyama et al. 2012)
High	Oil palm	JOP	2014–2020	17.02–52.33	4.78–7.91	ReddyProc R package (Wutzler et al. 2018); EddyPro (LI‐COR, Lincoln, USA)
High	Oil palm	SBW^a^	2011–2014	58.31–76.14		Flux Calculator (Ueyama et al. 2012)
High	Acacia	KAP^a^	2016–2020			ReddyProc R package (Wutzler et al. 2018); EddyPro (LI‐COR, Lincoln, USA)
High	Rubber	RFC	2015–2018			ReddyProc R package (Wutzler et al. 2018); EddyPro (LI‐COR, Lincoln, USA)
High	Rubber	RFN	2017–2018			ReddyProc R package (Wutzler et al. 2018); EddyPro (LI‐COR, Lincoln, USA)

^a^ Represent sites on peat soil. An empty input means we could not access the original raw (half‐hourly) data, or an unsuccessful RMSE analysis.

### 2.4 Canopy Light Response

To assess the relationship between LUI and GPP, light use efficiency (LUE, μmol CO_2_ μmol photon^−1^) was estimated for each site. LUE is defined as the ratio of GPP to the total of photosynthetically active radiation (PAR) in an ecosystem (Gilmanov et al. 2007). Ecological LUE was estimated as:

(1) $$\mathrm{LU}E_{\mathrm{eco}}=\frac{\mathrm{GPP}}{\mathrm{PAR}}$$

The relationship between PAR and GPP was modeled as a canopy light response curve using a Michaelis–Menten hyperbolic function (Marino et al. 2010):

(2) $$\mathrm{GPP}=\frac{{\mathrm{GPP}}_{\max}\cdot \mathrm{PAR}}{k+\mathrm{PAR}}$$

where 𝐺𝑃𝑃_𝑚𝑎𝑥_ is the maximum GPP (μmol CO_2_ m^−2^ s^−1^), PAR (μmol m^−2^ s^−1^), and *k* is the light intensity at half maximum GPP (μmol m^−2^ s^−1^). Canopy light responses were differentiated according to wet and dry seasons, which were defined based on monthly precipitation averages. The three highest precipitation months constitute the wet season, and the three lowest are defined as the dry season.

### 2.5 Water Use Efficiency and Atmospheric Drought Index

Water use efficiency (WUE) was calculated to investigate the trade‐off between carbon assimilation and water use within an ecosystem (Law et al. 2002). The WUE (g C kg^−1^ H_2_O^−1^) is defined as the ratio between productivity (GPP, g C m^−2^ d^−1^) and evapotranspiration (ET, kg H_2_O m^−2^ d^−1^):

(3) $$\mathrm{WUE}=\frac{\mathrm{GPP}}{\mathrm{ET}}$$

While WUE reflects an ecosystem response to atmospheric conditions, the inherent WUE (iWUE) reflects more of an eco‐physiological state of the vegetation (Beer et al. 2009). The methods of iWUE incorporate vapor pressure deficit (VPD) and is defined as:

(4) $$\text{iWUE}=\frac{\mathrm{GPP}*\mathrm{VPD}}{\mathrm{ET}}$$

The different formulations of WUE illustrate the response of ecosystem carbon and water balance to climate change and water management practices (Niu et al. 2011).

To investigate severe to extreme atmospheric drought and wet events, the standardized precipitation evapotranspiration index (SPEI), developed by Vicente‐Serrano et al. (2010), was used on a monthly timescale. The SPEI considers the climatic water balance, which is defined as the difference between precipitation and potential evapotranspiration (PET), and thus accounted for both water supply and atmospheric water demand (Vicente‐Serrano et al. 2015). PET was calculated according to the Thornthwaite method (Thornthwaite 1948). A log‐logistic probability distribution was used to standardize the climatic water balance for comparison across different spatial and temporal scales (Beguería et al. 2014; Vicente‐Serrano et al. 2010). Then, drought levels were categorized based on SPEI values, as shown in Table 4, following Li et al. (2015).

**TABLE 4:** Classification of atmospheric water scarcity based on SPEI value.

SPEI value	Category
SPEI ≤ −2.00	Extreme drought
−2.00 < SPEI ≤ −1.50	Severe drought
−1.50 < SPEI ≤ −1.00	Moderate drought
−1.00 < SPEI < 1.00	Normal
1.00 ≤ SPEI < 1.50	Moderate wet
1.50 ≤ SPEI < 2.00	Severe wet
SPEI ≥ 2.00	Extreme wet

### 2.6 Relative Extractable Soil Water

To further investigate the dryness of the soil, the relative extractable soil water (REW) index was calculated, allowing a comparison of soil moisture across sites. REW indicates soil water availability in the rooting zone (Bréda et al. 2006). REW was estimated as follows:

(5) $$\mathrm{REW}=\frac{{\mathrm{SWC}}_{\mathrm{day}}-{\mathrm{SWC}}_{\min}}{{\mathrm{SWC}}_{\max}-{\mathrm{SWC}}_{\min}}$$

where the ${\mathrm{SWC}}_{\mathrm{day}}$ is the daily average of soil water content. ${\mathrm{SWC}}_{\max}$ and ${\mathrm{SWC}}_{\min}$ are the maximum and minimum soil water content for the rooting zone over the entire data set. REW ranges from 0.0 (representing low soil water content) to 1.0 (high soil water content). According to Granier et al. (2007), a REW value lower than 0.4 indicates a potential stress on the water supply, triggering stomatal regulation in the vegetation.

### 2.7 Statistical Analysis

In this study, we focused our statistical analysis of LUI to sites which were established on mineral soil due to inconsistent data on the peat soil site. There are inconsistent conditions of sites located in peat soil, such as different vegetation age (e.g., SBW with young oil palm and PDB is a burned peat swamp dominated by understory), and some sites (e.g., KAP, KDF, KIF) only provide daily values. Meanwhile, all mineral soil sites are at a mature stage. Then, we conducted the statistical analysis with daily values (derived from half‐hourly data) as input parameters. First, we utilized a steel‐dwass test to investigate the impact of LUI gradient on carbon and water fluxes, with a significant level of 0.05 as a threshold. This analysis aims to give an overview on the direction of the trade‐offs on carbon and water fluxes.

Second, we applied a linear mixed‐effects model (LMEM) to assess the effects of meteorological parameters as fixed effects (e.g., air temperature (Tair), photosynthetic active radiation (PAR), REW, relative humidity (RH), precipitation, vapor pressure deficit (VPD)) on carbon fluxes (e.g., NEE, GPP, Reco), LUE, and WUE, while accounting for random effects (e.g., site characteristic) (Bates et al. 2015; Monsalves et al. 2020). This analysis aims to test whether random effect from site characteristic was higher than the effect of LUI. In random effects analysis, we used residual variance (*σ* ^2^) to indicate variability in each response of parameters that were not accounted for by the fixed effects. ${Ʈ}_{00}$ site represents the variance attributed to random effects from site in respect to carbon fluxes, WUE, and LUE. Interclass correlation (ICC) indicates the proportion of the total variance attributed to site‐level. A higher ICC value means a stronger influence of site characteristics on certain responses. Marginal and conditional *R* ^2^ provide how much variance is explained by fixed effect alone (marginal) and by both fixed and random effects (conditional).

Third, we also implemented the SHAP (Shapley additive explanations) metric to interpret the main factors contributing to NEE and ET in different LUI (Takamura et al. 2023). SHAP serves as a method to enhance our understanding of the relationships between individual drivers of the predicted model (Batunacun et al. 2021). The SHAP method assigns a value to all input parameters for a specific model prediction, representing a measure of their contribution to the model output (Takamura et al. 2023). For the SHAP analysis, we set daily values of PAR, VPD, Tair, friction velocity (uStar), REW and precipitation as input parameters and processed using a tree‐based machine‐learning algorithm, the gradient‐boosted regression tree model (Chen and Guestrin 2016). Mean absolute SHAP values for the input parameter on each site then combined based on their respective LUI categories, indicated the main controlling factors for ET and NEE in each LUI gradient. All descriptive and statistical analyses were performed with the statistical program R version 4.4.1 (R Core Team, 2025).

## 3 Results

### 3.1 Trade‐Off of LUI Gradient on Carbon‐Water Coupling Dynamics

**FIGURE 2:**
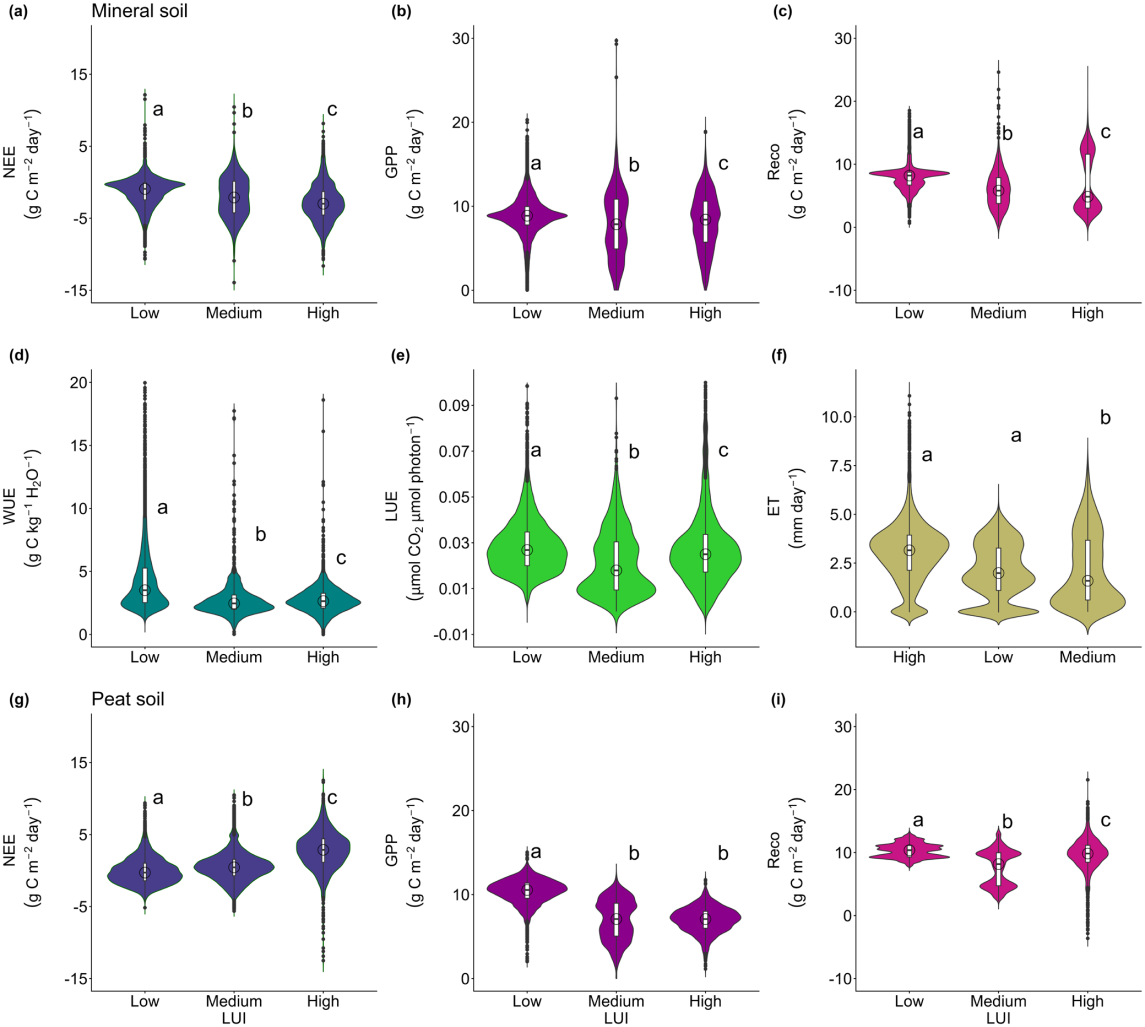
Impact of LUI on carbon and water fluxes on mineral and peat soil. LUI class refers to Table 2. Letters (a, b) near the top of each plot indicate significant population distribution differences between land use intensities (Steel‐Dwass test; *p* < 0.05). The box plot elements represent the median (open circle) and interquartile range of each LUI, while the wider section on the violin plot represents a higher probability of the data distribution. For peat sites, only carbon flux data were available.

High LUI sites on mineral soil exhibit greater carbon uptake compared to low LUI sites, but it led to a lower WUE as a trade‐off (Figure 2). It is important to note that high LUI in this category excludes factors such as harvest, establishment stage, and rotation periods, which often lead to large carbon sources. Although high LUI improves carbon absorption, the daily variability of carbon fluxes, particularly GPP and Reco, tends to be larger with higher LUI (Figure 2b,c). In contrast, WUE significantly declines as LUI increases (*p* < 0.05). In line with WUE trends, annual ET also increases along with the LUI gradient, but with higher daily variability. These results indicate trade‐offs between carbon and water fluxes at different LUI.

**FIGURE 3:**
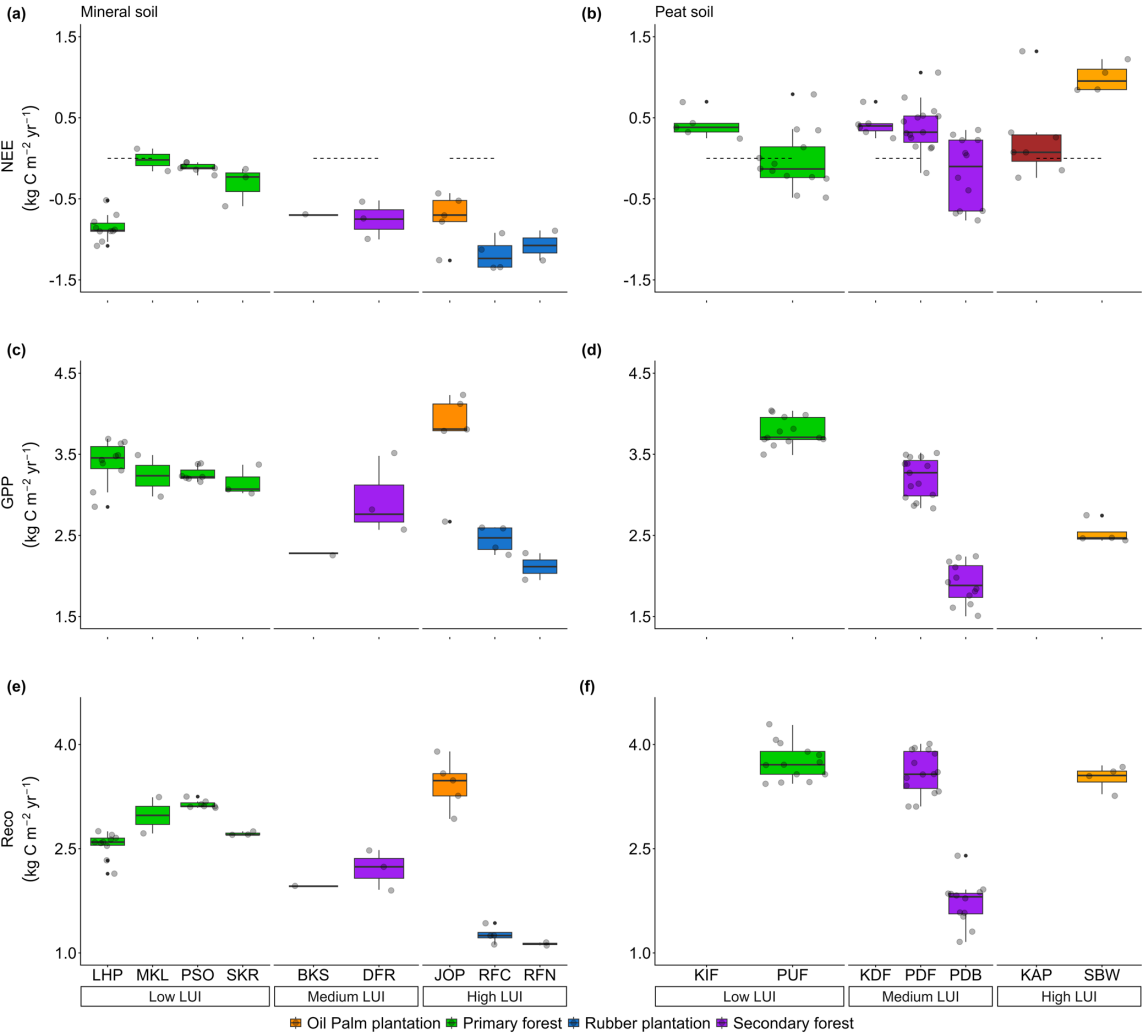
Mean annual carbon fluxes according to a LUI gradient in Southeast Asia: (a) NEE, (b) GPP and (c) Reco. The horizontal line inside the box represents the median value, while the box shows the interquartile range of the data distribution and points as an outlier. Detailed year to year carbon fluxes on each site can be found in Table S6.

Looking at more details on carbon fluxes, high LUI demonstrated stronger capacity for absorbing carbon compared to medium and low LUI, as indicated by more negative values of NEE (Figure 3). The stronger C uptake in high LUI is closely linked to the species selection with fast‐growing characteristics (e.g., oil palm‐ JOP) (Figure 3c) which are managed in purpose to enhance their productivity, and the controlled understory (e.g., rubber plantation‐RFC and RFN), thereby led to lower Reco (Figure 3e). However, a young oil palm plantation (SBW) on peat soil acted as net carbon sources. This result highlights that vegetation type and site characteristics in monocultures were key determinants of NEE. This strong characteristic also emphasized by mean annual NEE at two rubber plantations was in a similar range (mean ± SD: −1.07 ± 0.26 kg C m^−2^ year^−1^ on RFN and −1.19 ± 0.20 kg C m^−2^ year^−1^ on RFC, respectively), despite having different rainfall regimes (Table 1, Figure 1). Moreover, oil palm plantations showed varying carbon dynamics according to soil type and vegetation age. For example, oil plantation on mineral soil (JOP: 19 years old) acted as a carbon sink (−0.74 ± 0.32 kg C m^−2^ year^−1^), whereas the young plantation on peat soil (SBW: 3–7 years old) was a large carbon source (0.98 ± 0.19 kg C m^−2^ year^−1^).

**FIGURE 4:**
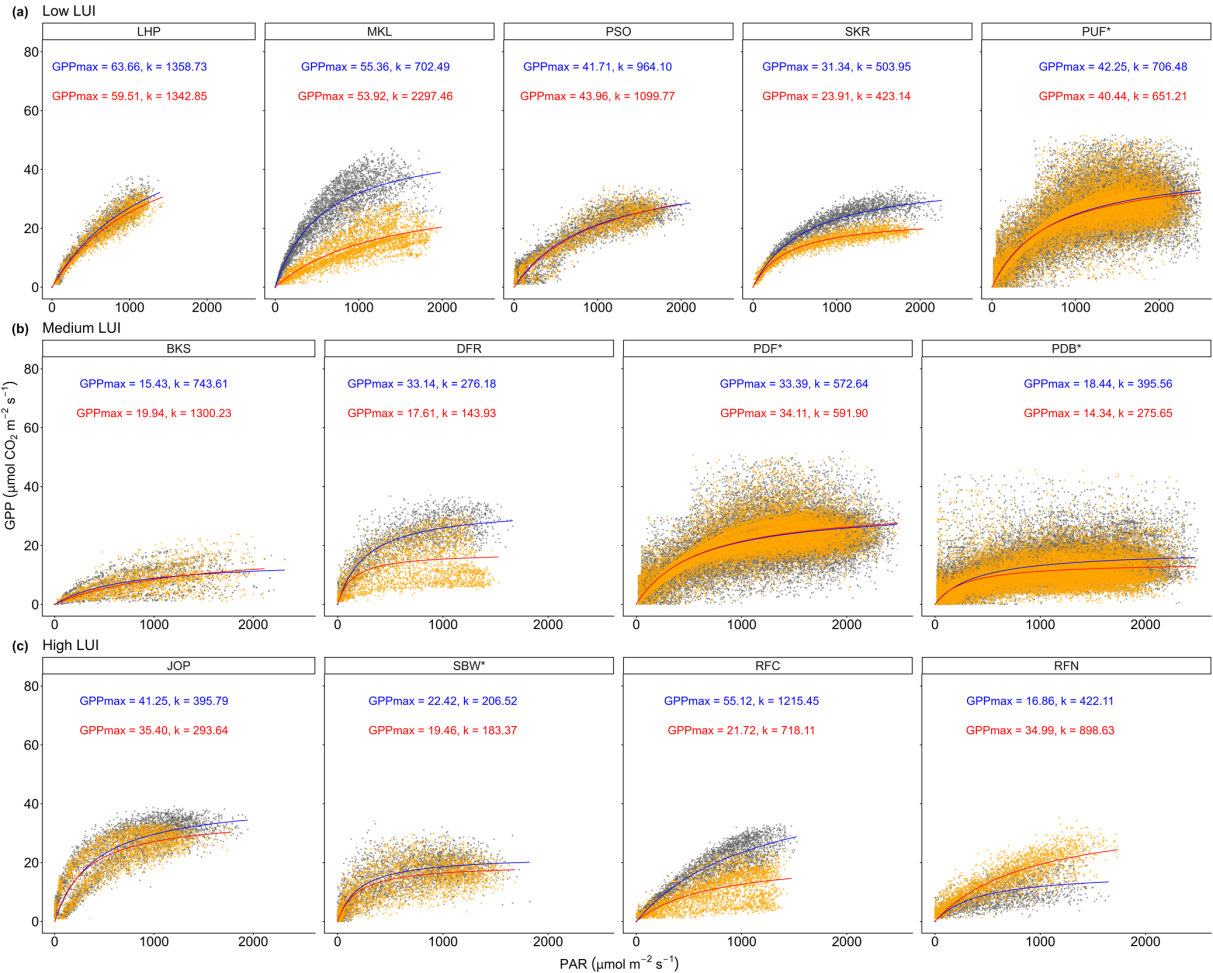
Canopy light response curves are separated into dry (orange scatter points) and wet seasons (grey scatter points) across different LUI. Regression curves were fitted by a rectangular hyperbola model with GPPmax (μmol m^−2^ s^−1^) and k (μmol m^−2^ s^−1^) shown in each panel. Blue and red lines refer to light response curves for wet and dry seasons, respectively. There are no light response curves for Kampar sites (e.g., KIF, KDF, KAP) due to inconsistent data characteristics (see Table S3).

The importance of vegetation and site characteristics in high LUI further highlighted by a larger interannual variability of GPP and Reco compared other LUI (Figure 4). Although both oil palm established on mineral and peat soil had similar mean annual Reco (3.43 ± 0.36 kg C m^−2^ year^−1^ for JOP and 3.49 ± 0.22 kg C m^−2^ year^−1^ for SBW, respectively), oil palm on mineral soil (JOP) showed a higher GPP compared to site on peat soil (SBW) (*p* < 0.05). On the other hand, rubber plantations demonstrated the lowest annual GPP and Reco for all sites (*p* < 0.05). Those CO_2_ fluxes variability emphasize the importance of vegetation type and site‐specific characteristics in high LUI. However, it is crucial to note that the limited time frame of this study did not allow capturing changes in carbon fluxes over the full rotation period of the plantations.

In contrast, low LUI sites (i.e., primary forest) showed less interannual variability in carbon fluxes than sites on high LUI (*p* < 0.05), indicating higher ecosystem stability. While the strength of the carbon sink on low LUI (NEE: −0.02 to −0.85 kg C m^−2^ year^−1^) was generally lower than on high LUI on mineral soil, the mean annual GPP (3.15 to 3.39 kg C m^−2^ year^−1^) and Reco (2.55 to 3.14 kg C m^−2^ year^−1^) were relatively comparable. Meanwhile, medium LUI had slightly higher interannual variability of GPP (2.28 to 3.15 kg C m^−2^ year^−1^) and Reco (1.96 to 2.72 kg C m^−2^ year^−1^) compared to low LUI. The high interannual variability on medium LUI possibly relates to legacy effect from past disturbance and successional regrowth with a higher sensitivity to drought events (Figure 6a). The high variability of carbon fluxes at medium LUI is more significant at peat soil, as shown by PDF and PBD sites. It indicates that legacy effects on peat soil potentially last longer than on mineral soil. These results further emphasize the important role of vegetation type and soil properties along with LUI gradient in determining carbon dynamics.

### 3.2 Canopy Light Response Curve Across LUI Gradient

The strong C uptake in high LUI is also reflected in the canopy light response curve (Figure 4), which shows that high LUI required less PAR to achieve half‐maximum GPP. However, the curves were faster in demonstrating flattening, indicating that carbon assimilation did not further increase significantly after reaching the half‐maximum GPP. This pattern highlights the strong growth potential of fast‐growing monoculture even under cloudy conditions. However, the faster in reaching half‐maximum GPP on high LUI did not necessarily lead to higher LUE compared to low LUI, although the difference was small (*p* < 0.05, Table S6). For example, in order to reach half GPP_max_, JOP required PAR 395.8 μmol m^−2^ s^−1^ and 293.6 μmol m^−2^ s^−1^ during wet and dry seasons, respectively. In contrast, as a primary forest site, LHP required significantly higher PAR values to reach half GPP_max_ (1358.7 and 1342.8 μmol m^−2^ s^−1^ during wet and dry seasons, respectively). Meanwhile, medium LUI required a wide range of PAR reach of half of GPP_max_ (from 143.93 to 1300.23 μmol m^−2^ s^−1^), emphasizing the large magnitude of the ecosystem in a transitional phase from disturbed into more stable. It is also worth mentioning that the LUE and canopy light response curve are closely correlated to ecosystem age and species traits, especially in high LUI. For example, JOP had daily LUE average of 0.039 ± 0.028 μmol CO_2_ μmol photon^−1^, while RFC and RFN have similar LUE at 0.022 ± 0.013 and 0.022 ± 0.012 μmol CO_2_ μmol photon^−1^, respectively.

### 3.3 WUE and Severe‐Extreme Drought Sensitivity Across LUI Gradient

**FIGURE 5:**
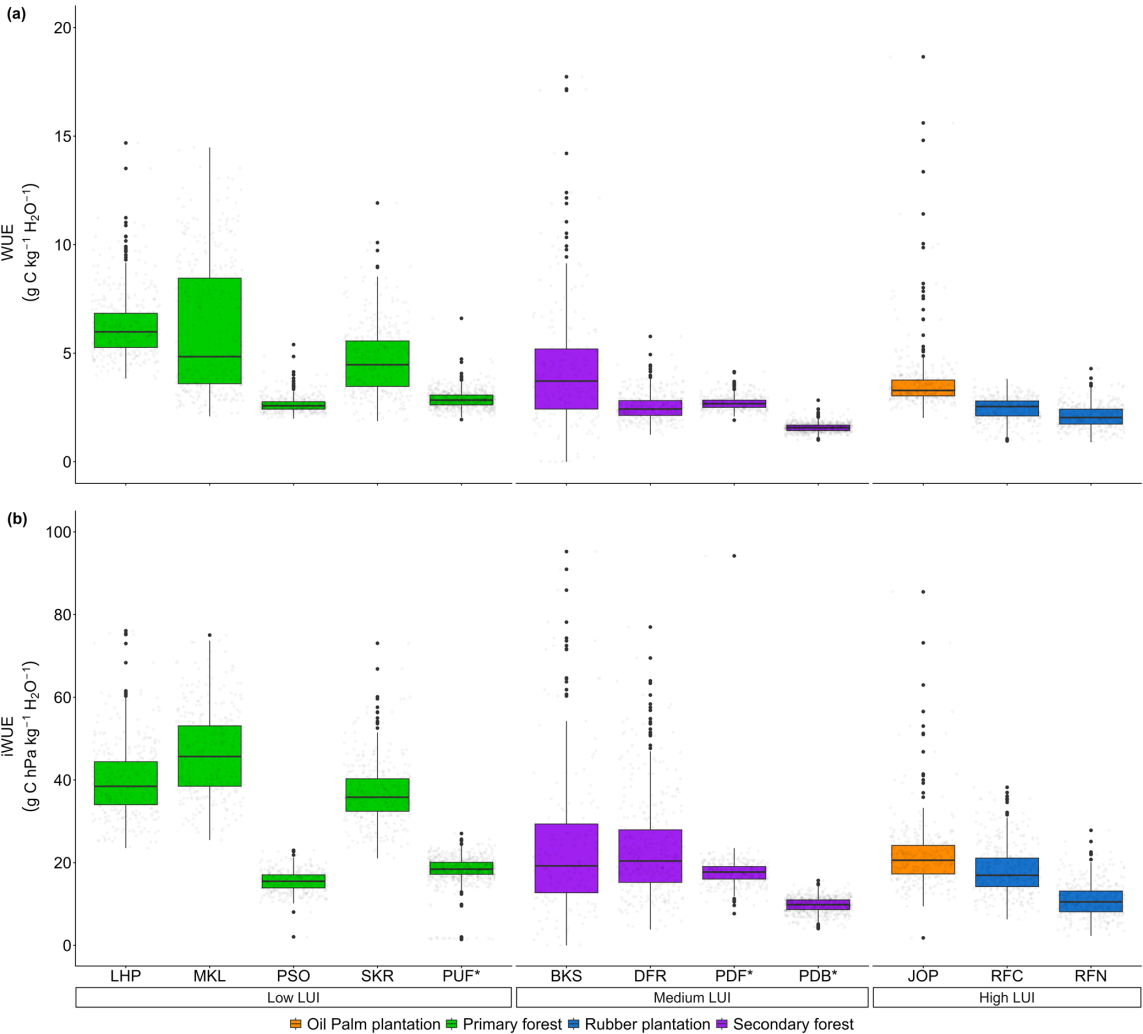
Mean daily WUE and iWUE across a LUI gradient. The horizontal line inside the box represents the median value, while the box shows the interquartile range of the data distribution and points as an outlier. * indicated the site located on peat soil. Detailed year to year WUE and iWUE on each site can be found in Table S6. There are no WUE and iWUE for Kampar sites (e.g., KIF, KDF, KAP) and SBW due to inconsistent data characteristics (see Table S3).

In exchange for enhanced C uptake (indicated by more negative NEE) at high LUI, this LUI category performed lower in terms of WUE (*p* < 0.05) (Figure 5). Low LUI exhibited a higher WUE than high LUI (*p* < 0.05), although WUE values were not significantly different with medium LUI (*p* > 0.05). Low LUI demonstrated greater daily variability in WUE, ranging from (mean ± SD) 2.63 to 6.50 g C kg^−1^ H_2_O^−1^, compared to high LUI, ranging from 2.08 to 3.53 g C kg^−1^ H_2_O^−1^ (Table S6). In addition, when incorporating the vegetation component to WUE through VPD, there is no significant difference in mean daily variability between WUE and iWUE. The highest iWUE variability consistently occurred at low LUI, followed by medium and high LUI (*p* < 0.05).

**FIGURE 6:**
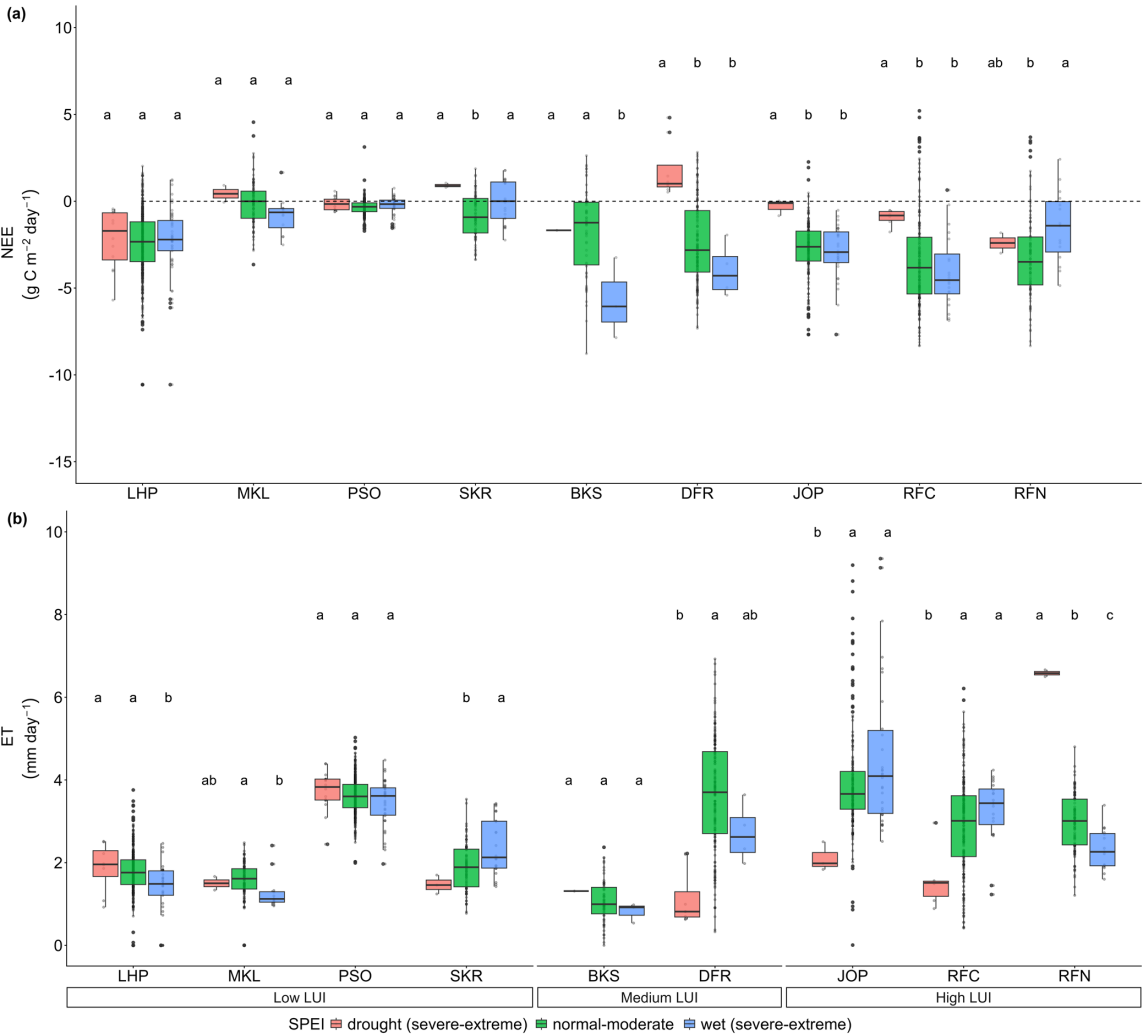
Daily NEE and ET variability based on SPEI value with a threshold of 1.5, referring to severe‐extreme conditions of wet and drought events (see Table 3) across different LUI on mineral soil sites. Small letters correspond to significantly different groups (*α* = 0.05) from post hoc Tukey LSH; the same letter means not significantly different. The horizontal line inside the box represents the median value, while the box shows the interquartile range of the data distribution and points as outlier.

The variability of carbon fluxes in high LUI is also linked to stronger sensitivity to water scarcity as shown in Figure 6a. Under severe to extreme events (where SPEI > 1.5 indicating severe wet or SPEI < −1.5 means severe drought), the daily NEE variability at medium and high LUI was significantly affected by water scarcity (Figures 6 and S6). Medium LUI showed the largest disparity of daily NEE between severe‐extreme drought (ranging from −1.68 to 1.79 g C m^−2^ day^−1^) and wet conditions (−5.72 to −0.92 g C m^−2^ day^−1^). On the other hand, low LUI demonstrated a smaller disparity between severe‐extreme wet (−2.30 and −0.01 g C m^−2^ day^−1^) and drought conditions (−2.19 and 0.91 g C m^−2^ day^−1^). This result further highlights that LUI gradients increase the ecosystem sensitivity to water scarcity, thus resulting in high interannual variability of carbon fluxes (Figure 4).

Additionally, the stronger response of ecosystem sensitivity to water scarcity showed in ET between severe to extreme wet and drought events following LUI gradient (Figure 6b). At high LUI sites, ET varied from 2.33 to 4.58 mm day^−1^ during severe‐extreme wet events, while a larger range was found during severe‐extreme drought events (ranging from 1.59 to 6.58 mm day^−1^). In contrast, low LUI showed a lower ET difference between severe‐extreme wet event (ranging from 1.29 to 3.43 mm day^−1^) and severe‐extreme drought event (1.47 to 3.73 mm day^−1^). This result also points out that high LUI not only had lower WUE but also showed to be more sensitive to drought events compared to low LUI.

### 3.4 The Difference of Meteorological Drivers Across LUI Gradients

Along with LUI gradient, site characteristics also play an important role in regulating the trade‐off between carbon fluxes and WUE as shown in Table 5. The random effects of site characteristics showed moderate impact in NEE (*σ* ^2^: 5.06 ± 2.25, ICC: 0.48) with high impact in Reco (*σ* ^2^: 8.34 ± 2.88, ICC: 0.64) and very high on LUE (*σ* ^2^: 12.22 ± 3.49, ICC: 0.91). This emphasizes the site characteristics and species selection in high LUI play an important role in affecting the LUE. Moreover, LUE has the highest ICC also suggesting strong site‐level effects. The LUI gradient also had a stronger influence on WUE than the random effect from site, as shown with the lower ICC (0.23) and with a negative relationship. Those lower ICC and negative relationships emphasize that high LUI led to lower WUE. Then, the low ratio between marginal and conditional R^2^ in following LUI gradients further highlights the complex interplay of meteorological parameters and ecosystem services (e.g., carbon fluxes, WUE, and LUE) along with LUI gradients.

**TABLE 5:** Fixed and random‐intercept linear mixed effects models of meteorology parameters with carbon fluxes, water and energy efficiency in SEA forests and plantations on mineral soil (estimates ± std. error).

	NEE	GPP	Reco	WUE	LUE
(intercept)	1.28 ± 1.25	9.48 ± 0.82***	8.84 ± 1.66***	6.23 ± 0.84	0.01 ± 0.02
Fixed effects					
REW	0.01 ± 0.02	−0.11 ± 0.03***	0.03 ± 0.02	−0.03 ± 0.03	−0.0003 ± 0.012
Tair	0.14 ± 0.02***	0.54 ± 0.03***	0.36 ± 0.02***	0.18 ± 0.03***	−0.0008 ± 0.013
PAR	−1.22 ± 0.07***	0.91 ± 0.08***	1.04 ± 0.07***	−2.54 ± 0.11***	0.0066 ± 0.041
RH	−0.40 ± 0.03***	0.96 ± 0.03***	0.47 ± 0.03***	0.40 ± 0.04***	−0.0228 ± 0.015
VPD	−0.02 ± 0.02	−0.11 ± 0.02***	−0.12 ± 0.02***	−0.06 ± 0.02***	0.0020 ± 0.009
Precipitation	0.19 ± 0.02***	−0.12 ± 0.02***	−0.12 ± 0.02***	0.03 ± 0.03	−0.0079 ± 0.011
LUI[High]	−0.17 ± 1.65	−2.75 ± 1.09	−2.31 ± 2.20	−3.85 ± 1.19***	0.0300 ± 2.855
LUI[Medium]	−0.50 ± 1.76	−1.18 ± 1.17	−1.99 ± 2.36	−1.82 ± 1.19	3.5080 ± 2.855
Random effect					
*σ* ^2^	5.06 ± 2.25	6.50 ± 2.55	4.66 ± 2.16	7.06 ± 2.56	1.14 ± 1.06
Ʈ_00_ Site	4.67 ± 2.16	2.04 ± 1.43	8.34 ± 2.88	2.12 ± 1.45	12.22 ± 3.49
ICC	0.48	0.24	0.64	0.23	0.91
N_site_	10	10	10	9	9
Observations	10,720	10,541	10,484	8747	9135
Marginal *R* ^2^/Conditional *R* ^2^	0.030/0.496	0.135/0.342	0.070/0.667	0.246/0.420	0.145/0.927

*Note:* *p*‐Value: *p < 0.05, **p < 0.01, ***p < 0.001.

**FIGURE 7:**
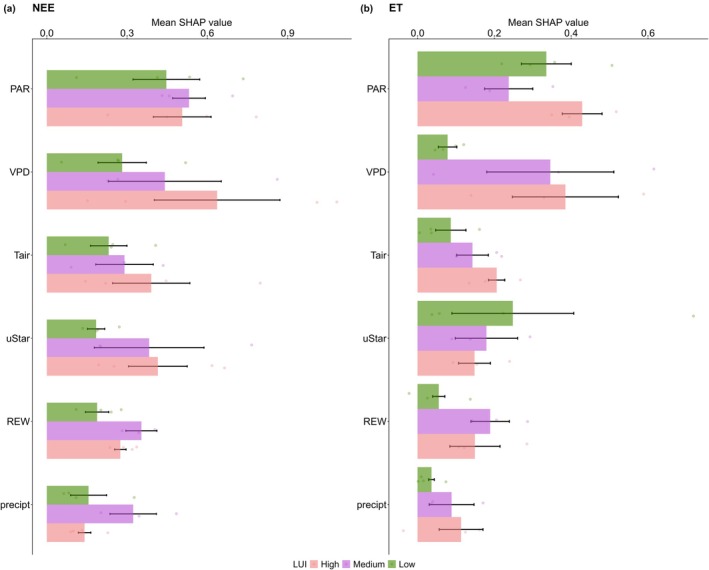
Mean SHAP value of meteorological drivers on (a) NEE and (b) ET across LUI gradient. Dots represent the SHAP value of each site. PAR refers to photosynthetic active radiation, VPD is vapor pressure deficit, uStar represents friction velocity, REW denotes relative extractable water, Tair signifies air temperature, and precipt indicates precipitation.

Strengthening the substantial impact of meteorological variables on carbon fluxes (Table 5), the SHAP analysis assessed the relative importance of those factors on NEE and ET across the LUI gradient (Figure 7 and Figures S2 and S3). At low LUI, PAR and VPD were identified as the primary predictors of NEE. Meanwhile, at high LUI, VPD emerged as the most important predictor of NEE. These results captured the importance of atmospheric drought in regulating carbon uptake following the LUI gradient. Similar to SHAP on NEE, PAR, and VPD were key variables influencing ET following the LUI gradient. At low LUI, PAR and friction velocity were identified as the main drivers of ET. Whereas, at high LUI, ET was more strongly influenced by PAR and VPD. At medium LUI, the role of VPD on ET was further strengthened as the most influential driver. These findings suggest the importance of VPD in controlling ET and highlight the potential risk of atmospheric drought affecting both NEE and ET in response to the LUI gradient.

## 4 Discussion

### 4.1 Low vs. High LUI: What Are the Gains and Trade‐Offs Regarding Carbon Uptake and Water Use?

Our analysis reveals that high LUI with fast‐growing species, such as oil palm and rubber plantations, demonstrated higher carbon uptake compared to low and medium LUI when established on mineral soil (Figures 2 and 3). However, this conclusion focuses only on mature plantations without accounting for carbon losses during harvesting, establishment, and the full rotation cycles. Stronger carbon sink in high LUI is largely due to the low Reco in rubber plantations (RFC and RFN), whereas oil palm plantation (JOP) showed similar NEE and Reco to medium LUI or even higher on peat soil (SBW). The variations in NEE among different LUI are closely linked to factors such as fertilization levels, soil type, and ecosystem age. For example, the oil palm plantation in JOP received 196 kg N ha^−1^ year^−1^, while SBW applied Urea (74–147 kg N ha^−1^ year^−1^), rock phosphate (7–9 kg P ha^−1^ year^−1^), and muriate of potash (239–311 kg K ha^−1^ year^−1^) (Kiew et al. 2020; Meijide et al. 2020). Meanwhile, rubber plantations in RFN and RFC were fertilized with 180 g N tree^−1^ year^−1^, 80 g P tree^−1^ year^−1^, and 170 g K tree^−1^ year^−1^ (Wang et al. 2022). Besides these fertilizers, herbicide application also contributes to chemical input to the soils. These practices ensure nutrient availability for the vegetation but can reduce the microorganism populations, increase nutrient leaching, and change soil acidity in the long term (Clough et al. 2016).

There is also an open discussion on how to address greenhouse gases emission other than CO_2_ from plantations. Methane (CH_4_) and nitrous oxide (N_2_O) emissions from plantations treated with fertilizer and herbicide on peat or mineral soils can substantially contribute to radiative forcing (Chen et al. 2020; Wang et al. 2022). Sustainable plantation management should consider these non‐CO_2_ emissions as part of a life cycle assessment as high fertilizer application can lead to substantial N_2_O emissions (Meijide et al. 2020; Stiegler et al. 2023). Young plantations experience surface heating due to reduced evaporative cooling, leading to increased sensible heat fluxes, which is then diminishing as the plantation ages (Meijide et al. 2017; Sabajo et al. 2017).

Furthermore, plantations operate on a rotation system, which is typically cutting down the vegetation and replanting once they exceed the optimal phase. The rotation period varies depending on the species and management practices, normally occurring every 25–30 years for oil palm (Meijide et al. 2020), 4–5 years for Acacia (Deshmukh et al. 2023), and 30 years for rubber plantations (Chen et al. 2025). Maintaining plants in their optimal phase generally leads to higher carbon uptake rates, particularly when adding fertilizer, compared to primary or secondary forests which are naturally grown without any human interventions. However, when these plantations are cut down and replanted, they often become carbon sources (Meijide et al. 2020). Therefore, to make a comprehensive comparison, it is essential to monitor the full rotation cycle of plantation systems, which is rarely done. The full rotation cycle captures the balance amount of the carbon emitted during the establishment stage and absorbed at mature age.

As post‐disturbance, C dynamic in the early stage or logging of plantations often led to persistent and high levels of soil respiration, potentially turning the ecosystem to a C source for some time (Mills et al. 2023; Riutta et al. 2021). Throughout an entire rotation period, plantations may become net carbon sources, as regular harvests remove stored carbon (e.g., in palm oil or latex), which can lead to long‐term carbon losses on site (Meijide et al. 2020). Intensive land management practices, such as fertilization, herbicide application, and thinning, led to reduced competition and created optimal conditions to boost productivity (Beckmann et al. 2019). This intensive management, however, raises questions about the how long plantations can effectively serve as net carbon sinks and how about unwanted side effects from the intensive management, such as loss of biodiversity or soil acidification (Clough et al. 2016). Therefore, it is essential to conduct comprehensive and full rotation calculations in plantations over the long term to balance the strength of the carbon sink during the mature stage, carbon source during the initial stage, along with assessing drought risk.

Although plantations on mineral soil have high carbon uptake, they are less efficient in their water use, indicating a clear trade‐off between carbon and water fluxes. This is illustrated by the higher ET and lower WUE values at high LUI sites compared to the low LUI systems (Figures 3 and 5). The high‐water demand can have significant hydrological impacts on the landscape. For example, young oil palm plantations transpire less water than primary forests, but lead to increased surface temperature and reduce soil moisture (Sabajo et al. 2017). In contrast, mature plantations increase ET, reproducing a similar surface temperature as forests, yet they consume more water, reduce water recharge, and increase the susceptibility to water stress (Manoli et al. 2018). Transpiration rates in plantations, such as oil palm plantations, are high or even higher compared to tropical forests (Röll et al. 2015), with potentially 18%–27% higher transpiration and 15%–20% higher ET than forests (Fan et al. 2019), indicating high water use in these ecosystems. Further, this can lead to water scarcity at the ecosystem level, as shown by Merten et al. (2016), who found a significant decline in water availability due to the rapid change in land use from forest to monoculture landscape, including rubber, and oil palm landscape in Indonesia. Given future climate change conditions, these trade‐offs need to be considered to evaluate species characteristics and suitable place selection for sustainable land use and hydrological systems (Levia et al. 2020).

In contrast, primary forests (low LUI) demonstrated a higher WUE, thereby strengthening the ecosystem's resilience to water scarcity. Primary forests fulfill their role as “sponge and pump” in hydrological functions better than plantations, with the “sponge” effect referring to the enhancement of water infiltration and moisture retention enhancement due to soil through the physical properties of the soil, while the “pump” effect regulating water vapor release through transpiration (Bruijnzeel 2004). Plantations such as mature oil palm have pump‐like properties to maintain productivity; however, they do not have sponge‐like properties that make it use more water than store water (Kristanto et al. 2022). These mechanisms increase the adaptability and resilience of the low LUI ecosystems by ensuring water availability in the landscape and supporting both carbon uptake and water security during the dry season, and reducing the risks of flooding during the wet season (Merten et al. 2016, 2020).

In summary, while plantations with high LUI provide high short‐term productivity and carbon sequestration under ideal conditions, these benefits are tempered by intensive resource requirements, higher non‐CO_2_ greenhouse gas emissions, and potential carbon loss over rotation cycles. Primary forests, with their greater resilience, higher WUE, and broader ecosystem services, contribute to a more sustainable carbon and water balance, highlighting their critical role in maintaining ecosystem stability and resilience in Southeast Asian landscapes.

### 4.2 Specific‐Species Characteristics Dependencies on Plantations and the Benefit of High Heterogeneity in Forests

In mature plantation systems, specific‐species characteristics play a crucial role in controlling the NEE. This is demonstrated by rubber plantations exhibiting lower GPP compared to primary forests, yet their Reco is significantly lower (Figure 3). This lower Reco outweighs the small GPP, making this ecosystem act as a carbon sink. This outcome is attributed to the reduction in belowground biomass and soil microbial activity due to the application of fertilizers and regular understory management (Clough et al. 2016; Wang et al. 2022). On the other hand, oil palm on mineral soil shows high Reco alongside high GPP, which collectively result in more negative NEE (strong C uptake). These examples emphasize the role of specific‐species traits in plantations. This factor also regulates the high uncertainty of carbon fluxes on high LUI across different species.

The dependency of plantations on species characteristics is also supported in the canopy light response curve. The initial sharp rise and subsequent flattening of the light response curve in plantations correspond to the traits of fast‐growing species with high photosynthetic capacity, thus increasing the GPP. Although this depends not only on specific species traits but also site characteristics, plantations may increase carbon uptake for a short period. In high LUI sites, although daily LUE was lower compared to low LUI (Figure 7), the canopy response curve shows that less PAR is needed to achieve GPP maximum. Light absorption through tree canopy generally follows a logarithmic pattern, which can vary based on species composition, site fertility, canopy structure, and silvicultural treatments (Binkley et al. 2013).

On the other hand, primary forests have higher WUE (Figure 5) and less interannual variability of NEE (Figure 3) than plantations. Those factors are linked to a higher biomass accumulation in primary forests than plantation systems, as a benefit from a high heterogeneous ecosystem (Guillaume et al. 2018). Biomass productivity, associated with above and belowground biomass, soil carbon, and litterfall, also improves other aspects of ecosystem functions and services such as biodiversity, pollination, and microclimate (Clough et al. 2016; Grass et al. 2020; Meijide et al. 2018). High biomass productivity ensures ecosystem resilience to water stress and stability, even though the rate of short‐term carbon absorption is not as high as in plantations (Aguilos et al. 2018). Stand biodiversity and biological control mechanisms play an important role in the long‐term WUE (Aguilos et al. 2018). Structural changes in vegetation, diversity in leaf phenology, and changes in surface conductance individually or in combination positively influence annual water availability (Costa et al. 2010). Therefore, primary forests are often more stable when unfavorable climate conditions occur compared to other land uses (Ma'rufah et al. 2022). In contrast to homogeneous plantation systems, heterogeneous forests and their species richness promote greater stability and resilience to environmental change (Levia et al. 2020).

However, there is a crucial challenge in disentangling the inherent effect between forest and plantation with LUI. Inherent differences in species composition between forest and plantation also influence ecosystem processes independently of management intensity. LUI in this study did not isolate the influence of management intensity from confounded factors such as species and ecological differences. A study done by Himes et al. (2020) suggested that single ecosystem services were maximized by monocultures (in this study reflected by higher C uptake). However, there is a legacy effect from land management practices on other ecosystem processes such as the functioning of the aboveground communities (e.g., species composition) with implications for belowground ecosystem services (e.g., nutrient cycling, water holding capacity) (Mejía et al. 2024).

### 4.3 Site Specific Importance and Global Comparison in Tropical Regions

Apart from the LUI gradient, the influence of site characteristics such as ecosystem structure, soil type, and species composition also affects the magnitude and direction of carbon fluxes (Table 4). Tree size and ecosystem age are also important factors in ecosystem carbon cycling (Kunert and Aparecido 2024). Ecosystem structure has a substantial impact on productivity and determines the carbon budget (Rödig et al. 2018). In particular in SEA, soil type (e.g., mineral or peat soil) is a crucial driver in determining the carbon cycle and WUE. This can be seen by secondary forest (e.g., DFR) and oil palm plantations (e.g., JOP) on mineral soil acting as carbon sinks, while secondary forests (e.g., PDF) and oil palm plantations (SBW) are carbon sources.

In comparison with other tropical regions globally, low LUI sites showed a relatively similar range of NEE and WUE between SEA, South America, and Africa regions (Tables S1 and S2). However, the variability of carbon fluxes and WUE is larger on sites with high LUI. The high variability of carbon fluxes in SEA is closely related to soil type while for other regions vegetation structure and species characteristics are more dominant. For example, annual mean NEE at low LUI in SEA ranged from −0.85 to −0.02 kg C m^−2^ year^−1^ in mineral soil and −0.03 to 0.42 kg C m^−2^ year^−1^ on peat soil, respectively. Meanwhile, NEE of primary forests in South America ranged from −1.19 to −0.09 kg C m^−2^ year^−1^ (mineral soil) (Fu et al. 2018; Table S1). Similarly, mean WUE at low LUI in SEA is slightly higher between 2.63 to 6.28 g C kg^−1^ H_2_O^−1^ compared to South America with values between 0.95 and 5.79 g C kg^−1^ H_2_O^−1^ (Table S2) (Costa et al. 2022). More data is needed to capture better representative values of carbon and water fluxes in each region, particularly given that in each region high LUI is related to different plantation types, such as SEA dominated by fast growing monoculture (e.g., rubber, oil palm and Acacia) (Deshmukh et al. 2023; Meijide et al. 2020; Wang et al. 2022), while South America tends to focus on combination of species such as Eucalyptus, coffee, *Gmelina arborea* with agroforestry system (Aguirre and Trilleras 2024; Chinchilla‐Soto et al. 2021; Maier et al. 2017).

### 4.4 Methodological Limitations

This study represents the first comprehensive analysis of the effects of LUI on carbon and water fluxes in SEA. However, our approach has some limitations. First, one of the issues is related to pre‐ and postprocessing of EC fluxes. In contrast to more centralized networks like NEON, ICOS, or Ameriflux, the sites we used are less harmonized. Although similar methods were used to calculate fluxes at most sites, differences in instrument characteristics (i.e., sensor sensitivity, measurement system, sampling frequency) can lead to uncertainties in the calculated fluxes (Kondo et al. 2017). Inconsistent techniques, such as different gap‐filling methods, increase uncertainties in annual carbon fluxes between sites (Vekuri et al. 2023). Despite significant efforts within the flux community to compare, harmonize, and validate various instrumental setups for multisite synthesis studies (Pastorello et al. 2020), progress in harmonizing fluxes in the SEA region remains very limited and requires further exploration.

Secondly, a low energy balance closure (EBC) is a long‐standing problem for EC measurements. Notably, many studies lack data on soil heat fluxes leading to systematic energy imbalance in EC measurements (Mauder et al. 2024). In our case, some of the sites with low EBC had also a shorter data coverage (e.g., BKS, MKL) (see Table S6). The underlying reasons for a low EBC is not necessarily due to instrument and data processing errors, but also includes the absence of additional energy terms (e.g., canopy heat storage, biomass energy storage) and secondary circulations (e.g., vertical‐horizontal advection) (Mauder et al. 2020). Furthermore, in tropical regions, high humidity can influence mass and energy exchanges between surface and atmosphere, resulting in high EBC variability (Jin et al. 2022). A study by Zhang et al. (2023) mentioned that the underestimation of the turbulent fluxes is most apparent when relative humidity exceeds 70%. In addition, forest ecosystems with tall canopies as typical for the tropics can develop relevant energy storage terms within the canopy and the understory, contributing to a lower EBC when not accounted for (Murkute et al. 2024). Considering such energy storage effects of forests could improve the EBC (Butterworth et al. 2024; Mauder et al. 2020).

Third, the measurement periods vary between sites. Considering that this region is strongly influenced by the El Nino Southern Oscillation (ENSO), the measurement period has been found to greatly impact the variability and uncertainties of carbon, energy, and water fluxes (Stiegler et al. 2019; Takamura et al. 2023). Although utilizing multiple years of measurements per site helped to reduce the effects of inter‐annual variability, we cannot fully rule out that some site differences are due to different measurement periods. Local climate conditions may also affect our results when calculating the impact of LUI, as shown by the two rubber plantations in this study. One rubber plantation is located in an area with lower rainfall (RFC) compared to another traditional growing area (RFN) where there is more rainfall (Wang et al. 2022). In addition, two available datasets for oil palm plantations are located in different soil types and at different ages, that is, mineral and mature (19 years old) (JOP) and peat soils and young age (3–7 years old) (SBW). Since all other sites were in the mature stage, we did not include the young oil palm plantation on peat soil in the aggregated analysis. In plantation systems, management practices have a major influence on environmental impacts and are tailored to site conditions. More intensive management, such as increased fertilization and herbicide use, is often necessary under less favorable conditions affecting the observed fluxes. Despite these limitations, this study provides the first synthesis of eddy covariance flux data from SEA that focuses on LUI gradients. It contributes to a deeper understanding of land‐atmosphere interactions at different LUI and provides valuable insights for sustainable land management in the region.

### 4.5 Future Implications

The SEA region is recognized not only as a biodiversity hotspot but also for experiencing rapid land use and land cover change (LULCC) over the past two decades (Estoque et al. 2019). Changes in vegetation structure over the rotation time of a plantation is a major driver in ecosystem functioning, making research on the complete rotation cycle essential. The necessity of monitoring the full rotation cycle is further emphasized by the fact that in the postlogging period plantations are typically a net carbon source to the atmosphere (Meijide et al. 2020; Mills et al. 2023). While long term monitoring using EC in SEA covering full rotation cycles in plantation systems is still limited, some sites will reach the end of the first rotation (e.g., JOP) thus providing new opportunities in the future. This knowledge gap could also be addressed by integrating data‐driven (or machine learning) approaches along with observation from FLUXNET sites, spatially continuous satellite (covering different plantation ages) and meteorological data to generate gridded carbon and water fluxes, similar to work of Zhang et al. (2020) in global semiarid regions. Research combining the high temporal resolution of EC systems with high spatial resolution from MODIS and Landsat has been expanding in recent years, as demonstrated by Walther et al. (2022). In addition, systematic assessments of upscaling carbon fluxes from EC monitoring, combined with atmospheric inversion and dynamic vegetation models (DGVMs) are also growing as done by Jung et al. (2020) and Grassi et al. (2023). Therefore, implementing a multiapproach strategy to understand the impact of LUI gradient throughout the full ecosystem cycle in tropical regions is a promising future direction.

## 5 Conclusions

This study shows that land use intensity (LUI) affects carbon and water fluxes in Southeast Asia, highlighting different trade‐offs and ecosystem vulnerabilities along the LUI gradient: (1) Mature high LUI on mineral soil, that is, as rubber and oil palm plantations, leads to higher carbon uptake at the expense of water availability, making these systems more susceptible to ecosystem water stress. (2) Medium LUI, that is, secondary forests, is most sensitive to environmental changes, which is reflected in a high correlation between carbon and water fluxes and meteorological parameters. This sensitivity is due to the transitional nature of these ecosystems as they recover from disturbance towards greater stability. (3) Low LUI, that is, primary forests, had the highest WUE. Although their carbon uptake was not as high as in the plantations, they are effective carbon sinks with high GPP and provide higher ecosystem resilience. Those results highlight a clear trade‐off in water and carbon fluxes along a LUI gradient. It can inform and support the development of land surface models for future predictions, particularly under the aspect of LUI and tropical plantation systems (Ali et al. 2022; Fan et al. 2015). With our analysis of SE Asian flux tower sites, which are partly not yet available in the large FLUXNET data compilations (Pastorello et al. 2020), we focus on a region that is currently rapidly changing due to land transformation and at the same time home of large, carbon‐rich tropical rainforests. As many nations base their emission reduction targets on agriculture, forestry and other land use (AFOLU), a more comprehensive understanding of the effects of LUI on carbon and water fluxes strengthens the strategic options for achieving the emission reduction targets based on the AFOLU sector.

## Author Contributions

**Bayu Budi Hanggara:** conceptualization, formal analysis, investigation, writing – original draft. **Christian Stiegler:** conceptualization, data curation, methodology. **Yoshiaki Hata:** data curation, validation. **Lulie Melling:** data curation, writing – review and editing. **Tania June:** validation. **Tomo'omi Kumagai:** validation. **Takashi Hirano:** data curation, validation. **Alexander Knohl:** conceptualization, funding acquisition, supervision, validation, writing – review and editing.

## Funding

This work was supported by The Deutsche Forschungsgemeinchaft (DFG) through ClimReg project (project number 532868192) and Collaborative Research Center 990 (project number: 192626868).

## Conflicts of Interest

The authors declare no conflicts of interest.

## Supporting information


**Data S1:** gcb70753‐sup‐0001‐Supinfo.docx.

## Data Availability

The data that support the findings of this study are openly available in Zenodo at https://doi.org/10.5281/zenodo.18236666. The primary data of carbon fluxes and meteorological datasets used in this study (LHP, MKL, PSO, SKR, BKS, DFR, PDF, and JOP) are publicly available at the Asiaflux database (https://db.cger.nies.go.jp/asiafluxdb/?page_id=16), and (RFC and RFN) can be found in an online repository from Wang et al. (2022) (https://zenodo.org/records/6474361). Other peat site data on PUF, PDF, and PDB can be found in online Supporting Information from Hirano et al. (2024) (https://doi.org/10.1038/s43247‐024‐01387‐7). Kampar peat site data (KIF, KDF, KAP) are publicly available at online Supporting Information from Deshmukh et al. (2023) (https://doi.org/10.1038/s41586‐023‐05860‐9) and Deshmukh et al. (2021) (https://doi.org/10.1038/s41561‐021‐00785‐2).
