# Supplementary material for: Trade‐Offs Between Carbon and Water Fluxes Along a Land Use Intensity Gradient in Southeast Asian Forests and Plantations
Source: Glob Chang Biol. 2026 Feb 24;32(2):e70753. doi: 10.1111/gcb.70753 (PMC12930338; doi:10.1111/gcb.70753)
Supplement: Supplementary file 1 — Data S1: gcb70753‐sup‐0001‐Supinfo.docx. [file GCB-32-e70753-s001.docx]

Supplementary information

***“Trade-off between carbon and water fluxes along a land use intensity gradient in Southeast Asian forests and plantations”***

Bayu Budi Hanggara* ^1,2)^ Christian Stiegler ^1,3)^ Yoshiaki Hata ^4)^ Lulie Melling ^5)^ Tania June ^6)^ Tomo’omi Kumagai ^4,7)^ Takashi Hirano^8)^ Alexander Knohl ^1)^

Table S1. Global comparison of carbon fluxes in tropical countries across continents

|  | **Country** | **LUI** | **Ecosystem types** | **Site** | **Soil type** | **Year** | **NEE** | **GPP** | **Reco** | **References** |
| --- | --- | --- | --- | --- | --- | --- | --- | --- | --- | --- |
|  |  |  |  |  |  |  | kg C m^-2^ yr^-1^ | | |  |
| South | Brazil | Low | Primary forest | Caxiuana-PA | Mineral soil | 2005 - 2008 | -0.60 ± 0.45 |  |  | (Alves et al., 2024) |
| America | Brazil | Low | Primary forest | BR-Ban | Mineral soil | 2003 - 2006 | -1.19 ± 0.17 | 2.62 ± 0.20 | 1.43 ± 0.37 | (Fu et al., 2018) |
|  | Brazil | Low | Primary forest | BR-Cax | Mineral soil | 1999 - 2003 | -1.02 ± 0.08 | 2.79 ± 0.36 | 1.78 ± 1.78 | (Fu et al., 2018) |
|  | Brazil | Low | Primary forest | BR-Ji2 | Mineral soil | 2000 - 2002 | -0.07 ± 0.12 | 2.99 ± 0.15 | 2.26 ± 2.25 | (Fu et al., 2018) |
|  | Brazil | Low | Primary forest | BR-Ma2 | Mineral soil | 1999 - 2006 | -0.09 ± 0.15 | 2.51 ± 0.60 | 1.58 ± 1.57 | (Fu et al., 2018) |
|  | Brazil | Low | Primary forest | BR-Sa1 | Mineral soil | 2002 - 2011 | -0.06 ± 0.19 | 3.63 ± 0.59 | 3.95 ± 0.65 | (Fu et al., 2018) |
|  | Brazil | Low | Primary forest | BR-Sa3 | Mineral soil | 2000 - 2004 | -0.17 ± 0.32 | 3.35 ± 0.24 | 3.48 ± 0.50 | (Fu et al., 2018) |
|  | French Guyana | Low | Primary forest | GF-Guy | Mineral soil | 2004 - 2014 | -0.15 ± 0.08 | 3.67 ± 0.22 | 3.42 ± 0.27 | (Fu et al., 2018) |
|  | Brazil | Low | Dry topical forest | Caatinga | Mineral soil | 2014 | -0.17 | 0.41 | 0.25 | (Mendes et al., 2020) |
|  | Brazil | Low | Dry topical forest | Caatinga | Mineral soil | 2015 | -0.15 | 0.33 | 0.19 | (Mendes et al., 2020) |
|  | Panama | Medium | Secondary forest | PA-SPn | Mineral soil | 2007 - 2009 | -0.06 ± 0.25 | 2.21 ± 0.02 | 1.67 ± 0.04 | (Mendes et al., 2020) |
|  | Panama | High | Monoculture | LS1 | Mineral soil | 2007 - 2008 | 0.23 | 0.27 |  | (Kunert et al., 2019) |
|  | Panama | High | Monoculture | AE2 | Mineral soil | 2007 - 2008 | 0.06 | 0.08 |  | (Kunert et al., 2019) |
|  | Panama | High | Monoculture | HC1 | Mineral soil | 2007 - 2008 | 0.10 | 0.13 |  | (Kunert et al., 2019) |
|  | Panama | High | Monoculture | CO2 | Mineral soil | 2007 - 2008 | 0.05 | 0.06 |  | (Kunert et al., 2019) |
|  | Panama | High | Monoculture | TR1 | Mineral soil | 2007 - 2008 | 0.08 | 0.12 |  | (Kunert et al., 2019) |
| Africa | Zambia | Low | Primary forest | ZM-Mon | Mineral soil | 2000 - 2009 | -0.15 | 1.56 |  | (Besnard et al., 2018) |
|  | Ghana | Low | Primary forest | GH-Ank | Mineral soil | 2011 - 2014 | -0.77 ± 0.23 | 2.77 ± 0.47 | 2.25 ± 0.28 | (Besnard et al., 2018) |
|  | Sudan | Medium | Degraded woodland | Nangatchori | Mineral soil | 2005 - 2007 | 0.03 ± 0.01 |  |  | (Besnard et al., 2018) |
| Asia - | Indonesia | Low | Intact forest | KIF | Peat soil | 2016 - 2022 | 0.42 ± 0.10 |  |  | (Deshmukh et al., 2023) |
| Pacific | Indonesia | Low | Peatswamp forest | PUF | Peat soil | 2005 - 2017 | -0.03 ± 0.36 | 3.78 ± 0.17 | 3.75 ± 0.26 | (Hirano et al., 2024) |
|  | Malaysia | Low | Primary forest | LHP | Mineral soil | 2010 - 2019 | -0.85 ± 0.16 | 3.39 ± 0.27 | 2.55 ± 0.18 | (Takamura et al., 2023) |
|  | Malaysia | Low | Primary forest | PSO | Mineral soil | 2003 - 2009 | -0.11 ± 0.05 | 3.25 ± 0.09 | 3.14 ± 0.06 | (Kosugi et al., 2012) |
|  | Thailand | Low | Primary forest | MKL | Mineral soil | 2003 - 2004 | -0.02 ± 0.20 | 3.24 ± 0.27 | 2.98 ± 0.37 | (Saigusa et al., 2013) |
|  | Thailand | Low | Primary forest | SKR | Mineral soil | 2001 - 2003 | -0.32 ± 0.24 | 2.72 ± 0.03 | 3.15 ± 0.19 | (Saigusa et al., 2013) |
|  | Indonesia | Medium | Degraded peatswamp | KDF | Peat soil | 2016 - 2022 | 1.11 ± 0.12 |  |  | (Deshmukh et al., 2023) |
|  | Indonesia | Medium | Drained peatswamp | PDF | Peat soil | 2002 - 2016 | 0.38 ± 0.29 | 3.21 ± 0.25 | 3.60 ± 0.30 | (Hirano et al., 2024) |
|  | Indonesia | Medium | Burned peatswamp | PDB | Peat soil | 2005 - 2016 | -0.18 ± 0.43 | 1.90 ± 0.25 | 1.72 ± 0.32 | (Hirano et al., 2024) |
|  | Indonesia | Medium | Secondary forest | BKS | Mineral soil | 2001 - 2002 | -0.7 | 2.27 | 1.95 | (Kato and Tang, 2008) |
|  | Thailand | Medium | Secondary forest | DFR | Mineral soil | 2015 - 2017 | -0.78 ± 0.25 | 3.04 ± 0.50 | 2.30 ± 0.35 | (Wang et al., 2022) |
|  | Indonesia | High | Oil palm | Pompa air | Mineral soil | 2013 - 014 | 1.01 ± 0.05 |  |  | (Meijide et al., 2020) |
|  | Indonesia | High | Oil palm | JOP | Mineral soil | 2014 - 2016 | -0.75 ± 0.04 |  |  | (Meijide et al., 2020) |
|  | Indonesia | High | Oil palm | Jambi | Peat soil | 2014 2016 | 0.33 ± 0.28^*^ |  |  | (Meijide et al., 2020) |
|  | Indonesia | High | Acacia | KAP | Peat soil | 2016 - 2021 | 0.26 ± 0.12 |  |  | (Deshmukh et al., 2023) |
|  | Malaysia | High | Oil palm | Sebungan | Peat soil | 2016 - 2019 | 1.08 ± 0.16 | 0.50 ± 0.22 | 1.58 ± 0.06 | (McCalmont et al., 2023) |
|  | Malaysia | High | Oil Palm | Sebungan | Peat soil | 2017 - 2019 | 0.01 ± 0.02 | 1.15 ± 0.07 | 1.29 ± 0.05 | (McCalmont et al., 2023) |
|  | Thailand | High | Rubber | RFC | Mineral soil | 2015 - 2018 | -1.23 ± 0.20 | 2.54 ± 0.18 | 1.35 ± 0.14 | (Wang et al., 2022) |
|  | Thailand | High | Rubber | RFN | Mineral soil | 2017 - 2018 | -1.11 ± 0.27 | 2.19 ± 0.24 | 1.04 ± 0.01 | (Wang et al., 2022) |

Table S2. Global comparison of water use efficiency in tropical countries across continents

|  | **Country** | **LUI** | **Ecosystem types** | **Site** | **Year** | **WUE** | **References** |
| --- | --- | --- | --- | --- | --- | --- | --- |
|  |  |  |  |  |  |  |  |
| South - America | Brazil | Low | Dry tropical forest | Caatinga | 2014 - 2015 | 5.79 | (Costa et al., 2022) |
|  | Brazil | Low | Primary forest | Amazonia | 2009 - 2011 | 1.82 | (Costa et al., 2022) |
|  | Brazil | Low | Floodplain forest | Cerrado | 2004 - 2006 | 1.61 | (Costa et al., 2022) |
|  | Brazil | Low | Wetland | Pantanal | 2015 - 2016 | 0.95 | (Costa et al., 2022) |
|  | French Guiana | Low | Tropical rainforest | Paracou | 2004 - 2014 | 2.65 | (Aguilos et al., 2018) |
|  | Costa Rica | High | Coffee plantation | Aajuela | 1999 - 2019 | 1 - 3 | (Chinchilla-Soto et al., 2021) |
|  | Columbia | High | *Gmelina arborea* | Caribbean | 2007 - 2014 | 0.93 | (Aguirre and Trilleras, 2024) |
|  | Columbia | High | *Gmelina arborea* | Inter-Andean valleys | 2007 - 2014 | 0.77 | (Aguirre and Trilleras, 2024) |
|  | Brazil | High | *Eucalyptus benthamii* | Ravenel | 2013 – 2014 | 2.86 | (Maier et al., 2017) |
|  | Brazil | High | Pine plantation | Ravenel | 2013 - 2014 | 1.72 | (Maier et al., 2017) |
| Asia - Pacific | Malaysia | Low | Primary forest | LHP | 2010 - 2019 | 6.28 ± 3.68 | (Takamura et al., 2023) |
|  | Malaysia | Low | Primary forest | PSO | 2003 -2009 | 2.63 ± 0.88 | (Kosugi et al., 2012) |
|  | Thailand | Low | Primary forest | MKL | 2003 -2004 | 6.07 ± 3.28 | (Saigusa et al., 2013) |
|  | Thailand | Low | Primary forest | SKR | 2001 - 2003 | 4.83 ± 2.37 | (Saigusa et al., 2013) |
|  | Indonesia | Low | Peatswamp forest | PUF | 2005 - 2017 | 2.87 ± 0.86 | (Hirano et al., 2024) |
|  | Indonesia | Medium | Secondary forest | BKS | 2001 | 4.36 ± 3.13 | (Kato and Tang, 2008) |
|  | Indonesia | Medium | Secondary forest | PDF | 2002 - 2016 | 2.81 ± 2.71 | (Hirano et al., 2024) |
|  | Indonesia | Medium | Burned peatswamp | PDB | 2005 - 2016 | 1.57 ± 0.52 | (Hirano et al., 2024) |
|  | Thailand | Medium | Secondary forest | DFR | 2015 - 2017 | 2.57 ± 2.14 | (Wang et al., 2022) |
|  | China | High | Rubber plantation | Xishuangbanna | 2013 – 2016 | 2.34 ± 0.37 | (Lin et al., 2018) |
|  | Indonesia | High | Oil palm | Pompa air | 2014 – 2015 | 3.29 ± 0.32 | (Meijide et al., 2017) |
|  | Indonesia | High | plantation | JOP | 2013 - 2014 | 2.76 ±0.40 | (Meijide et al., 2017) |
|  | Thailand | High | Rubber plantation | RFC | 2015 - 2018 | 2.46 ± 0.81 | (Wang et al., 2022) |
|  | Thailand | High | Rubber plantation | RFN | 2017 - 2018 | 2.09 ± 0.68 | (Wang et al., 2022) |

Table S3. List of data availability of each site. This overview is based on variable availability for daily values.

| LUI | Ecosystem | Site | Soil_type | year | NEE | GPP | Reco | LE | H | G | Rn | Rg | PAR | Tair | RH | VPD | Tsoil | precipt | SWC | ET | REW | WUE | iWUE | uWUE | LUE |
| --- | --- | --- | --- | --- | --- | --- | --- | --- | --- | --- | --- | --- | --- | --- | --- | --- | --- | --- | --- | --- | --- | --- | --- | --- | --- |
|  |  |  |  |  |  |  |  |  |  |  |  |  |  |  |  |  |  |  |  |  |  |  |  |  |  |
| Low | primary forest | LHP | Mineral | 2010-2019 | v | v | v | v | v |  | v | v | v | v | v | v |  | v | v | v | v | v | v | v | v |
| Low | primary forest | MKL | Mineral | 2003-2004 | v | v | v | v | v | v | v |  | v | v | v | v | v | v | v | v | v | v | v | v | v |
| Low | primary forest | PSO | Mineral | 2003-2009 | v | v | v | v | v | v | v | v | v | v |  | v | v | v | v | v | v | v | v | v | v |
| Low | primary forest | SKR | Mineral | 2001-2003 | v | v | v | v | v | v | v | v | v | v | v | v | v | v | v | v | v | v | v | v | v |
| Low | primary forest | KIF* | Peat | 2016-2022 | v |  |  |  |  |  |  |  | v | v |  | v |  |  |  |  |  |  |  |  |  |
| Low | primary forest | PUF* | Peat | 2005-2017 | v | v | v | v | v |  | v |  | v | v | v | v |  | v |  | v |  | v | v | v | v |
| Medium | secondary forest | KDF* | Peat | 2016-2022 | v |  |  |  |  |  |  |  | v | v |  | v |  |  |  |  |  |  |  |  |  |
| Medium | Secondary forest | DFR | Mineral | 2015-2017 | v | v | v | v | v |  | v | v | v | v | v | v |  | v | v | v | v | v | v | v | v |
| Medium | secondary forest | BKS | Mineral | 2001-2002 | v | v | v | v | v | V | v | v | v | v | v | v | v | v | v | v | v | v | v | v | v |
| Medium | secondary forest | PDF* | Peat | 2002-2016 | v | v | v | v | v |  | v |  | v | v | v | v |  | v | v | v | v | v | v | V | v |
| Medium | secondary forest | PDB* | Peat | 2005-2016 | v | v | v | v | v |  | v |  | v | v | v | v |  | v |  | v |  | v | v | v | v |
| High | oil palm | JOP | Mineral | 2014-2020 | v | v | v | v | v | v | v | v | v | v | v | v | v | v | v | v | v | v | v | v | v |
| High | oil palm | SBW* | Peat | 2011-2014 | v | v | v |  |  |  |  |  | v | v | v | v | v | v | v |  | v |  |  |  |  |
| High | acacia | KAP* | Peat | 2016-2022 | v |  |  |  |  |  |  |  |  |  |  |  |  |  |  |  |  |  |  |  |  |
| High | rubber | RFC | Mineral | 2015-2018 | v | v | v | v | v |  |  | v | v | v | v | v |  | v | v | v | v | v | v | v | v |
| High | rubber | RFN | Mineral | 2017-2018 | v | v | v | v | v |  |  | v | v | v | v | v |  | v | v | v | v | v | v | v | v |

Carbon fluxes: NEE (net ecosystem exchange), GPP (gross primary productivity), Reco (ecosystem respiration)
Energy fluxes: LE (latent heat), H (sensible heat), G (soil heat flux), LUE (light use efficiency)
Meteorological variable: Rn (net radiation), Rg (global radiation), PAR (photosynthetic active radiation), Tair (air temperature), RH (relative humidity), VPD (vapor pressure deficit), Tsoil (soil temperature), precipt (precipitation), SWC (soil water content), ET (evapotranspiration), REW (relative extractable water)
Water fluxes: WUE (water use efficiency), iWUE (inherent water use efficiency), uWUE (underly water use efficiency)

Table S4. Detailed carbon fluxes partitioning, gap-filled methods, and meteorological validations.

| **LUI** | **Ecosystem** | **Site** | **Soil_type** | **Fluxes partitioning** | **Gap-filled method** | **Meteorological gap-filled and quality control** |
| --- | --- | --- | --- | --- | --- | --- |
| Low | primary forest | LHP | Mineral | Temperature-respiration model followed Reichstein et al. (2005) | Marginal distribution sampling (MDS)  (Reichstein et al., 2005) | Linear regression wit an observation at a tower located 100 m away from the site |
| Low | primary forest | MKL | Mineral | Temperature-respiration model followed Reichstein et al. (2005) | Marginal distribution sampling (MDS)  (Reichstein et al., 2005) |  |
| Low | primary forest | PSO | Mineral | Nighttime partitioning combined optimum relationship with volumetric soil water content | Mean diurnal course combined optimum relationship with volumetric soil water content | Interpolation with an observation weather station at 430 m away from the site |
| Low | primary forest | SKR | Mineral | Temperature-respiration model followed Reichstein et al. (2005) | Marginal distribution sampling (MDS)  (Reichstein et al., 2005) |  |
| Low | primary forest | KIF* | Peat | Temperature-respiration model followed Reichstein et al. (2005) | Marginal distribution sampling (MDS) (Reichstein et al., 2005), artificial neural network (ANN) (Papale and Valentini, 2003), and random forest (RF) (Xu et al., 2018) |  |
| Low | primary forest | PUF* | Peat | Daytime Reco was derived using look-up tables for nighttime NEE. And GPP = daytime Reco - NEE | Look-up table under similar groundwater level and Marginal distributional sampling (MDS) by Reichstein et al. 2005 | Linear regression |
| Medium | secondary forest | KDF* | Peat | Temperature-respiration model followed Reichstein et al. (2005) | Marginal distribution sampling (MDS) (Reichstein et al., 2005), artificial neural network (ANN) (Papale and Valentini, 2003), and random forest (RF) (Xu et al., 2018) |  |
| Medium | Secondary forest | DFR | Mineral | Average of nighttime and daytime partitioning | Look-up tables under similar meteorological conditions and mean diurnal course by Reichstein et al. (2005) | Interpolation with three weather stations located close to the flux towers |
| Medium | secondary forest | BKS | Mineral | Temperature-respiration model followed Reichstein et al. (2005) | Marginal distribution sampling (MDS)  (Reichstein et al., 2005) |  |
| Medium | secondary forest | PDF* | Peat | Daytime Reco was derived using look-up tables for nighttime NEE. And GPP = daytime Reco - NEE | Look-up table under similar groundwater level and Marginal distributional sampling (MDS) by Reichstein et al. (2005) | Linear regression |
| Medium | secondary forest | PDB* | Peat | Daytime Reco was derived using look-up tables for nighttime NEE. And GPP = daytime Reco - NEE | Look-up table under similar groundwater level and Marginal distributional sampling (MDS) by Reichstein et al. (2005) | Linear regression |
| High | oil palm | JOP | Mineral | Temperature-respiration model followed Reichstein et al. 2005 | Marginal distributional sampling (MDS) by Reichstein et al. (2005) | Interpolation with two weather stations, 33 km and 47 km away from the site |
| High | oil palm | SBW* | Peat | Daytime Reco was ‘looked up’ from nighttime NEE using the same algorithm as for gap filling (MDS), and then GPP was calculated as the difference between NEE and daytime Reco (GPP = daytime Reco – NEE). During the nighttime, GPP was set as zero. | Marginal distributional sampling (MDS) by Reichstein et al. (2005) | Interpolation with a meteorological station 7.4 km away from the site. |
| High | acacia | KAP* | Peat | Temperature-respiration model followed Reichstein et al. (2005) | Marginal distribution sampling (MDS) (Reichstein et al., 2005), artificial neural network (ANN) (Papale and Valentini, 2003), and random forest (RF) (Xu et al., 2018) |  |
| High | rubber | RFC | Mineral | Average of nighttime and daytime partitioning | Look-up tables under similar meteorological conditions and mean diurnal course by Reichstein et al. (2005) | Interpolation with three weather stations located close to the flux towers |
| High | rubber | RFN | Mineral | Average of nighttime and daytime partitioning | Look-up tables under similar meteorological conditions and mean diurnal course by Reichstein et al. (2005) | Interpolation with three weather stations located close to the flux towers |

*An empty input means there is no explicit explanation in the literature

Table S5. Percentage NEE gap-filled, root mean square errors (RMSE) and flux processing package for each site

| **LUI** | **Ecosystem** | **Site** | **Soil_type** | **Year** | **Percentage gap-filled** | **RMSE (µ mol m^-2^ s^-1^)** | **Flux processing package** |
| --- | --- | --- | --- | --- | --- | --- | --- |
| Low | primary forest | LHP | Mineral | 2010 | 88.46 | 8.08 | ONEflux processing pipeline (Pastorello et al., 2020) combined with Rflux R package (Vitale et al., 2020): EddyPro (LI-COR, Lincoln, USA) |
|  |  |  |  | 2011 | 92.36 | 9.37 |  |
|  |  |  |  | 2012 | 80.23 | 8.83 |  |
|  |  |  |  | 2013 | 81.09 | 8.92 |  |
|  |  |  |  | 2014 | 80.56 | 7.38 |  |
|  |  |  |  | 2015 | 82.51 | 6.25 |  |
|  |  |  |  | 2016 | 80.52 | 6.30 |  |
|  |  |  |  | 2017 | 93.25 | 6.03 |  |
|  |  |  |  | 2018 | 90.95 | 8.61 |  |
|  |  |  |  | 2019 | 93.18 | 7.07 |  |
| Low | primary forest | MKL | Mineral | 2003 | 73.08 | 8.51 | ReddyPrco R package (Wutzler et al., 2018) |
|  |  |  |  | 2004 | 74.89 | 34.44 |  |
| Low | primary forest | PSO | Mineral | 2003 | 35.06 | 5.47 | (Kosugi et al., 2008; Kosugi et al., 2012) |
|  |  |  |  | 2004 | 71.74 | 4.54 |  |
|  |  |  |  | 2005 | 58.20 | 4.51 |  |
|  |  |  |  | 2006 | 55.79 | 5.07 |  |
|  |  |  |  | 2007 | 30.48 | 6.05 |  |
|  |  |  |  | 2008 | 29.67 | 6.21 |  |
|  |  |  |  | 2009 | 29.92 | 6.53 |  |
| Low | primary forest | SKR | Mineral | 2001 | 36.95 | 2.12 | ReddyPrco R package (Wutzler et al., 2018) |
|  |  |  |  | 2002 | 17.88 | 2.31 |  |
|  |  |  |  | 2003 | 31.79 | 2.52 |  |

Table S5 (cont). Percentage NEE gap-filled, root mean square errors (RMSE) and flux processing package for each site

| **LUI** | **Ecosystem** | **Site** | **Soil_type** | **Year** | **Percentage gap-filled** | **RMSE** | **Flux processing package** |
| --- | --- | --- | --- | --- | --- | --- | --- |
| Low | primary forest | KIF* | Peat | 2017 |  |  | ReddyPrco R package (Wutzler et al., 2018) |
|  |  |  |  | 2018 |  |  |  |
|  |  |  |  | 2019 |  |  |  |
|  |  |  |  | 2020 |  |  |  |
|  |  |  |  | 2021 |  |  |  |
| Low | primary forest | PUF* | Peat | 2005 |  |  | Flux Calculator software (Ueyama et al., 2012) |
|  |  |  |  | 2006 |  |  |  |
|  |  |  |  | 2007 |  |  |  |
|  |  |  |  | 2008 |  |  |  |
|  |  |  |  | 2009 |  |  |  |
|  |  |  |  | 2010 |  |  |  |
|  |  |  |  | 2011 |  |  |  |
|  |  |  |  | 2012 |  |  |  |
|  |  |  |  | 2013 |  |  |  |
|  |  |  |  | 2014 |  |  |  |
|  |  |  |  | 2015 |  |  |  |
|  |  |  |  | 2016 |  |  |  |
|  |  |  |  | 2017 |  |  |  |
| Medium | secondary forest | KDF* | Peat | 2016 |  |  | ReddyPrco R package (Wutzler et al., 2018) |
|  |  |  |  | 2017 |  |  |  |
|  |  |  |  | 2018 |  |  |  |
|  |  |  |  | 2019 |  |  |  |
|  |  |  |  | 2020 |  |  |  |
|  |  |  |  | 2021 |  |  |  |
| Medium | Secondary forest | DFR | Mineral | 2015 |  |  | ReddyPrco R package (Wutzler et al., 2018) |
|  |  |  |  | 2016 |  |  |  |
|  |  |  |  | 2017 |  |  |  |
|  |  |  |  |  |  |  |  |

Table S5 (cont). Percentage NEE gap-filled, root mean square errors (RMSE) and flux processing package for each site

| **LUI** | **Ecosystem** | **Site** | **Soil_type** | **Year** | **Percentage gap-filled** | **RMSE** | **Flux processing package** |
| --- | --- | --- | --- | --- | --- | --- | --- |
| Medium | secondary forest | BKS | Mineral | 2001 |  |  | ReddyPrco R package (Wutzler et al., 2018) |
|  |  |  |  | 2002 | 68.63 | 73.48 |  |
| Medium | secondary forest | PDF* | Peat | 2002 | 71.60 |  | Flux Calculator software (Ueyama et al., 2012) |
|  |  |  |  | 2003 | 79.37 |  |  |
|  |  |  |  | 2004 | 71.07 |  |  |
|  |  |  |  | 2005 | 68.43 |  |  |
|  |  |  |  | 2006 | 78.18 |  |  |
|  |  |  |  | 2007 | 89.03 |  |  |
|  |  |  |  | 2008 | 93.60 |  |  |
|  |  |  |  | 2009 | 96.14 |  |  |
|  |  |  |  | 2010 | 71.79 |  |  |
|  |  |  |  | 2011 | 79.14 |  |  |
|  |  |  |  | 2012 | 84.44 |  |  |
|  |  |  |  | 2013 | 84.32 |  |  |
|  |  |  |  | 2014 | 78.36 |  |  |
|  |  |  |  | 2015 | 79.98 |  |  |
|  |  |  |  | 2016 | 74.23 |  |  |
| Medium | secondary forest | PDB* | Peat | 2005 | 38.34 |  | Flux Calculator software (Ueyama et al., 2012) |
|  |  |  |  | 2006 | 63.26 |  |  |
|  |  |  |  | 2007 | 66.54 |  |  |
|  |  |  |  | 2008 | 65.98 |  |  |
|  |  |  |  | 2009 | 68.09 |  |  |
|  |  |  |  | 2010 | 34.42 |  |  |
|  |  |  |  | 2011 | 48.34 |  |  |
|  |  |  |  | 2012 | 38.29 |  |  |
|  |  |  |  | 2013 | 37.59 |  |  |
|  |  |  |  | 2014 | 45.61 |  |  |

Table S5 (cont). Percentage NEE gap-filled, root mean square errors (RMSE) and flux processing package for each site

| **LUI** | **Ecosystem** | **Site** | **Soil_type** | **Year** | **Percentage gap-filled** | **RMSE** | **Flux processing package** |
| --- | --- | --- | --- | --- | --- | --- | --- |
| Medium | secondary forest | PDB* | Peat | 2015 | 57.21 |  |  |
|  |  |  |  | 2016 | 66.11 |  |  |
| High | oil palm | JOP | Mineral | 2014 | 40.94 | 4.78 | ReddyPrco R package (Wutzler et al., 2018) |
|  |  |  |  | 2015 |  |  |  |
|  |  |  |  | 2017 | 52.33 | 5.06 |  |
|  |  |  |  | 2018 | 17.02 | 5.81 |  |
|  |  |  |  | 2019 |  |  |  |
|  |  |  |  | 2020 | 41.96 | 7.91 |  |
| High | oil palm | SBW* | Peat | 2011 | 58.31 |  | Flux Calculator software (Ueyama et al., 2012) |
|  |  |  |  | 2012 | 74.20 |  |  |
|  |  |  |  | 2013 | 76.18 |  |  |
|  |  |  |  | 2014 | 72.48 |  |  |
| High | acacia | KAP* | Peat | 2016 |  |  | ReddyPrco R package (Wutzler et al., 2018) |
|  |  |  |  | 2017 |  |  |  |
|  |  |  |  | 2018 |  |  |  |
|  |  |  |  | 2019 |  |  |  |
|  |  |  |  | 2020 |  |  |  |
|  |  |  |  | 2021 |  |  |  |
| High | rubber | RFC | Mineral | 2015 |  |  | ReddyPrco R package (Wutzler et al., 2018) |
|  |  |  |  | 2016 |  |  |  |
|  |  |  |  | 2017 |  |  |  |
|  |  |  |  | 2018 |  |  |  |
| High | rubber | RFN | Mineral | 2017 |  |  | ReddyPrco R package (Wutzler et al., 2018) |
|  |  |  |  | 2018 |  |  |  |

- Due to limited access to original half-hourly data, some sites were unable estimate the percentage of gap-filled and their RMSE.

Table S6. Year-to-year carbon, energy, and water fluxes of every site. Mean ± stdev for C stocks (NEE, GPP, and Reco) were calculated based on yearly data, while the rest (Rn, LE, H, LUE, WUE, iWUE) were based on daily values.

| ***LUI*** | ***Site*** | ***Ecosystem type*** | ***Year*** | ***NEE*** | ***GPP*** | ***Reco*** | ***Gap-***  ***filled*** | ***Net radiation (Rn)*** | ***Latent heat (LE)*** | ***Sensible heat (H)*** | ***LUE*** | ***WUE*** | ***iWUE*** |
| --- | --- | --- | --- | --- | --- | --- | --- | --- | --- | --- | --- | --- | --- |
|  |  |  |  | (kg C | m^-2^ | yr^-1^) |  | (Wm^-2^) | (Wm^-2^) | (Wm^-2^) | (µmol CO_2_ µmol photon^-1^) | (g C kg^-1^ H_2_O^-1^) | (g C hPa kg^-1^ H2O^-1^) |
| Low (1) | LHP | Forest | 2010 | -1.03 | 3.63 | 2.59 | 88.46 | 127.59 ± 36.88 | 112.92 ± 53.04 | 38.84 ± 76.09 | 0.038 ± 0.013 | 7.73 ± 5.11 | 50.47 ± 34.28 |
|  |  |  | 2011 | -0.86 | 3.39 | 2.63 | 92.36 | 115.54 ± 38.69 | 139.04 ± 55.29 | 29.99 ± 20.43 | 0.036 ± 0.010 | 6.43 ± 2.83 | 37.70 ± 18.53 |
|  |  |  | 2012 | -1.08 | 3.65 | 2.66 | 80.23 | 125.28 ± 37.12 | 151.38 ± 53.30 | 38.13 ± 20.78 | 0.036 ± 0.008 | 6.48 ± 3.50 | 40.00 ± 19.22 |
|  |  |  | 2013 | -0.90 | 3.49 | 2.58 | 81.09 | 184.13 ± 43.60 | 163.10 ± 54.32 | 42.13 ± 25.51 | 0.033 ± 0.007 | 5.89 ± 4.11 | 35.36 ± 18.71 |
|  |  |  | 2014 | -0.70 | 3.43 | 2.70 | 80.56 | 182.34 ± 47.93 | 140.77 ± 50.50 | 39.90 ± 24.34 | 0.035 ± 0.011 | 6.44 ± 3.07 | 38.24 ± 15.72 |
|  |  |  | 2015 | -0.78 | 3.30 | 2.54 | 82.51 | 187.77 ± 44.22 | 148.44 ± 48.53 | 37.38 ± 22.53 | 0.032 ± 0.010 | 5.98 ± 3.10 | 39.49 ± 19.13 |
|  |  |  | 2016 | -0.88 | 3.69 | 2.75 | 80.52 | 188.86 ± 42.56 | 157.26 ± 58.23 | 41.12 ± 24.93 | 0.035 ± 0.011 | 6.46 ± 4.46 | 42.86 ± 28.12 |
|  |  |  | 2017 | -0.90 | 3.48 | 2.60 | 93.25 | 183.57 ± 43.59 | 165.42 ± 53.79 | 47.82 ± 28.87 | 0.031 ± 0.007 | 5.92 ± 2.95 | 36.00 ± 15.53 |
|  |  |  | 2018 | -0.52 | 2.85 | 2.33 | 90.95 | 185.76 ± 43.22 | 151.36 ± 45.13 | 43.89 ± 22.21 | 0.029 ± 0.008 | 5.43 ± 2.13 | 38.10 ± 13.54 |
|  |  |  | 2019 | -0.90 | 3.03 | 2.14 | 93.18 | 187.48 ± 42.94 | 168.30 ± 76.41 | 51.99 ± 36.50 | 0.032 ± 0.013 | 6.17 ± 3.65 | 45.94 ± 32.58 |
|  |  | Mean ± stdev | | *-0.85 ± 0.16* | *3.39 ± 0.27* | *2.55 ± 0.18* |  | *166.83 ± 51.18* | *150.79 ± 55.25* | *40.81 ± 25.27* | *0.034 ± 0.010* | *6.28 ± 3.68* | *39.72 ± 21.78* |
|  |  |  | |  |  |  |  |  |  |  |  |  |  |
|  | MKL | Forest | 2003 | -0.16 | 3.49 | 3.24 | 73.08 | 138.15 ± 36.49 | 45.64 ± 14.08 | 18.56 ± 10.44 | 0.032 ± 0.016 | 6.39 ± 3.29 | 43.49 ± 9.82 |
|  |  |  | 2004 | 0.12 | 2.98 | 2.72 | 74.89 | 132.36 ± 35.00 | 39.67 ± 11.89 | 19.55 ± 13.76 | 0.028 ± 0.018 | 5.74 ± 3.25 | 49.11 ± 15.26 |
|  |  |  |  | *-0.02 ± 0.20* | *3.24 ± 0.36* | *2.98 ± 0.37* |  | *135.25 ± 35.85* | *42.65 ± 13.36* | *19.05 ± 12.22* | *0.030 ± 0.017* | *6.07 ± 3.28* | *46.30 ± 13.13* |
|  |  |  |  |  |  |  |  |  |  |  |  |  |  |
|  | PSO | Forest | 2003 | -0.05 | 3.16 | 3.11 | 35.06 | 148.99 ± 39.21 | 100.87 ± 20.63 | 47.50 ± 21.79 | 0.025 ± 0.017 | 2.35 ± 0.71 | 13.45 ± 4.05 |
|  |  |  | 2004 | -0.12 | 3.23 | 3.11 | 71.74 | 141.59 ± 35.05 | 100.90 ± 19.14 | 40.48 ± 18.30 | 0.025 ± 0.015 | 2.49 ± 0.73 | 15.26 ± 4.68 |
|  |  |  | 2005 | -0.14 | 3.22 | 3.09 | 58.20 | 151.23 ± 36.53 | 103.69 ± 19.84 | 47.26 ± 22.58 | 0.024 ± 0.018 | 2.56 ± 1.14 | 17.27 ± 5.73 |
|  |  |  | 2006 | -0.05 | 3.20 | 3.14 | 55.79 | 139.70 ± 35.53 | 99.29 ± 19.77 | 40.11 ± 19.38 | 0.025 ± 0.013 | 2.58 ± 0.57 | 15.08 ± 5.35 |
|  |  |  | 2007 | -0.21 | 3.39 | 3.18 | 30.48 | 135.23 ± 36.88 | 95.76 ± 21.86 | 39.56 ± 18.54 | 0.031 ± 0.033 | 2.92 ± 1.03 | 15.41 ± 6.87 |
|  |  |  | 2008 | -0.12 | 3.38 | 3.25 | 29.67 | 144.79 ± 36.73 | 101.95 ± 21.17 | 42.85 ± 21.16 | 0.026 ± 0.014 | 2.69 ± 0.75 | 14.15 ± 4.17 |
|  |  |  | 2009 | -0.10 | 3.21 | 3.10 | 29.92 | 147.76 ± 36.64 | 92.26 ± 19.10 | 55.18 ± 23.03 | 0.024 ± 0.017 | 2.84 ± 0.97 | 17.68 ± 6.24 |
|  |  |  |  | *-0.11 ± 0.05* | *3.25 ± 0.09* | *3.14 ± 0.06* |  | *144.18 ± 37.00* | *99.25 ± 20.54* | *44.70 ± 21.39* | *0.025 ± 0.019* | *2.63 ± 0.88* | *15.47 ± 5.57* |
|  |  |  |  |  |  |  |  |  |  |  |  |  |  |
|  | SKR | Forest | 2001 | -0.13 | 2.75 | 3.07 | 36.95 | 123.03 ± 41.55 | 43.41 ± 17.51 | 28.44 ± 14.66 | 0.021 ± 0.008 | 6.08 ± 3.08 | 39.62 ±17.77 |
|  |  |  | 2002 | -0.23 | 2.70 | 3.02 | 17.88 | 122.42 ± 37.45 | 56.40 ± 20.36 | 53.69 ± 25.85 | 0.021 ± 0.008 | 4.58 ± 2.05 | 35.70 ± 8.99 |
|  |  |  | 2003 | -0.59 | 2.70 | 3.37 | 31.79 | 112.97 ± 35.51 | 66.01 ± 21.39 | 58.00 ± 17.57 | 0.022 ± 0.008 | 4.25 ± 1.78 | 35.52 ± 8.59 |
|  |  |  |  | *-0.32 ± 0.24* | *2.72 ± 0.03* | *3.15 ± 0.19* |  | *125.60 ± 38.54* | *56.88 ± 21.84* | *49.13 ± 23.69* | *0.021 ± 0.008* | *4.83 ± 2.37* | *36.60 ± 11.78* |

Table S6 (cont). Year-to-year carbon, energy, and water fluxes of every site. Mean ± stdev for C stocks (NEE, GPP, and Reco) were calculated based on yearly data, while the rest (Rn, LE, H, LUE, WUE, iWUE) were based on daily values.

| **LUI** | **Site** | **Ecosystem type** | **Year** | **NEE** | **GPP** | **Reco** | **Gap-filled** | **Net radiation (Rn)** | **Latent heat (LE)** | **Sensible heat (H)** | **LUE** | **WUE** | **iWUE** |
| --- | --- | --- | --- | --- | --- | --- | --- | --- | --- | --- | --- | --- | --- |
|  | KIF | Forest | 2017 | 0.32 |  |  |  |  |  |  |  |  |  |
|  |  |  | 2018 | 0.25 |  |  |  |  |  |  |  |  |  |
|  |  |  | 2019 | 0.70 |  |  |  |  |  |  |  |  |  |
|  |  |  | 2020 | 0.43 |  |  |  |  |  |  |  |  |  |
|  |  |  | 2021 | 0.38 |  |  |  |  |  |  |  |  |  |
|  |  |  |  | *0.42* *± 0.10* |  |  |  |  |  |  |  |  |  |
|  |  |  |  |  |  |  |  |  |  |  |  |  |  |
|  | PUF | Forest | 2005 | 0.01 | 3.71 | 3.71 | 80.06 | 152.56 ± 33.81 | 107.79 ± 28.65 | 33.53 ± 14.16 | 0.029 ± 0.006 | 2.85 ± 0.79 | 19.45 ± 3.78 |
|  |  |  | 2006 | 0.35 | 3.65 | 4.01 | 73.89 | 140.49 ± 40.45 | 117.48 ± 36.43 | 52.73 ± 21.50 | 0.028 ± 0.006 | 2.60 ± 0.80 | 19.00 ± 5.11 |
|  |  |  | 2007 | -0.25 | 3.98 | 3.74 | 70.41 | 157.07 ± 34.02 | 120.86 ± 32.59 | 67.72 ± 16.84 | 0.030 ± 0.006 | 2.69 ± 0.62 | 19.68 ± 4.95 |
|  |  |  | 2008 | -0.13 | 4.04 | 3.90 | 75.14 | 151.09 ± 34.21 | 110.59 ± 29.20 | 35.60 ± 22.01 | 0.026± 0.005 | 2.97 ± 0.64 | 20.59 ± 4.44 |
|  |  |  | 2009 | 0.36 | 3.71 | 4.06 | 73.64 | 147.47 ± 35.73 | 105.59 ± 27.01 | 25.71 ± 10.31 | 0.028 ± 0.006 | 2.94 ± 1.21 | 21.81 ± 5.35 |
|  |  |  | 2010 | -0.22 | 3.68 | 3.46 | 69.96 | 155.10 ± 39.58 | 107.15 ± 27.12 | 22.85 ± 9.60 | 0.029 ± 0.007 | 2.81 ± 0.68 | 14.77 ± 4.31 |
|  |  |  | 2011 | -0.24 | 3.82 | 3.57 | 84.10 | 154.05 ± 35.37 | 109.97 ± 27.86 | 25.01 ± 9.53 | 0.028 ± 0.004 | 2.84 ± 0.94 | 17.36 ± 4.40 |
|  |  |  | 2012 | -0.46 | 4.04 | 3.57 | 79.42 | 152.22 ± 36.14 | 109.04 ± 28.69 | 25.16 ± 8.61 | 0.030 ± 0.005 | 3.04 ± 0.75 | 18.54 ± 5.08 |
|  |  |  | 2013 | -0.49 | 3.95 | 3.46 | 67.93 | 155.69 ± 35.75 | 112.54 ± 29.42 | 25.07 ± 10.20 | 0.029 ± 0.006 | 2.88 ± 0.78 | 18.30 ± 4.63 |
|  |  |  | 2014 | -0.07 | 3.79 | 3.71 | 85.06 | 147.93 ± 37.54 | 106.98 ± 30.04 | 27.29 ± 11.50 | 0.028 ± 0.005 | 2.93 ± 0.89 | 18.29 ± 5.46 |
|  |  |  | 2015 | 0.79 | 3.49 | 4.28 | 73.56 | 137.66 ± 46.84 | 95.48 ± 33.52 | 30.55 ± 14.75 | 0.030 ± 0.008 | 3.10 ± 0.89 | 20.86 ± 7.49 |
|  |  |  | 2016 | -0.15 | 3.60 | 3.44 | 65.28 | 149.64 ± 39.93 | 101.62 ± 31.93 | 40.72 ± 19.40 | 0.028 ± 0.007 | 2.97 ± 0.87 | 17.79 ± 6.99 |
|  |  |  | 2017 | 0.14 | 3.68 | 3.85 | 75.42 | 144.48 ± 38.03 | 117.90 ± 32.59 | 16.84 ± 10.04 | 0.025 ± 0.005 | 2.62 ± 1.50 | 14.51 ± 4.52 |
|  |  |  |  | *-0.03 ± 0.36* | *3.78 ± 0.17* | *3.75 ± 0.26* |  | *149.53 ± 37.55* | *109.57 ± 33.02* | *31.73 ± 18.75* | *0.028 ± 0.006* | *2.87 ± 0.86* | *18.65 ± 5.67* |
|  |  |  |  |  |  |  |  |  |  |  |  |  |  |
| Medium | BKS | Secondary | 2001 | -0.46* |  |  |  | 119.23 ± 108.05 | 26.66 ± 34.03 | 24.83 ± 27.88 |  |  |  |
| (2) |  | forest | 2002 | -0.70 | 2.27 | 1.95 | 68.63 | 175.30 ± 70.07 | 32.41 ± 18.97 | 25.71 ± 22.65 | 0.011 ± 0.007 | 4.36 ± 3.13 | 26.57 ± 23.58 |
|  |  |  |  |  |  |  |  | *146.72 ± 95.57* | *30.09 ± 26.24* | *25.35 ± 24.92* |  |  |  |
|  |  |  |  |  |  |  |  |  |  |  |  |  |  |
|  | DFR | Secondary | 2015 | -0.54 | 2.86 | 2.45 |  | 211.58 ± 39.18 | 101.39 ± 35.58 | 31.38 ± 29.31 | 0.024 ± 0.013 | 2.42 ± 0.87 | 25.16 ± 13.59 |
|  |  |  | 2016 | -0.77 | 2.66 | 1.90 |  | 205.59 ± 71.82 | 88.67 ± 51.91 | 39.75 ± 40.53 | 0.024 ± 0.016 | 2.81 ± 3.56 | 23.70 ± 13.87 |
|  |  |  | 2017 | -1.03 | 3.60 | 2.55 |  | 208.88 ± 71.65 | 117.71 ± 41.34 | 41.34 ± 12.84 | 0.030 ± 0.015 | 2.50 ± 0.61 | 20.15 ± 14.44 |
|  |  |  |  | *-0.78 ± 0.25* | *3.04 ± 0.50* | *2.30 ± 0.35* |  | *208.08 ± 61.20* | *102.58 ± 45.04* | *28.00 ± 35.87* | *0.026 ± 0.015* | *2.57 ± 2.14* | *23.16 ± 14.14* |
|  |  |  |  |  |  |  |  |  |  |  |  |  |  |

Table S6 (cont). Year-to-year carbon, energy, and water fluxes of every site. Mean ± stdev for C stocks (NEE, GPP, and Reco) were calculated based on yearly data, while the rest (Rn, LE, H, LUE, WUE, iWUE) were based on daily values.

| **LUI** | **Site** | **Ecosystem type** | **Year** | **NEE** | **GPP** | **Reco** | **Gap-filled** | **Net radiation (Rn)** | **Latent heat (LE)** | **Sensible heat (H)** | **LUE** | **WUE** | **iWUE** |
| --- | --- | --- | --- | --- | --- | --- | --- | --- | --- | --- | --- | --- | --- |
|  | KDF | Secondary | 2016 | 0.99 |  |  |  |  |  |  |  |  |  |
|  |  | Degraded | 2017 | 1.06 |  |  |  |  |  |  |  |  |  |
|  |  | forest | 2018 | 1.12 |  |  |  |  |  |  |  |  |  |
|  |  |  | 2019 | 1.18 |  |  |  |  |  |  |  |  |  |
|  |  |  | 2020 | 1.09 |  |  |  |  |  |  |  |  |  |
|  |  |  | 2021 | 1.33 |  |  |  |  |  |  |  |  |  |
|  |  |  |  | *1.11 ± 0.12* |  |  |  |  |  |  |  |  |  |
|  |  |  |  |  |  |  |  |  |  |  |  |  |  |
|  | PDF | Secondary | 2002 | 0.58 | 3.14 | 3.74 | 71.60 | 126.50 ± 51.88 | 78.18 ± 34.23 | 15.04 ± 13.74 | 0.024 ± 0.005 | 2.56 ± 0.62 | 19.27 ± 6.84 |
|  |  | Forest | 2003 | 0.52 | 3.35 | 3.87 | 79.37 | 139.31 ± 51.00 | 75.90 ± 29.31 | 13.22 ± 13.47 | 0.024 ± 0.004 | 2.90 ± 0.70 | 23.04 ± 7.02 |
|  |  |  | 2004 | 0.46 | 3.46 | 3.93 | 71.07 | 135.59 ± 46.05 | 77.23 ± 31.08 | 17.38 ± 12.88 | 0.026 ± 0.004 | 2.91 ± 0.66 | 21.55 ± 6.62 |
|  |  |  | 2005 | 0.53 | 3.38 | 3.93 | 68.43 | 139.80 ± 48.86 | 77.29 ± 31.38 | 18.76 ± 12.42 | 0.025 ± 0.005 | 2.85 ± 0.65 | 20.13 ± 5.92 |
|  |  |  | 2006 | 0.50 | 3.52 | 4.01 | 78.18 | 125.98 ± 53.31 | 73.32 ± 36.84 | 18.01 ± 15.85 | 0.027 ± 0.005 | 3.27 ± 1.07 | 25.75 ± 10.68 |
|  |  |  | 2007 | 0.12 | 3.46 | 3.57 | 89.03 | 139.79 ± 47.45 | 86.91 ± 36.86 | 24.63 ± 15.86 | 0.026 ± 0.004 | 3.00 ± 3.57 | 21.41 ± 18.27 |
|  |  |  | 2008 | 0.32 | 3.27 | 3.60 | 93.60 | 138.81 ± 47.31 | 86.64 ± 34.27 | 17.51 ± 13.67 | 0.022 ± 0.003 | 2.61 ± 0.77 | 13.48 ± 6.50 |
|  |  |  | 2009 | 1.06 | 2.89 | 3.95 | 96.14 | 132.83 ± 49.26 | 80.14 ± 28.04 | 22.71 ± 15.02 | 0.025 ± 0.005 | 2.65 ± 0.80 | 17.16 ± 7.92 |
|  |  |  | 2010 | 0.25 | 2.86 | 3.11 | 71.79 | 137.81 ± 54.27 | 69.73 ± 28.29 | 20.07 ± 15.70 | 0.023 ± 0.005 | 2.60 ± 0.62 | 14.10 ± 5.56 |
|  |  |  | 2011 | 0.13 | 2.97 | 3.11 | 79.14 | 142.64 ± 49.65 | 82.41 ± 33.82 | 19.67 ± 14.84 | 0.021 ± 0.003 | 2.31 ± 0.50 | 14.96 ± 5.44 |
|  |  |  | 2012 | 0.29 | 3.11 | 3.41 | 84.44 | 133.91 ± 49.74 | 78.88 ± 34.93 | 14.70 ± 13.76 | 0.023 ± 0.003 | 2.61 ± 0.76 | 16.86 ± 5.28 |
|  |  |  | 2013 | -0.18 | 3.49 | 3.30 | 84.32 | 136.70 ± 51.88 | 84.09 ± 39.20 | 17.96 ± 13.42 | 0.025 ± 0.004 | 2.84 ± 0.92 | 17.82 ± 5.44 |
|  |  |  | 2014 | 0.14 | 3.38 | 3.52 | 78.36 | 124.90 ± 51.33 | 78.95 ± 37.80 | 15.68 ± 12.61 | 0.025 ± 0.004 | 2.80 ± 0.81 | 19.29 ± 6.13 |
|  |  |  | 2015 | 0.75 | 2.84 | 3.57 | 79.98 | 114.15 ± 53.40 | 67.82 ± 33.41 | 24.20 ± 23.64 | 0.025 ± 0.006 | 3.08 ± 2.90 | 22.04 ± 16.24 |
|  |  |  | 2016 | 0.31 | 3.00 | 3.33 | 74.23 | 124.45 ± 54.48 | 70.72 ± 38.22 | 11.86 ± 18.33 | 0.022 ± 0.004 | 3.29 ± 9.25 | 19.56 ± 18.34 |
|  |  |  |  | *0.38 ± 0.29* | *3.21 ± 0.25* | *3.59 ± 0.30* |  | *132.45 ± 51.29* | *77.75 ± 34.45* | *18.29 ± 15.71* | *0.024 ± 0.005* | *2.81 ± 2.71* | *18.96 ± 10.36* |
|  |  |  |  |  |  |  |  |  |  |  |  |  |  |
|  | PDB | Secondary | 2005 | 0.35 | 1.50 | 1.85 | 38.34 | 130.72 ± 49.10 | 89.47 ± 27.71 | 18.10 ± 10.10 | 0.011 ± 0.005 | 1.24 ± 0.34 | 7.75 ± 2.19 |
|  |  | Forest | 2006 | 0.22 | 1.61 | 1.83 | 63.26 | 115.89 ± 51.32 | 78.12 ± 28.58 | 17.33 ± 13.53 | 0.013 ± 0.004 | 1.51 ± 0.38 | 10.29 ± 3.70 |
|  |  |  | 2007 | 0.29 | 2.11 | 2.40 | 66.54 | 131.75 ± 49.05 | 82.75 ± 24.38 | 21.37 ± 12.93 | 0.015 ± 0.005 | 1.84 ± 0.40 | 12.04 ± 4.32 |
|  |  |  | 2008 | 0.07 | 1.84 | 1.91 | 65.98 | 128.03 ± 47.87 | 68.20 ± 17.59 | 20.25 ± 11.04 | 0.013 ± 0.006 | 1.82 ± 0.37 | 11.41 ± 4.30 |
|  |  |  | 2009 | 0.23 | 1.65 | 1.87 | 68.09 | 125.02 ± 50.32 | 72.33 ± 22.12 | 19.93 ± 15.59 | 0.011 ± 0.010 | 1.75 ± 0.67 | 11.37 ± 3.65 |
|  |  |  | 2010 | -0.77 | 1.92 | 1.16 | 34.42 | 138.97 ± 56.51 | 87.93 ± 30.99 | 14.55 ± 9.25 | 0.012 ± 0.003 | 1.40 ± 0.40 | 7.83 ± 3.57 |
|  |  |  | 2011 | -0.68 | 1.99 | 1.31 | 48.34 | 141.85 ± 50.24 | 94.10 ± 32.43 | 19.45 ± 12.33 | 0.010 ± 0.004 | 1.43 ± 0.37 | 8.52 ± 2.94 |
|  |  |  | 2012 | -0.65 | 2.24 | 1.58 | 38.29 | 140.60 ± 50.54 | 94.28 ± 29.36 | 15.43 ± 11.37 | 0.014 ± 0.004 | 1.74 ± 0.46 | 9.96 ± 3.63 |

Table S6 (cont). Year-to-year carbon, energy, and water fluxes of every site. Mean ± stdev for C stocks (NEE, GPP, and Reco) were calculated based on yearly data, while the rest (Rn, LE, H, LUE, WUE, iWUE) were based on daily values.

| **LUI** | **Site** | **Ecosystem type** | **Year** | **NEE** | **GPP** | **Reco** | **Gap-filled** | **Net radiation (Rn)** | **Latent heat (LE)** | **Sensible heat (H)** | **LUE** | **WUE** | **iWUE** |
| --- | --- | --- | --- | --- | --- | --- | --- | --- | --- | --- | --- | --- | --- |
|  | PDB | Secondary | 2013 | -0.65 | 2.23 | 1.58 | 37.59 | 145.91 ± 51.29 | 95.79 ± 31.56 | 16.47 ± 13.81 | 0.014 ± 0.004 | 1.73 ± 0.44 | 9.38 ± 3.66 |
|  |  | Forest | 2014 | -0.24 | 1.76 | 1.52 | 45.61 | 129.38 ± 48.99 | 79.73 ± 32.77 | 20.98 ± 17.70 | 0.013 ± 0.007 | 1.47 ± 0.67 | 9.49 ± 5.57 |
|  |  |  | 2015 | 0.04 | 1.81 | 1.85 | 57.21 | 117.34 ± 51.97 | 73.40 ± 28.23 | 19.69 ± 16.50 | 0.016 ± 0.006 | 1.72 ± 0.46 | 12.37 ± 5.00 |
|  |  |  | 2016 | -0.40 | 2.18 | 1.79 | 66.11 | 127.15 ± 50.29 | 88.56 ± 33.48 | 16.13 ± 12.69 | 0.016 ± 0.005 | 1.74 ± 0.57 | 9.25 ± 4.40 |
|  |  |  |  | *-0.18 ± 0.43* | *1.90 ± 0.25* | *1.72 ± 0.32* |  | *131.10 ± 51.04* | *83.28 ± 29.72* | *18.72 ± 13.71* | *0.013 ± 0.006* | *1.57 ± 0.52* | *9.78 ± 4.27* |
|  |  |  |  |  |  |  |  |  |  |  |  |  |  |
| High (3) | JOP | Oil Palm | 2014 | -0.55 | 1.30 | 1.12 | 40.94 | 136.67 ± 29.84 | 131.99 ± 27.44 | 11.02 ± 5.49 | 0.035 ± 0.014 | 1.85 ± 5.92 | 18.70 ± 42.50 |
|  |  | plantation | 2015 | -0.70 | 4.23 | 3.91 |  | 147.92 ± 56.05 | 121.04 ± 42.71 | 17.62 ± 12.12 | 0.072 ± 0.027 | 3.67 ± 1.37 | 23.10 ± 11.61 |
|  |  |  | 2017 | -0.43 | 2.72 | 3.56 | 52.33 |  | 93.16 ±29.64 | 11.24 ± 6.17 | 0.024 ± 0.011 | 2.32 ± 2.30 | 10.75 ± 13.57 |
|  |  |  | 2018 | -1.26 | 4.02 | 3.44 | 17.02 | 99.71 ± 35.78 | 93.68 ±29.16 | 13.56 ±8.64 | 0.027 ± 0.035 | 4.90 ± 6.65 | 23.51 ± 28.00 |
|  |  |  | 2019 | -0.81 | 4.25 | 3.69 |  | 110.63 ± 25.66 | 98.50 ± 20.93 | 13.08 ±6.95 | 0.033 ± 0.012 | 3.49 ± 1.01 | 24.98 ±11.48 |
|  |  |  | 2020 | -0.52 | 3.94 | 3.06 | 41.96 | 152.00 ± 45.38 | 131.82 ± 35.46 | 21.18 ± 11.57 | 0.033 ± 0.011 | 3.44 ± 1.47 | 22.43 ± 8.02 |
|  |  |  |  | *-0.71 ± 0.30* | *3.41 ± 1.18* | *3.13 ± 1.02* |  | *130.54 ± 47.30* | *111.23 ± 35.67* | *14.91 ± 9.74* | *0.039 ± 0.029* | *3.45 ± 3.75* | *21.79 ± 19.42* |
|  |  |  |  |  |  |  |  |  |  |  |  |  |  |
|  | RFC | Rubber | 2015 | -1.17 | 2.34 | 1.29 |  | 206.90 ± 51.76 | 88.28 ± 36.25 | 33.77 ± 20.58 | 0.019 ± 0.009 | 2.21 ± 0.92 | 16.70 ± 8.91 |
|  |  | plantation | 2016 | -0.96 | 2.44 | 1.54 |  | 207.73 ± 50.76 | 71.67 ± 31.38 | 24.35 ± 22.61 | 0.020 ± 0.011 | 2.70 ± 0.74 | 20.42 ± 11.38 |
|  |  |  | 2017 | -1.38 | 2.68 | 1.21 |  | 196.73 ± 60.75 | 80.17 ± 34.44 | 26.02 ± 21.45 | 0.025 ± 0.018 | 2.69 ± 0.74 | 16.99 ± 9.68 |
|  |  |  | 2018 | -1.39 | 2.69 | 1.35 |  | 201.04 ± 48.39 | 92.73 ±45.65 | 23.04 ± 19.54 | 0.024 ± 0.012 | 2.23 ± 0.68 | 18.44 ± 8.02 |
|  |  |  |  | *-1.23 ± 0.20* | *2.54 ± 0.18* | *1.35 ± 0.14* |  | *203.01 ± 53.26* | *83.64 ± 36.10* | *26.85 ± 21.37* | *0.022 ± 0.013* | *2.46 ± 0.81* | *18.14 ± 9.71* |
|  |  |  |  |  |  |  |  |  |  |  |  |  |  |
|  | RFN | Rubber | 2017 | -1.30 | 2.36 | 1.03 |  | 182.25 ± 61.60 | 90.91 ± 34.70 | 91.94 ± 27.38 | 0.024 ± 0.014 | 2.17 ± 0.70 | 11.31 ± 5.20 |
|  |  | plantation | 2018 | -0.92 | 2.02 | 1.05 |  | 191.20 ± 58.47 | 81.85 ± 27.50 | 86.70 ± 28.30 | 0.020 ± 0.010 | 2.01 ± 0.64 | 10.56 ± 5.94 |
|  |  |  |  | *-1.11 ± 0.27* | *2.19 ± 0.24* | *1.04 ± 0.01* |  | *186.72 ± 60.18* | *86.39 ± 31.61* | *89.09 ± 27.93* | *0.022 ± 0.012* | *2.09 ± 0.68* | *10.93 ± 5.59* |
|  |  |  |  |  |  |  |  |  |  |  |  |  |  |
|  | SBW | Oil Palm | 2011 | 1.22 | 2.47 | 3.69 | 58.31 |  |  |  |  |  |  |
|  |  | plantation | 2012 | 0.85 | 2.75 | 3.59 | 74.20 |  |  |  |  |  |  |
|  |  |  | 2013 | 1.06 | 2.46 | 3.51 | 76.18 |  |  |  |  |  |  |
|  |  |  | 2014 | 0.81 | 2.37 | 3.18 | 72.48 |  |  |  |  |  |  |
|  |  |  |  | *0.99 ± 0.19* | *2.51 ± 0.16* | *3.49 ± 0.22* |  |  |  |  |  |  |  |
|  |  |  |  |  |  |  |  |  |  |  |  |  |  |
|  |  |  |  |  |  |  |  |  |  |  |  |  |  |

Table S6 (cont). Year-to-year carbon, energy, and water fluxes of every site. Mean ± stdev for C stocks (NEE, GPP, and Reco) were calculated based on yearly data, while the rest (Rn, LE, H, LUE, WUE, iWUE) were based on daily values.

| **LUI** | **Site** | **Ecosystem type** | **Year** | **NEE** | **GPP** | **Reco** | **Gap-filled** | **Net radiation (Rn)** | **Latent heat (LE)** | **Sensible heat (H)** | **LUE** | **WUE** | **iWUE** |
| --- | --- | --- | --- | --- | --- | --- | --- | --- | --- | --- | --- | --- | --- |
|  |  |  |  |  |  |  |  |  |  |  |  |  |  |
|  | KAP | Acacia | 2016 | 1.32 |  |  |  |  |  |  |  |  |  |
|  |  | plantation | 2017 | -0.15 |  |  |  |  |  |  |  |  |  |
|  |  |  | 2018 | -0.24 |  |  |  |  |  |  |  |  |  |
|  |  |  | 2019 | 0.07 |  |  |  |  |  |  |  |  |  |
|  |  |  | 2020 | 0.07 |  |  |  |  |  |  |  |  |  |
|  |  |  | 2021 | 0.32 |  |  |  |  |  |  |  |  |  |
|  |  |  |  | 0.26 *± 0.12* |  |  |  |  |  |  |  |  |  |
|  |  |  |  |  |  |  |  |  |  |  |  |  |  |
|  |  |  |  |  |  |  |  |  |  |  |  |  |  |

Table S7. Year-to-year meteorological parameters of every site. Mean ± stdev for most of the data (PAR, Tair, RH, VPD, SWC, REW) were calculated based on daily values, while precipitation and ET were based on yearly data.

| **LUI** | **Site** | **Ecosystem type** | **Year** | **PAR** | **Tair** | **RH** | **VPD** | **SWC** | **REW** | **Precipitation** | **ET** |
| --- | --- | --- | --- | --- | --- | --- | --- | --- | --- | --- | --- |
|  |  |  |  | (µmol m^-2^day^-1^) | (°C) | (%) | (hPa) | (m^3^m^-3^) |  | (mm yr^-1^) | (mm yr^-1^) |
| Low (1) | LHP | Forest | 2010 | 330.45 ± 71.98 | 26.14 ± 0.94 | 82.19 ± 4.04 | 6.53 ± 1.70 | NA | NA | 3016.50 | 212.66 |
|  |  |  | 2011 | 307.49 ± 74.15 | 25.92 ± 1.00 | 83.37 ± 4.37 | 6.03 ± 1.87 | NA | NA | 3080.00 | 205.92 |
|  |  |  | 2012 | 327.12 ± 71.17 | 26.14 ± 0.97 | 82.38 ± 5.27 | 6.50 ± 2.28 | 0.301 ± 0.022 | NA | 2958.50 | 677.60 |
|  |  |  | 2013 | 326.61 ± 73.89 | 26.10 ± 0.98 | 82.68 ± 4.63 | 6.35 ± 2.01 | 0.302 ± 0.023 | 0.510 ± 0.066 | 2918.00 | 723.64 |
|  |  |  | 2014 | 323.01 ± 81.59 | 25.98 ± 1.08 | 82.87 ± 4.05 | 6.19 ± 1.75 | 0.289 ± 0.027 | 0.491 ± 0.046 | 2345.00 | 607.06 |
|  |  |  | 2015 | 332.89 ± 74.58 | 26.22 ± 1.11 | 81.23 ± 4.30 | 6.87 ± 1.99 | 0.283 ± 0.029 | 0.492 ± 0.055 | 2322.00 | 605.29 |
|  |  |  | 2016 | 334.79 ± 70.95 | 26.57 ± 0.97 | 81.73 ± 4.39 | 6.85 ± 1.95 | 0.272 ± 0.027 | 0.487 ± 0.042 | 2065.00 | 711.82 |
|  |  |  | 2017 | 324.32 ± 73.66 | 26.15 ± 0.96 | 82.38 ± 4.46 | 6.46 ± 1.94 | 0.311 ± 0.021 | 0.486 ± 0.054 | 3465.50 | 320.01 |
|  |  |  | 2018 | 328.66 ± 72.56 | 26.25 ± 1.00 | 80.51 ± 3.58 | 7.12 ± 1.70 | 0.308 ± 0.023 | 0.528 ± 0.070 | 3007.00 | 211.00 |
|  |  |  | 2019 | 333.14 ± 71.62 | 26.40 ± 1.02 | 79.45 ± 4.08 | 7.55 ± 1.90 | 0.262 ± 0.035 | NA | 2333.50 | 134.99 |
|  |  | Mean ± stdev | | *326.85 ± 73.95* | *26.19 ± 1.02* | *81.88 ± 4.47* | *6.65 ± 1.96* | *0.290 ± 0.030* | *0.500 ± 0.060* | *2751.10 ± 449.63* | *441.00 ± 243.21* |
|  |  |  | |  |  |  |  |  |  |  |  |
|  | MKL | Forest | 2003 | 378.64 ± 106.08 | 24.91 ± 1.86 | 57.74 ± 8.01 | 8.86 ± 3.70 | 0.336 ± 0.076 | 0.495 ± 0.068 | 1708.00 | 613.15 |
|  |  |  | 2004 | 361.19 ± 92.05 | 25.21 ± 1.90 | 70.63 ± 11.72 | 10.39 ± 4.73 | 0.291 ± 0.075 | 0.505 ± 0.047 | 738.00 | 540.51 |
|  |  |  |  | *369.90 ± 99.62* | *25.06 ± 1.88* | *64.19 ± 11.93* | *9.38 ± 4.36* | *0.310 ± 0.080* | *0.500 ± 0.060* | *1223.00 ± 685.89* | *576.83 ± 51.37* |
|  |  |  |  |  |  |  |  |  |  |  |  |
|  | PSO | Forest | 2003 | 443.68 ± 108.84 | 25.63 ± 1.05 |  | 6.15 ± 2.37 | 0.366 ± 0.027 | 0.473 ± 0.145 | 1895.81 | 1417.30 |
|  |  |  | 2004 | 433.63 ± 99.57 | 25.57 ± 1.08 |  | 6.51 ± 2.42 | 0.359 ± 0.027 | 0.508 ± 0.129 | 1655.12 | 1360.62 |
|  |  |  | 2005 | 460.68 ± 106.07 | 25.68 ± 1.12 |  | 7.30 ± 2.80 | 0.360 ± 0.040 | 0.495 ± 0.131 | 1648.72 | 1343.60 |
|  |  |  | 2006 | 426.38 ± 101.06 | 25.28 ± 0.93 |  | 6.06 ± 2.33 | 0.384 ± 0.025 | 0.479 ± 0.117 | 2058.50 | 1286.13 |
|  |  |  | 2007 | 414.17 ± 106.87 | 25.13 ± 1.05 |  | 5.79 ± 2.27 | 0.389 ± 0.020 | 0.493 ± 0.113 | 2110.50 | 1240.28 |
|  |  |  | 2008 | 444.72 ± 104.73 | 24.89 ± 0.92 |  | 5.59 ± 2.00 | 0.405 ± 0.018 | 0.472 ± 0.146 | 2234.50 | 1323.69 |
|  |  |  | 2009 | 444.39 ± 98.33 | 25.18 ± 1.18 |  | 6.63 ± 2.56 | 0.362 ± 0.029 | 0.513 ± 0.121 | 1450.78 | 1195.11 |
|  |  |  |  | *438.23 ± 104.51* | *25.34 ± 1.08* |  | *6.29 ± 2.46* | *0.370 ± 0.030* | *0.490 ± 0.130* | *1864.85 ± 287.98* | *1309 ± 75.30* |
|  |  |  |  |  |  |  |  |  |  |  |  |
|  | SKR | Forest | 2001 | 424.03 ± 118.69 | 24.37 ± 2.53 | 76.75 ± 8.73 | 7.99 ± 3.41 | 0.102 ± 0.022 | 0.496 ± 0.088 | 3049.70 | 372.13 |
|  |  |  | 2002 | 433.17 ± 111.64 | 24.38 ± 2.09 | 74.25 ± 9.74 | 8.97 ± 3.66 | 0.113 ± 0.033 | 0.486 ± 0.091 | 1812.71 | 715.12 |
|  |  |  | 2003 | 464.23 ± 113.66 | 23.96 ± 2.45 | 73.48 ± 9.17 | 9.32 ± 3.41 | 0.119 ± 0.024 | 0.554 ± 0.220 | 1152.30 | 870.04 |
|  |  |  |  | *441.26 ± 115.67* | *24.23 ± 2.36* | *74.73 ± 9.34* | *8.80 ± 3.54* | *0.110 ± 0.030* | *0.510 ± 0.150* | *2004.90 ± 963.19* | *652.43 ± 254.81* |

Table S7 (cont). Year-to-year meteorological parameters of every site. Mean ± stdev for most of the data (PAR, Tair, RH, VPD, SWC, REW) were calculated based on daily values, while precipitation and ET were based on yearly data.

| **LUI** | **Site** | **Ecosystem type** | **Year** | **PAR** | **Tair** | **RH** | **VPD** | **SWC** | **REW** | **Precipitation** | **ET** |
| --- | --- | --- | --- | --- | --- | --- | --- | --- | --- | --- | --- |
|  |  |  |  | (µmol m^-2^day^-1^) | (°C) | (%) | (hPa) | (m^3^m^-3^) |  | (mm yr^-1^) | (mm yr^-1^) |
|  | KIF | Forest | 2017 |  | 26.71 ± 0.95 |  | 7.32 ± 2.93 |  |  | 2020 | 1537 |
|  |  |  | 2018 |  | 26.46 ± 0.86 |  | 6.90 ± 2.60 |  |  | 1756 | 1592 |
|  |  |  | 2019 |  | 27.12 ± 0.85 |  | 7.46 ± 2.80 |  |  | 1496 | 1547 |
|  |  |  | 2020 |  | 27.24 ± 0.83 |  | 7.87 ± 3.17 |  |  | 2249 | 1502 |
|  |  |  | 2021 |  | *27.30* ± 0.80 |  |  |  |  | *1895* | *1471* |
|  |  |  |  |  | *26.83 ± 0.92* |  | *7.29 ± 2.82* |  |  | *1883* | *1530* |
|  |  |  |  |  |  |  |  |  |  |  |  |
|  | PUF | Forest | 2005 | 436.4 ± 96.38 | 26.15 ± 0.9 | 80.83 ± 4.64 | 7.26 ± 2.12 |  |  | 2434.90 | 1397.50 |
|  |  |  | 2006 | 454.67 ± 129.72 | 25.88 ± 1.02 | 79.45 ± 5.46 | 7.73 ± 2.4 |  |  | 2031.60 | 1522.91 |
|  |  |  | 2007 | 429.7 ± 93.63 | 25.67 ± 1.37 | 79.54 ± 4.42 | 7.59 ± 2.14 |  |  | 3031.10 | 1566.38 |
|  |  |  | 2008 | 457.87 ± 100.3 | 25.96 ± 0.96 | 80.86 ± 4.68 | 7.26 ± 2.18 |  |  | 2623.00 | 1437.60 |
|  |  |  | 2009 | 469.62 ± 107.11 | 26.61 ± 0.98 | 79.93 ± 5.13 | 7.92 ± 2.38 |  |  | 2390.40 | 1369.84 |
|  |  |  | 2010 | 444.64 ± 111.56 | 26.31 ± 1.08 | 85.9 ± 5.01 | 5.63 ± 2.31 |  |  | 3806.50 | 1389.22 |
|  |  |  | 2011 | 451.43 ± 105.17 | 26.21 ± 0.92 | 83.52 ± 4.89 | 6.48 ± 2.19 |  |  | 2843.10 | 1425.88 |
|  |  |  | 2012 | 449.2 ± 100 | 26.22 ± 0.94 | 83.55 ± 4.9 | 6.47 ± 2.23 |  |  | 2507.10 | 1417.61 |
|  |  |  | 2013 | 467.19 ± 108.41 | 26.37 ± 1.02 | 82.85 ± 4.87 | 6.74 ± 2.28 |  |  | 2992.00 | 1459.25 |
|  |  |  | 2014 | 460.78 ± 109.72 | 25.16 ± 1.31 | 82.18 ± 5.34 | 6.63 ± 2.44 |  |  | 2398.20 | 1385.67 |
|  |  |  | 2015 | 429.6 ± 137.62 | 25.59 ± 1.29 | 81.65 ± 4.81 | 6.99 ± 2.27 |  |  | 2149.00 | 1237.23 |
|  |  |  | 2016 | 477.16 ± 116.74 | 26.78 ± 1.08 | 84.27 ± 6.25 | 6.46 ± 2.82 |  |  | 2974.00 | 1321.79 |
|  |  |  | 2017 | 478.95 ± 114.23 | 26.34 ± 0.94 | 84.97 ± 4.82 | 5.94 ± 2.18 |  |  | 2772.30 | 1528.74 |
|  |  |  |  | *454.40 ± 16.45* | *26.10 ± 0.43* | *82.27 ± 2.10* | *6.85 ± 0.68* |  |  | *2688.71 ± 462.69* | *1419.97 ± 88.47* |
|  |  |  |  |  |  |  |  |  |  |  |  |
| Medium | BKS | Secondary | 2001 | 302.94 ± 237.25 | 25.84 ± 0.99 | 87.19 ± 3.65 | 4.94 ± 1.61 | 0.310 ± 0.040 | 0.520 ± 0.090 | 2516.00 | NA |
| (2) |  | forest | 2002 | 336.62 ± 92.85 | 26.07 ± 1.10 | 86.28 ± 4.75 | 5.68 ± 2.12 | 0.290 ± 0.040 | 0.530 ± 0.070 | 2014.00 | 302.87 |
|  |  |  |  | *319.73 ± 180.99* | *25.95 ± 1.05* | *86.74 ± 4.25* | *5.30 ± 1.91* | *0.300 ± 0.040* | *0.530 ± 0.080* | *2265.00 ± 354.97* |  |
|  |  |  |  |  |  |  |  |  |  |  |  |
|  | DFR | Secondary | 2015 | 328.81 ± 113.05 | 27.41 ± 2.40 | 73.24 ± 9.58 | 11.32 ± 4.88 | 0.080 ± 0.040 | 0.460 ± 0.130 | 670.9 | 1233.71 |
|  |  |  | 2016 | 318.25 ± 111.18 | 27.32 ± 2.32 | 74.84 ± 13.30 | 22.4 ± 47.86 | 0.080 ± 0.050 | 0.460 ± 0.130 | 704.88 | 1121.58 |
|  |  |  | 2017 | 323.35 ± 110.91 | 27.07 ± 1.83 | 80.25 ± 11.81 | 8.35 ± 5.39 | 0.070 ± 0.040 | 0.460 ± 0.130 | 1002.13 | 1484.89 |
|  |  |  |  | *323.43 ± 111.73* | *27.27 ± 2.20* | *76.09 ± 11.96* | *13.78 ± 27.67* | *0.080 ± 0.040* | *0.460 ± 0.130* | *792.64 ± 182.22* | *1280 ± 186.04* |

Table S7 (cont). Year-to-year meteorological parameters of every site. Mean ± stdev for most of the data (PAR, Tair, RH, VPD, SWC, REW) were calculated based on daily values, while precipitation and ET were based on yearly data.

| **LUI** | **Site** | **Ecosystem type** | **Year** | **PAR** | **Tair** | **RH** | **VPD** | **SWC** | **REW** | **Precipitation** | **ET** |
| --- | --- | --- | --- | --- | --- | --- | --- | --- | --- | --- | --- |
|  |  |  |  | (µmol m^-2^day^-1^) | (°C) | (%) | (hPa) | (m^3^m^-3^) |  | (mm yr^-1^) | (mm yr^-1^) |
|  | KDF | Secondary | 2016 | 749.96 ± 200.51 | 26.88 ± 0.84 |  | 7.97 ± 2.91 |  |  | *2142* | *1372* |
|  |  | Degraded | 2017 | 742.87 ± 200.09 | 26.76 ± 0.88 |  | 7.16 ± 2.91 |  |  | *1837* | *1371* |
|  |  | forest | 2018 | 708.36 ± 186.38 | 26.67 ± 0.88 |  | 6.92 ± 2.76 |  |  | *1325* | *1539* |
|  |  |  | 2019 | 723.77 ± 184.95 | 27.39 ± 0.88 |  | 7.89 ± 2.91 |  |  | *1763* | *1496* |
|  |  |  | 2020 | 737.52 ± 206.05 | 28.43 ± 1.22 |  | 8.33 ± 3.14 |  |  | *2206* | *1351* |
|  |  |  | 2021 |  | *27.80* ± 0.20 |  |  |  |  | *1347* | *869* |
|  |  |  |  | *728.84 ± 194.39* | *27.19 ± 1.14* |  | *7.53 ± 2.96* |  |  | *1942* | *1411* |
|  |  |  |  |  |  |  |  |  |  |  |  |
|  | PDF | Secondary | 2002 | 372.76 ± 142.91 | 26.72 ± 1.03 | 80.29 ± 6.1 | 7.78 ± 2.8 | NA | NA | 1867.6 | 1263.53 |
|  |  | Forest | 2003 | 423.67 ± 145.47 | 26.33 ± 1.23 | 78.61 ± 5.35 | 8.21 ± 2.71 | 0.300 ± 0.060 | 0.470 ± 0.130 | 2309.5 | 1194.85 |
|  |  |  | 2004 | 386.16 ± 120.28 | 25.88 ± 1.18 | 79.71 ± 5.28 | 7.66 ± 2.58 | 0.280 ± 0.050 | 0.500 ± 0.140 | 2576.4 | 1229.42 |
|  |  |  | 2005 | 392.24 ± 129.6 | 26.07 ± 0.97 | 80.34 ± 5.14 | 7.35 ± 2.43 | 0.300 ± 0.030 | 0.480 ± 0.120 | 2637.6 | 1229.78 |
|  |  |  | 2006 | 392.15 ± 158.04 | 25.9 ± 0.98 | 78.23 ± 5.81 | 8.06 ± 2.68 | 0.320 ± 0.030 | 0.490 ± 0.120 | 1991.9 | 1173.19 |
|  |  |  | 2007 | 378.87 ± 128.35 | 25.86 ± 1 | 79.1 ± 5.39 | 7.68 ± 2.49 | NA | NA | 2570.2 | 1319.18 |
|  |  |  | 2008 | 439.93 ± 145.09 | 26.05 ± 1.03 | 86.81 ± 5.48 | 5.23 ± 2.44 | NA | NA | 2623 | 1307.8 |
|  |  |  | 2009 | 384.45 ± 124.3 | 26.37 ± 1.02 | 83.52 ± 5.75 | 6.53 ± 2.62 |  |  | 2253 | 1169.97 |
|  |  |  | 2010 | 358.37 ± 127.48 | 26.34 ± 1.12 | 85.38 ± 5.58 | 5.7 ± 2.6 |  |  | 3750.1 | 1133.5 |
|  |  |  | 2011 | 408.55 ± 133.63 | 26.3 ± 0.97 | 82.84 ± 5.52 | 6.64 ± 2.52 |  |  | 2962.1 | 1284.92 |
|  |  |  | 2012 | 405.45 ± 134.19 | 26.13 ± 1.05 | 82.22 ± 5.64 | 6.83 ± 2.59 |  |  | 2460.5 | 1236.22 |
|  |  |  | 2013 | 420.99 ± 138.61 | 26.01 ± 1.2 | 82.23 ± 5.44 | 6.75 ± 2.52 |  |  | 2775.9 | 1324.12 |
|  |  |  | 2014 | 409.22 ± 141.79 | 25.88 ± 1.01 | 80.82 ± 5.42 | 7.22 ± 2.49 |  |  | 2185.9 | 1279.35 |
|  |  |  | 2015 | 371.95 ± 156.7 | 25.94 ± 0.92 | 80.18 ± 5.85 | 7.5 ± 2.56 |  |  | 2282.8 | 1081.91 |
|  |  |  | 2016 | 409.93 ± 152.4 | 26.75 ± 1.12 | 81.84 ± 6.25 | 7.16 ± 2.91 |  |  | 3116.4 | NA |
|  |  |  |  | *397.08 ± 140.47* | *26.17 ± 1.09* | *81.56 ± 6.06* | *7.05 ± 2.71* | *0.300 ± 0.050* | *0.490 ± 0.130* | *2557.53* ± 472.86 | *1230.55* ± 72.74 |
|  |  |  |  |  |  |  |  |  |  |  |  |
|  |  |  |  |  |  |  |  |  |  |  |  |

Table S7 (cont). Year-to-year meteorological parameters of every site. Mean ± stdev for most of the data (PAR, Tair, RH, VPD, SWC, REW) were calculated based on daily values, while precipitation and ET were based on yearly data.

| **LUI** | **Site** | **Ecosystem type** | **Year** | **PAR** | **Tair** | **RH** | **VPD** | **SWC** | **REW** | **Precipitation** | **ET** |
| --- | --- | --- | --- | --- | --- | --- | --- | --- | --- | --- | --- |
|  |  |  |  | (µmol m^-2^day^-1^) | (°C) | (%) | (hPa) | (m^3^m^-3^) |  | (mm yr^-1^) | (mm yr^-1^) |
|  | PDB | Secondary | 2005 | 383.83 ± 129.70 | 26.62 ± 0.95 | 83.54 ± 4.57 | 6.71 ± 2.43 |  |  | 2637.60 | 1239 |
|  |  | Forest | 2006 | 362.00 ± 142.07 | 26.36 ± 0.98 | 82.61 ± 5.34 | 7.12 ± 2.74 |  |  | 1991.90 | 1091.22 |
|  |  |  | 2007 | 401.45 ± 134.31 | 26.49 ± 1.02 | 83.34 ± 5.13 | 6.84 ± 2.68 |  |  | 2570.20 | 1161.79 |
|  |  |  | 2008 | 404.92 ± 132.26 | 26.03 ± 1.05 | 83.97 ± 4.98 | 6.49 ± 2.57 |  |  | 2614.60 | 985.77 |
|  |  |  | 2009 | 451.82 ± 162.00 | 26.31 ± 1.09 | 82.78 ± 5.05 | 7.49 ± 2.76 |  |  | 2253.00 | NA |
|  |  |  | 2010 | 427.34 ± 153.96 | 25.87 ± 1.56 | 85.70 ± 5.08 | 5.75 ± 2.54 |  |  | 3750.10 | 1269.44 |
|  |  |  | 2011 | 462.96 ± 145.97 | 26.09 ± 1.29 | 84.70 ± 4.74 | 6.26 ± 2.37 |  |  | 3019.90 | 1329.99 |
|  |  |  | 2012 | 438.10 ± 143.56 | 25.94 ± 1.19 | 85.42 ± 4.88 | 6.07 ± 2.52 |  |  | 2460.50 | 1312.34 |
|  |  |  | 2013 | 427.64 ± 141.76 | 26.37 ± 1.17 | 86.52 ± 4.83 | 5.84 ± 2.69 |  |  | 2775.90 | 1331.70 |
|  |  |  | 2014 | 371.75 ± 135.59 | 26.80 ± 1.05 | 83.07 ± 6.44 | 7.30 ± 3.29 |  |  | 2219.80 | 1139.86 |
|  |  |  | 2015 | 344.33 ± 148.26 | 26.61 ± 1.00 | 82.42 ± 5.93 | 7.42 ± 2.98 |  |  | 2389.20 | 1065.88 |
|  |  |  | 2016 | 380.89 ± 143.84 | 26.77 ± 1.14 | 87.19 ± 5.40 | 5.67 ± 2.96 |  |  | 3116.40 | NA |
|  |  |  |  | *404.27 ± 145.91* | *26.36 ± 1.17* | *84.14 ± 5.51* | *6.64 ± 2.81* |  |  | *2649.93 ± 473.66* | *1192.70 ± 121.78* |
|  |  |  |  |  |  |  |  |  |  |  |  |
| High (3) | JOP | Oil Palm | 2014 | 179.30 ± 208.22 | 26.31 ± 0.92 | 89.42 ± 4.48 | 5.91 ± 2.46 | 0.270 ± 0.020 | 0.500 ± 0.090 | 3071.40 | 834.63 |
|  |  | plantation | 2015 | 406.27 ± 109.71 | 26.87 ± 0.93 | 85.61 ± 5.44 | 6.33 ± 2.61 | 0.270 ± 0.020 | 0.490 ± 0.100 | 3860.10 | 1231.01 |
|  |  |  | 2017 | 581.61 ± 211.50 | 26.45 ± 0.88 | 90.98 ± 3.45 | 4.97 ± 1.99 | 0.490 ± 0.290 | 0.510 ± 0.100 | 2497.00 | NA |
|  |  |  | 2018 | 446.19 ± 134.93 | 26.32 ± 0.98 | 88.39± 5.04 | 5.34 ± 2.24 | 0.300 ± 0.010 | 0.500 ± 0.090 | 3985.00 | 932.38 |
|  |  |  | 2019 | 345.27 ± 121.88 | 26.24 ± 0.78 | 85.15 ± 5.56 | 7.38 ± 2.85 | 0.270 ± 0.940 | 0.530 ± 0.100 | 1634.00 | 1232.18 |
|  |  |  | 2020 | 325.60 ± 89.86 | 26.50 ± 0.87 | 87.97 ± 3.93 | 7.07 ± 2.17 | 0.627 ± 1.770 | 0.450 ± 0.080 | 1257.00 | 853.86 |
|  |  |  |  | *386.90 ± 197.01* | *26.52 ± 0.94* | *87.79 ± 5.06* | *6.16 ± 2.54* | *0.371 ± 0.152* | *0.500 ± 0.100* | *2593.49 ± 1083.06* | *1016.81 ± 199.46* |
|  |  |  |  |  |  |  |  |  |  |  |  |
|  | RFN | Rubber | 2017 | 332.30 ± 114.59 | 26.37 ± 1.06 | 85.68 ± 7.20 | 5.74 ± 3.02 | 0.310 ± 0.020 | 0.430 ± 0.060 | 4234.20 | 1146.95 |
|  |  | plantation | 2018 | 315.04 ± 97.06 | 26.49 ± 1.07 | 86.42 ± 1.07 | 5.58 ± 3.06 | 0.300 ± 0.030 | 0.430 ± 0.060 | 2642.40 | 1032.46 |
|  |  |  |  | *323.67 ± 106.47* | *26.43 ± 1.07* | *86.05 ± 7.24* | *5.66 ± 3.04* | *0.310 ± 0.020* | *0.430 ± 0.050* | *3438.30 ± 1125.27* | *1089.71 ± 80.96* |
|  |  |  |  |  |  |  |  |  |  |  |  |
|  | SBW | Oil Palm | 2011 | 370.14 ± 99.55 | 25.92 ± 1.08 | 91.76 ± 4.20 | 3.43 ± 1.92 | 0.580 ± 0.050 | 0.470 ± 0.120 | 2850.20 |  |
|  |  | plantation | 2012 | 351.95 ± 91.41 | 25.63 ± 0.96 | 92.06 ± 4.06 | 3.29 ± 1.84 | 0.600 ± 0.040 | 0.480 ± 0.130 | 3034.00 |  |
|  |  |  | 2013 | 331.89 ± 104.74 | 26.01 ± 1.10 | 91.70 ± 4.75 | 3.53 ± 2.26 | 0.520 ± 0.060 | 0.470 ± 0.120 | 2854.40 |  |
|  |  |  | 2014 | 288.20 ± 76.09 | 25.86 ± 1.18 | 91.53 ± 4.04 | 3.51 ± 1.84 | 0.510 ± 0.120 | 0.490 ± 0.140 | 2483.70 |  |
|  |  |  |  | *333.34 ± 98.72* | *25.85 ± 1.08* | *91.79 ± 4.24* | *3.42 ± 1.96* | *0.561 ± 0.074* | *0.476 ± 0.127* | *2805.58 ± 231.05* |  |

Table S7 (cont). Year-to-year meteorological parameters of every site. Mean ± stdev for most of the data (PAR, Tair, RH, VPD, SWC, REW) were calculated based on daily values, while precipitation and ET were based on yearly data.

| **LUI** | **Site** | **Ecosystem type** | **Year** | **PAR** | **Tair** | **RH** | **VPD** | **SWC** | **REW** | **Precipitation** | **ET** |
| --- | --- | --- | --- | --- | --- | --- | --- | --- | --- | --- | --- |
|  |  |  |  | (µmol m^-2^day^-1^) | (°C) | (%) | (hPa) | (m^3^m^-3^) |  | (mm yr^-1^) | (mm yr^-1^) |
|  |  |  |  |  |  |  |  |  |  |  |  |
|  | KAP | Acacia | 2016 |  | 27.20 ± 1.00 |  |  |  |  | 2687 | 1465 |
|  |  | plantation | 2017 |  | 26.90 ± 0.90 |  |  |  |  | 1651 | 1527 |
|  |  |  | 2018 |  | 27.00 ± 0.80 |  |  |  |  | 2177 | 1597 |
|  |  |  | 2019 |  | 27.00 ± 1.20 |  |  |  |  | 2232 | 1635 |
|  |  |  | 2020 |  | 26.60 ± 1.20 |  |  |  |  | 1655 | 843 |
|  |  |  |  |  | *27.00 ± 1.00* |  |  |  |  | *2228* | *1514* |
|  |  |  |  |  |  |  |  |  |  |  |  |


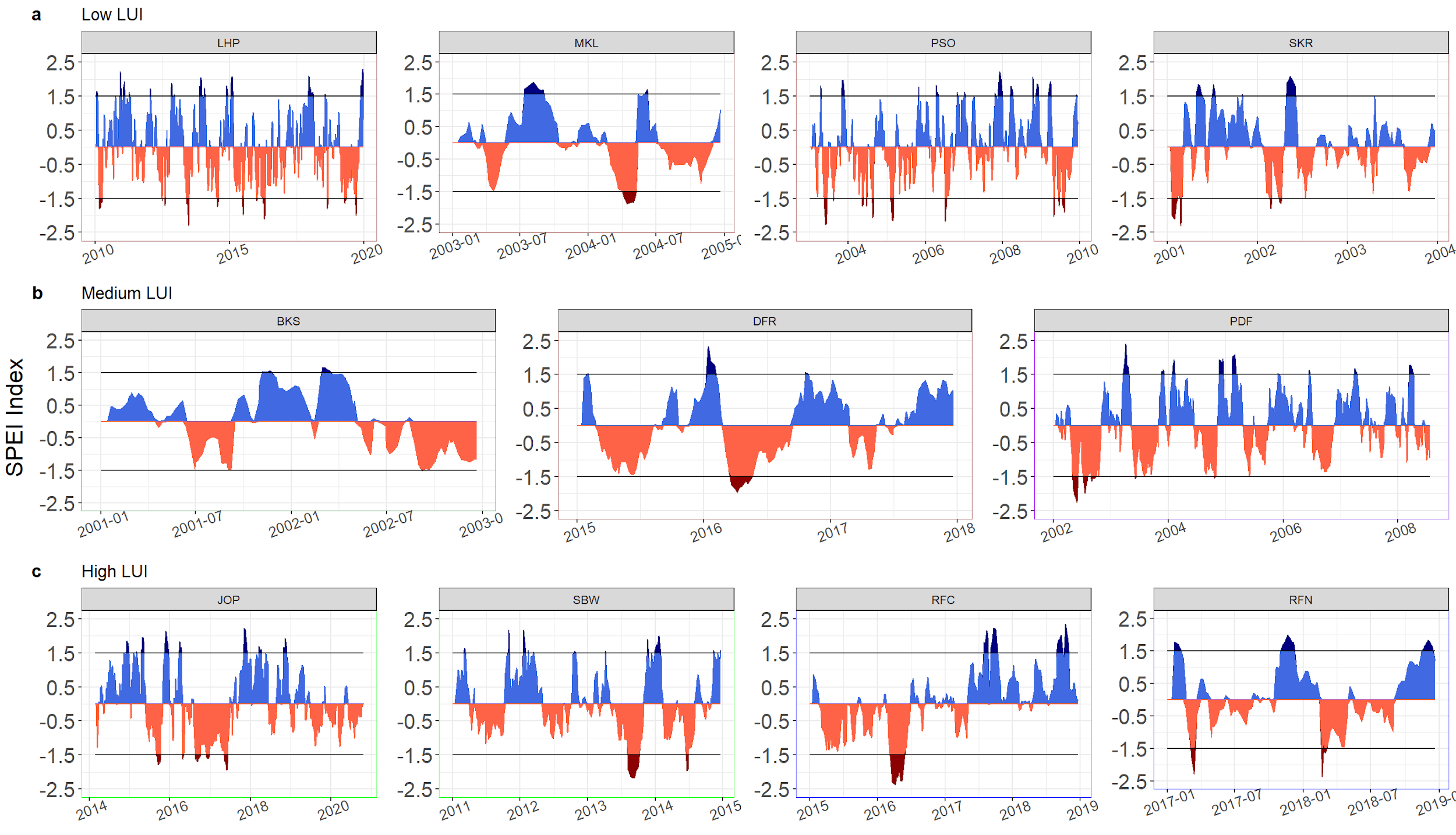


Fig S1. SPEI index for flux tower sites with different LUI in Southeast Asia. The blue and red graphs refer to wet and dry events. The horizontal line represents (1.5 value) the threshold for severe to extreme conditions based on the SPEI category (Table 3).

Compiled Fig S2. Contribution of meteorological drivers, energy, and water fluxes to **NEE** under different LUI.

| LHP: Low (NEE) | MKL: Low (NEE) |
| --- | --- |
| 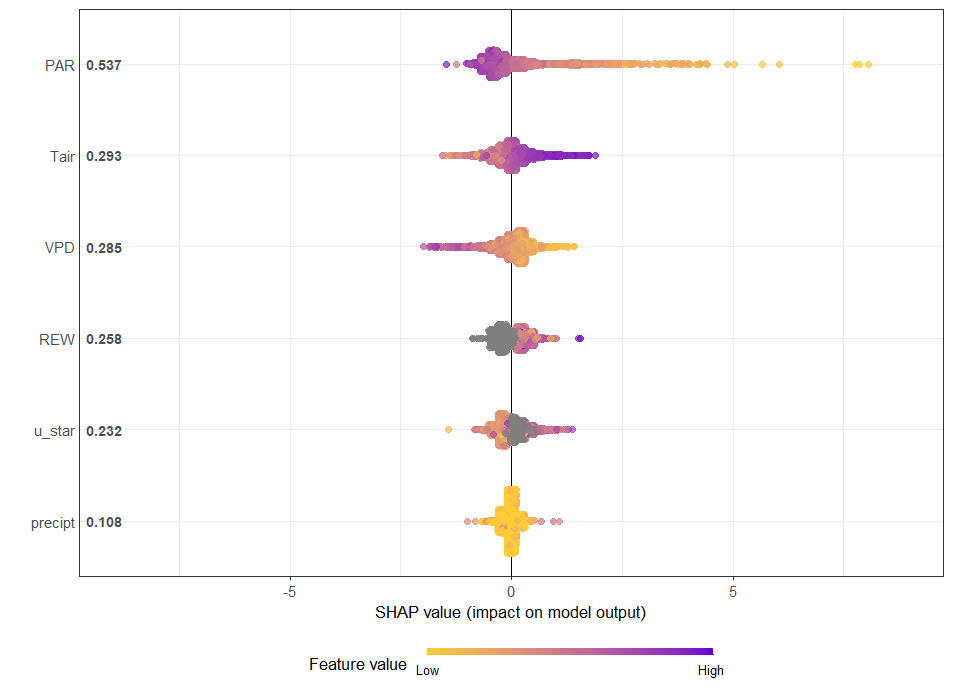 | 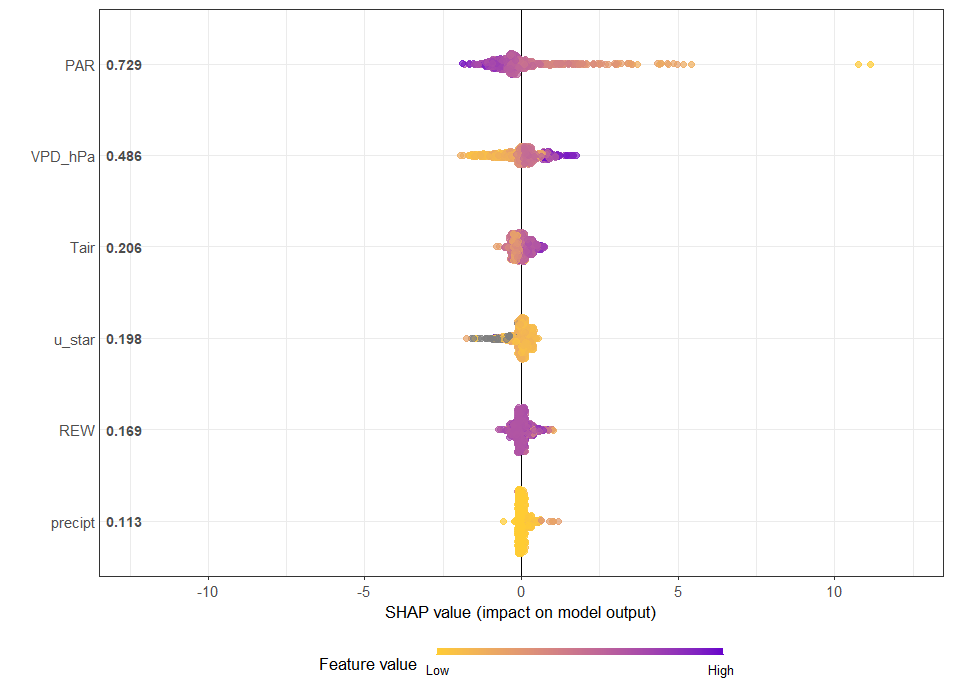 |
| SKR: Low (NEE) | PSO: Low (NEE) |
| 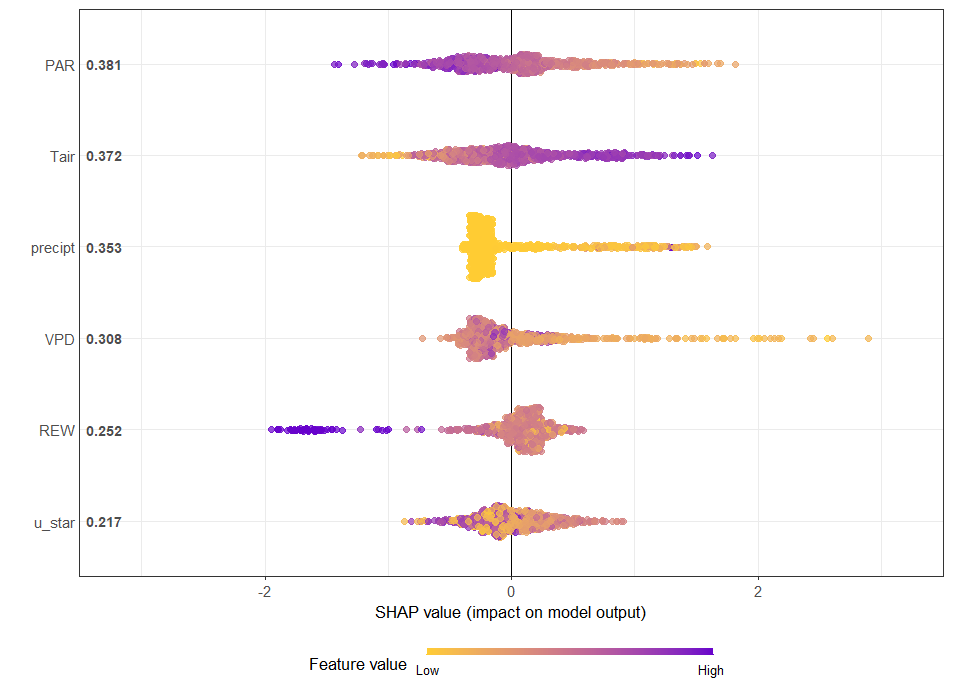 | 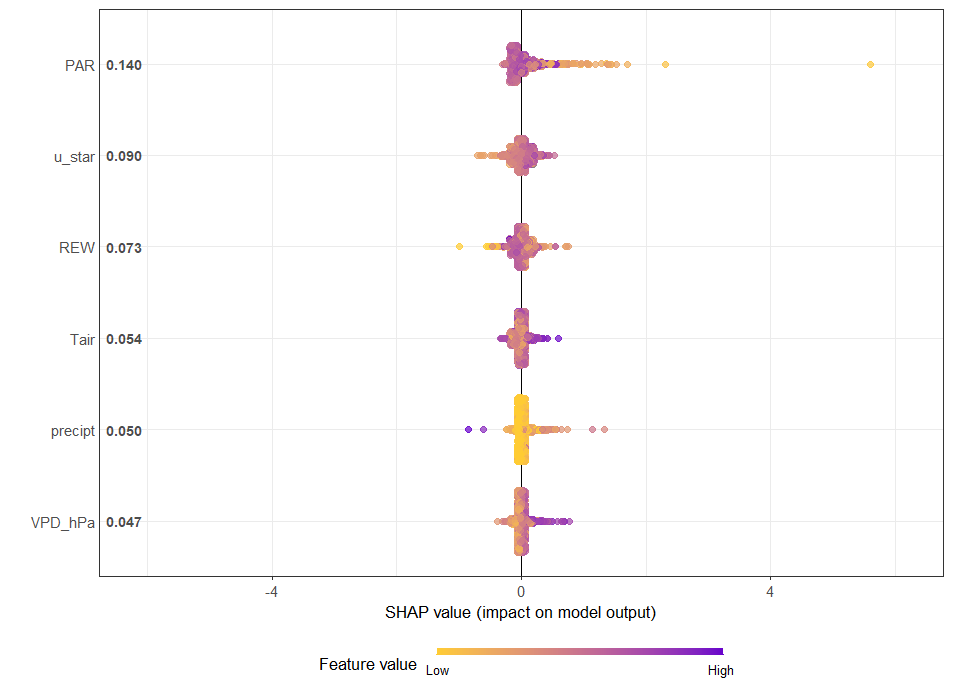 |

Compiled Fig S2 (cont). Contribution of meteorological drivers, energy, and water fluxes to **NEE** under different LUI.

| BKS: Medium (NEE) | DFR: Medium (NEE) |
| --- | --- |
| 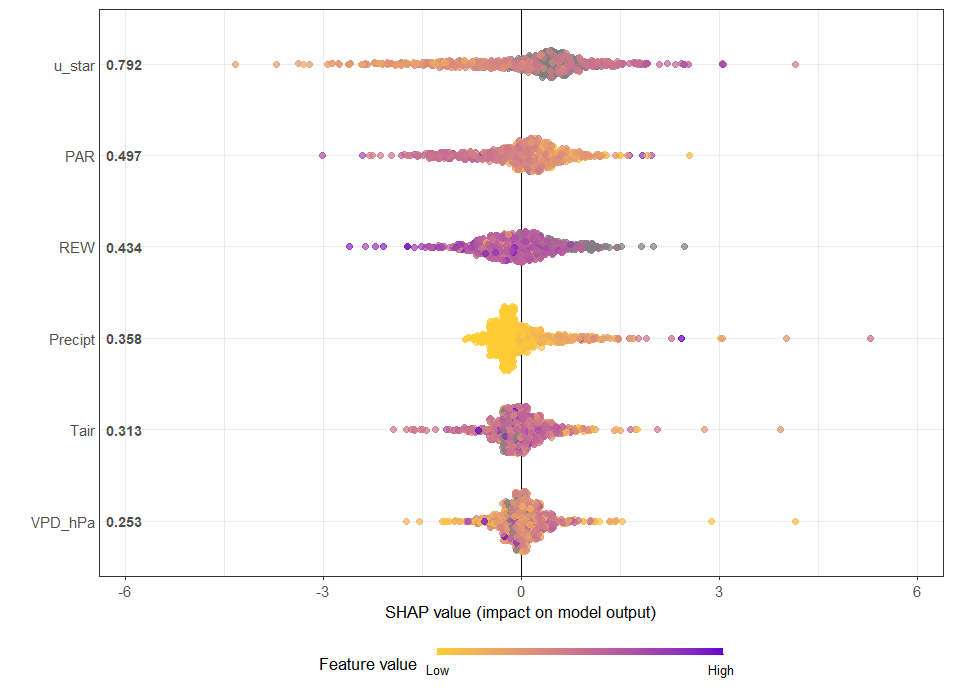 | 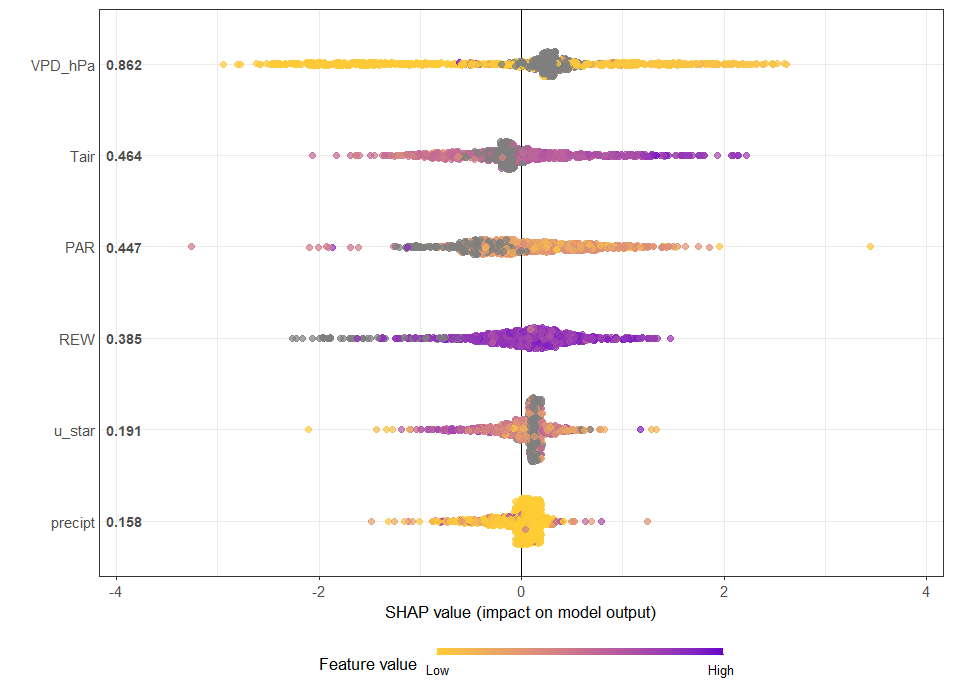 |
| PDF*: Medium (NEE) | |
| 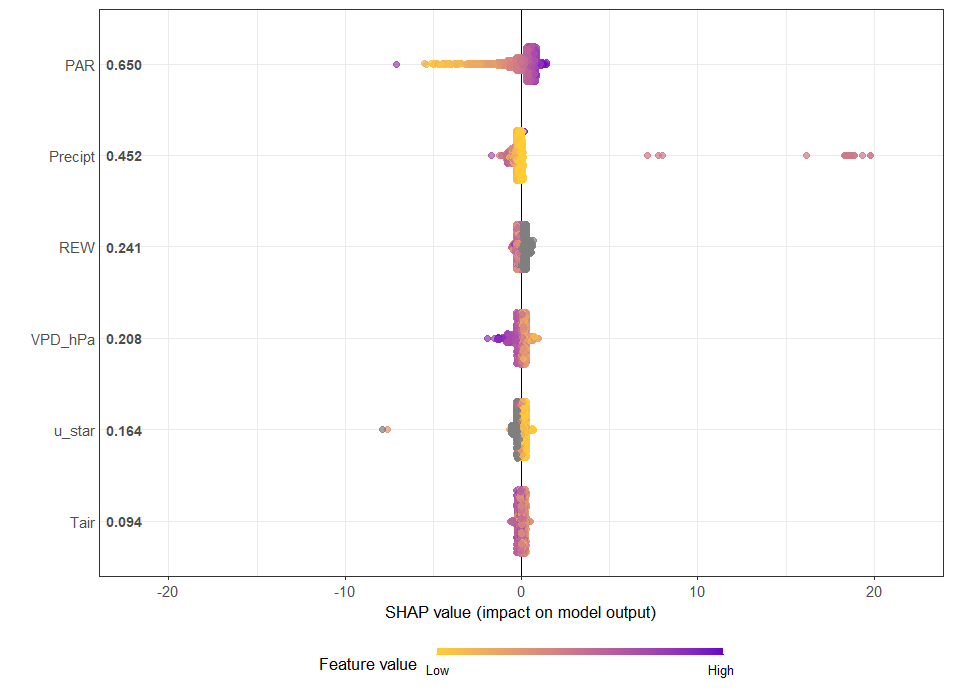 | |

Compiled Fig S2 (cont). Contribution of meteorological drivers, energy, and water fluxes to **NEE** under different LUI.

| JOP: High (NEE) | SBW*: High (NEE) |
| --- | --- |
| 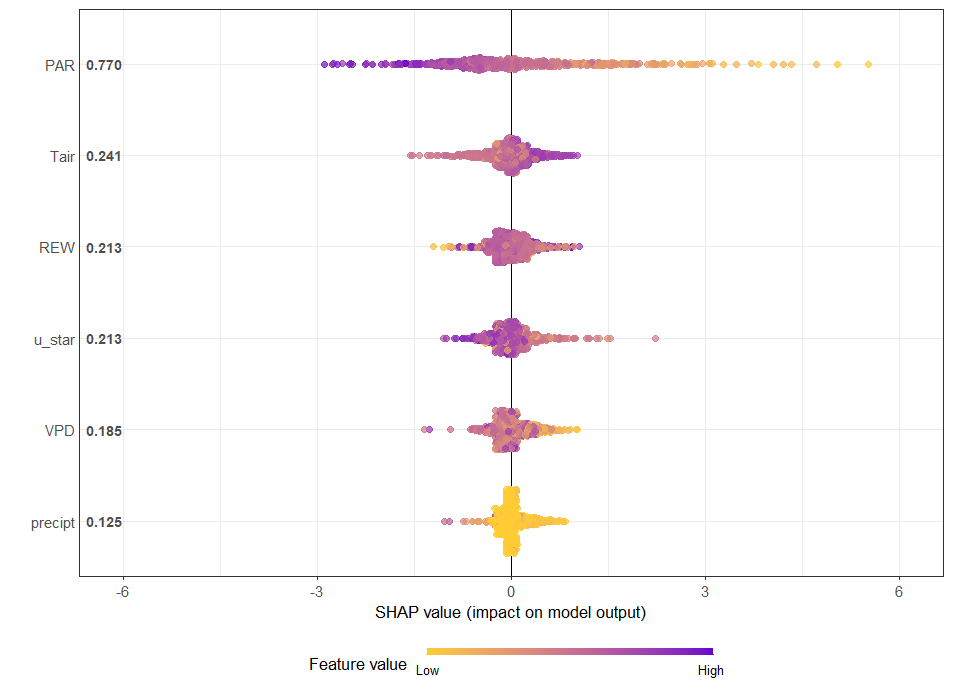 | 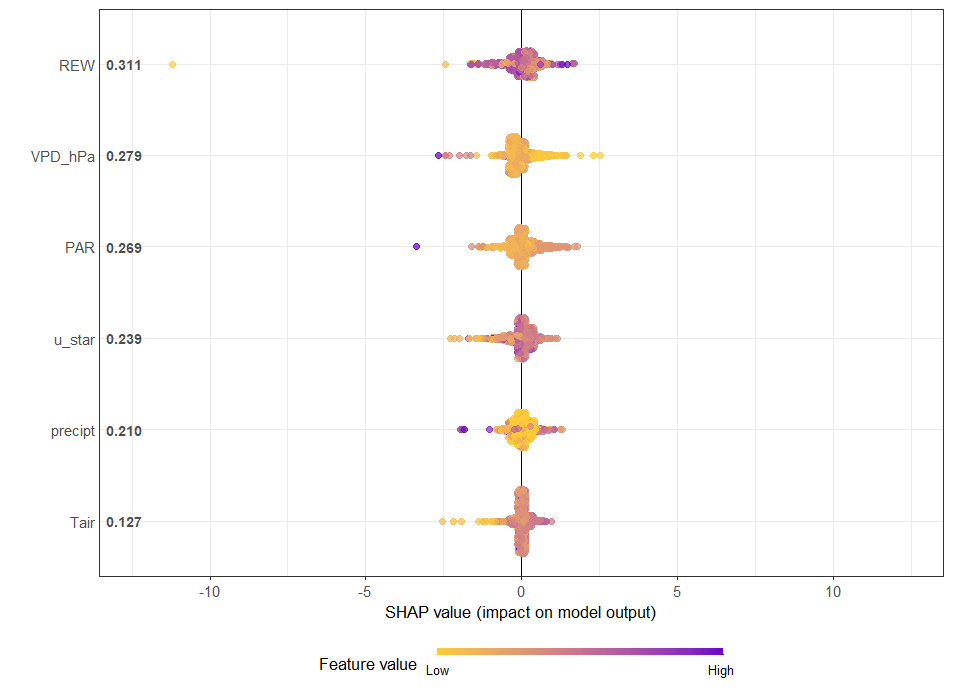 |
| RFN: High (NEE) | RFC: High (NEE) |
| 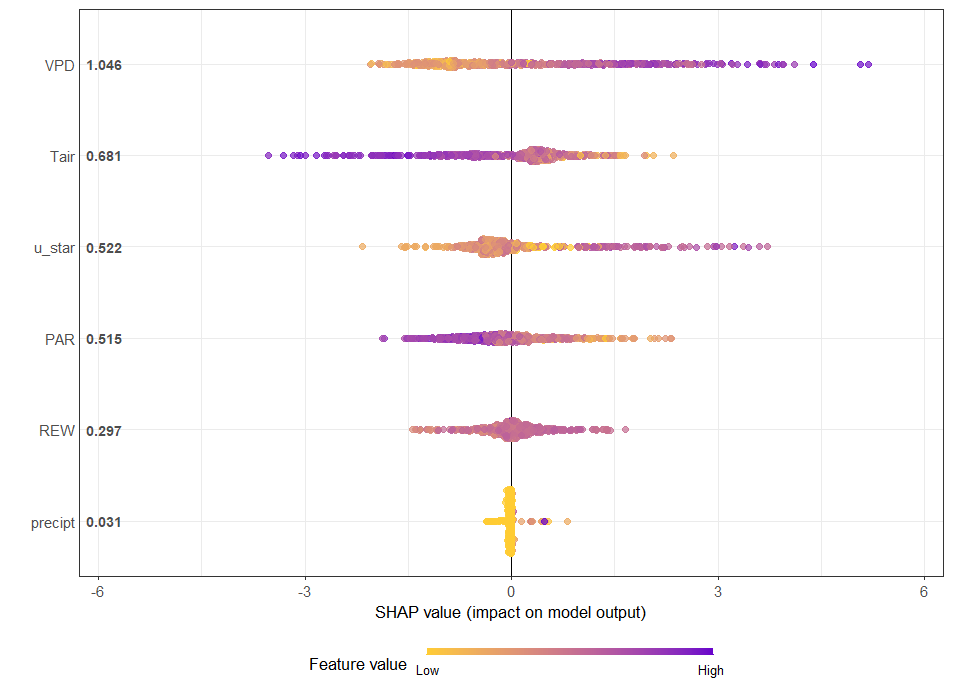 | 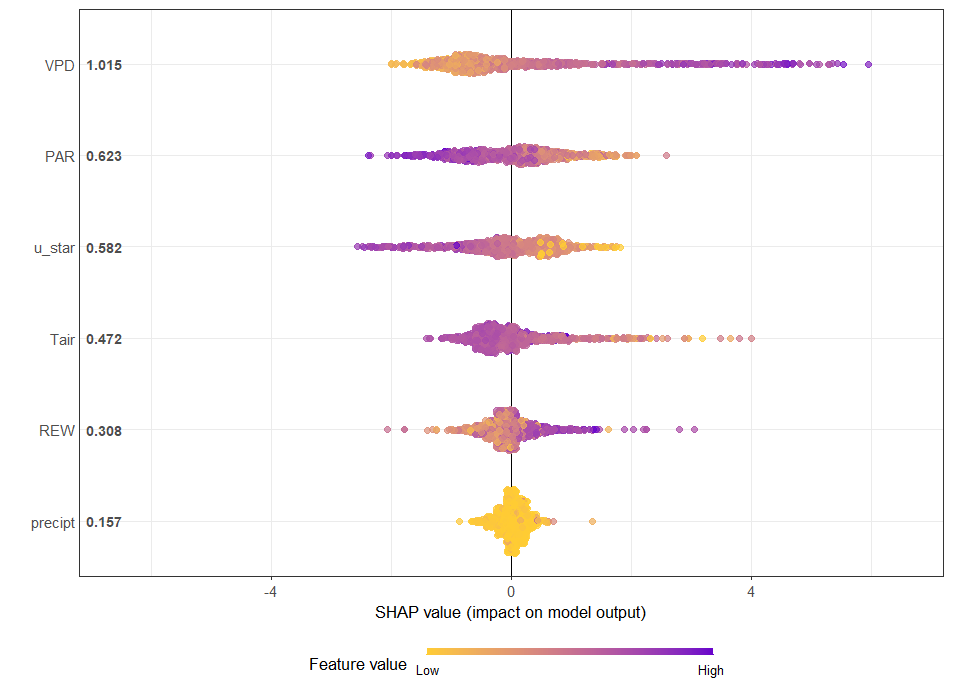 |

Compiled Fig S3. Contribution of meteorological drivers, energy, and water fluxes to **ET** under different LUI.

| LHP: Low (ET) | MKL: Low (ET) |
| --- | --- |
| 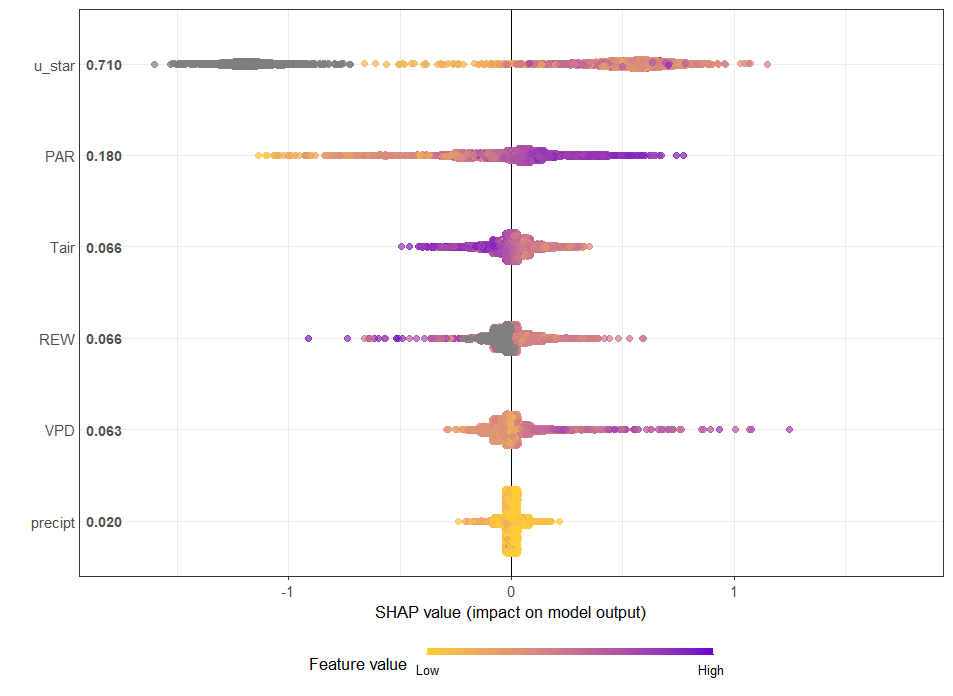 | 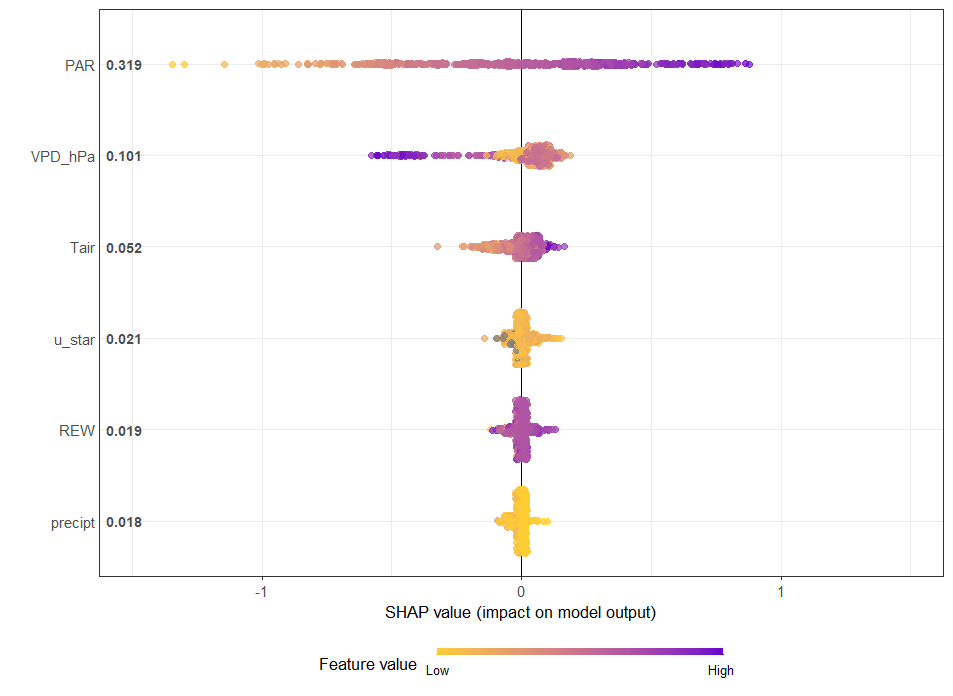 |
| SKR: Low (ET) | PSO: Low (ET) |
| 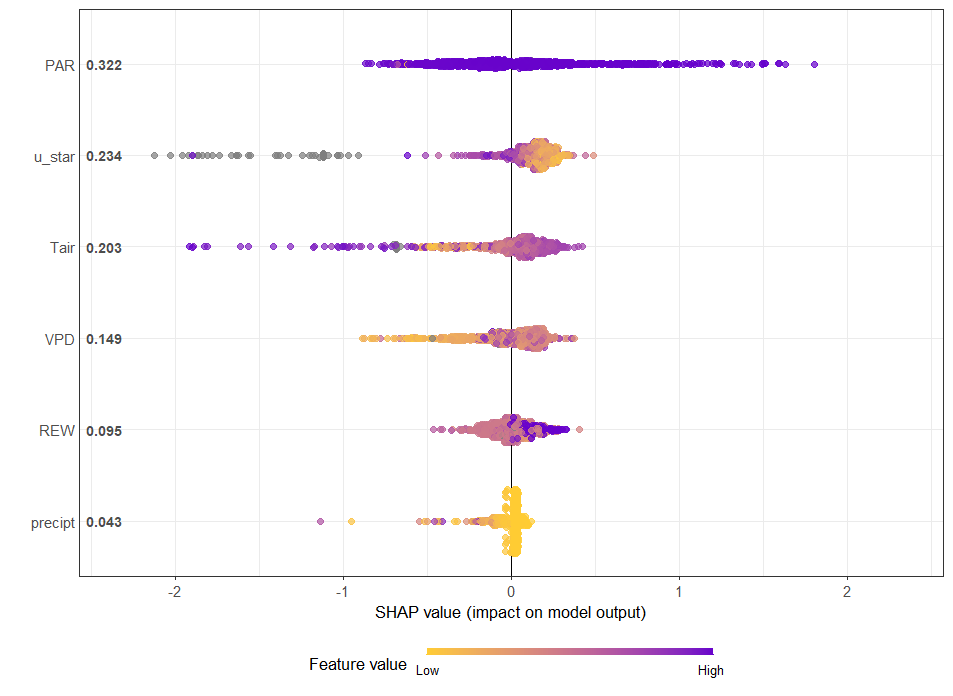 | 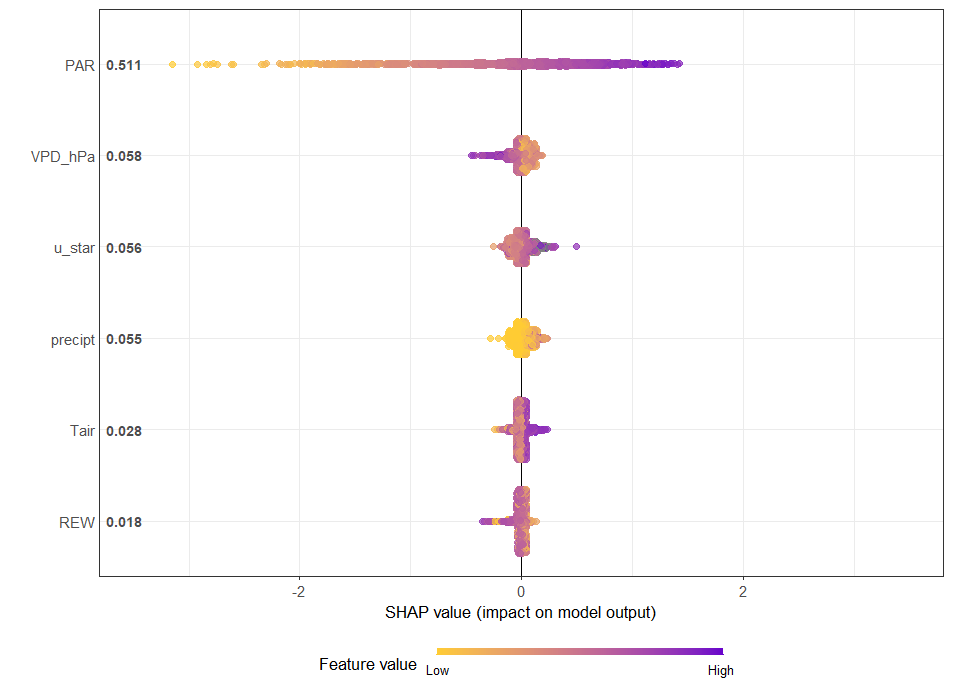 |

Compiled Fig S3 (cont). Contribution of meteorological drivers, energy, and water fluxes to **ET** under different LUI.

| BKS: Medium (ET) | DFR: Medium (ET) |
| --- | --- |
| 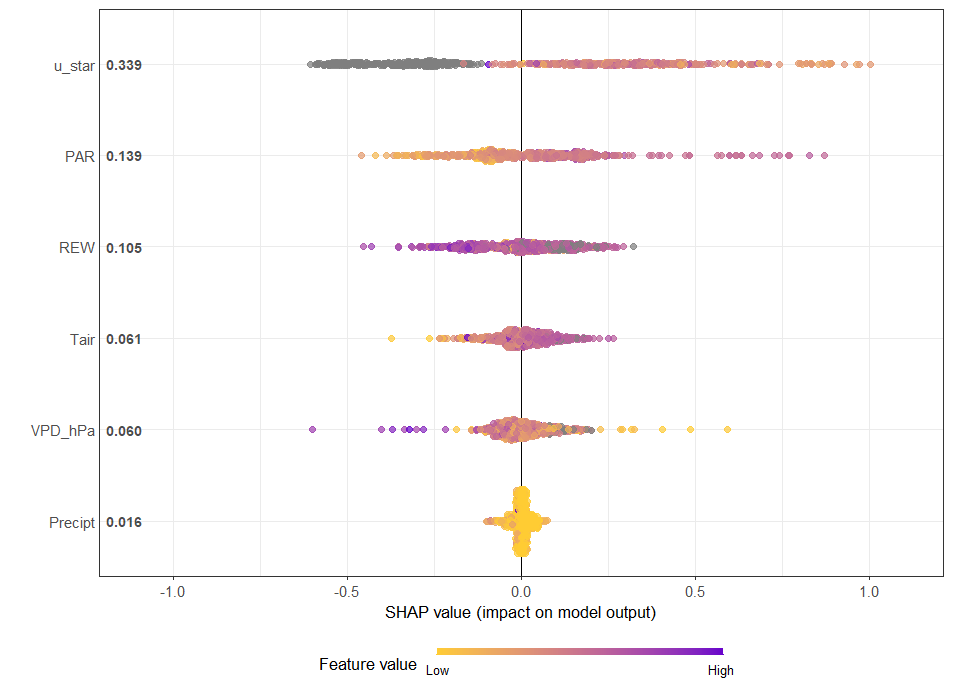 | 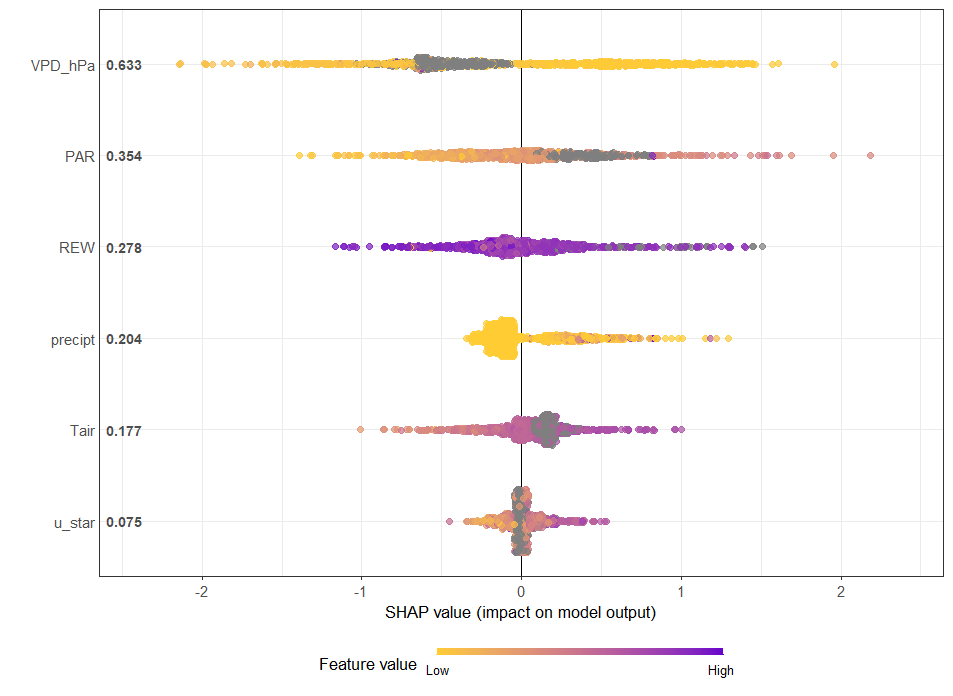 |
| PDF*: Medium (ET) | |
| 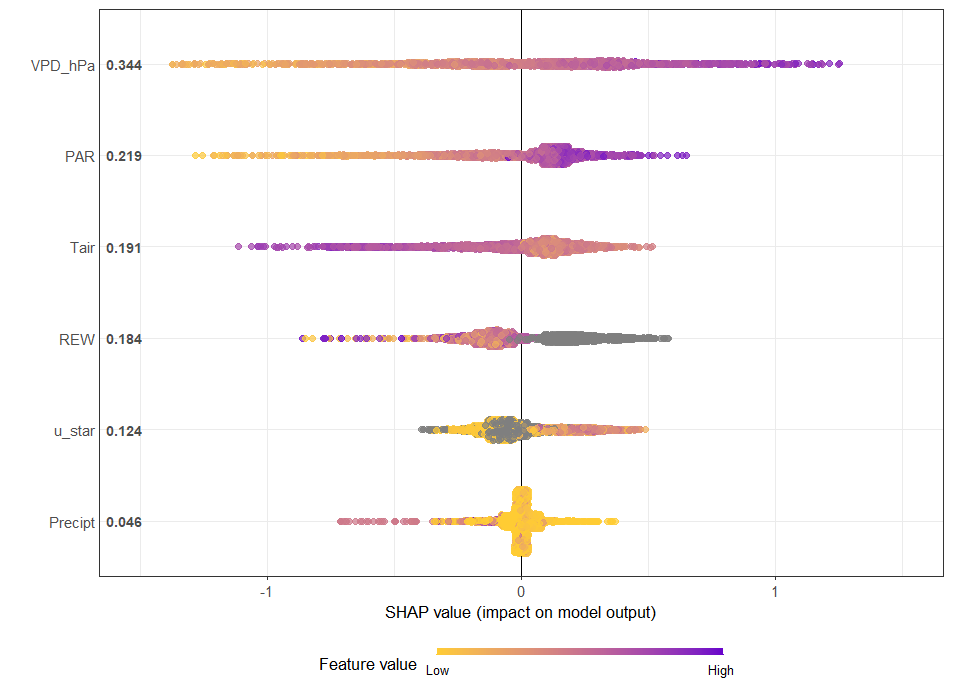 | |

Compiled Fig S3 (cont). Contribution of meteorological drivers, energy, and water fluxes to **ET** under different LUI.

| JOP: High (ET) | |
| --- | --- |
| 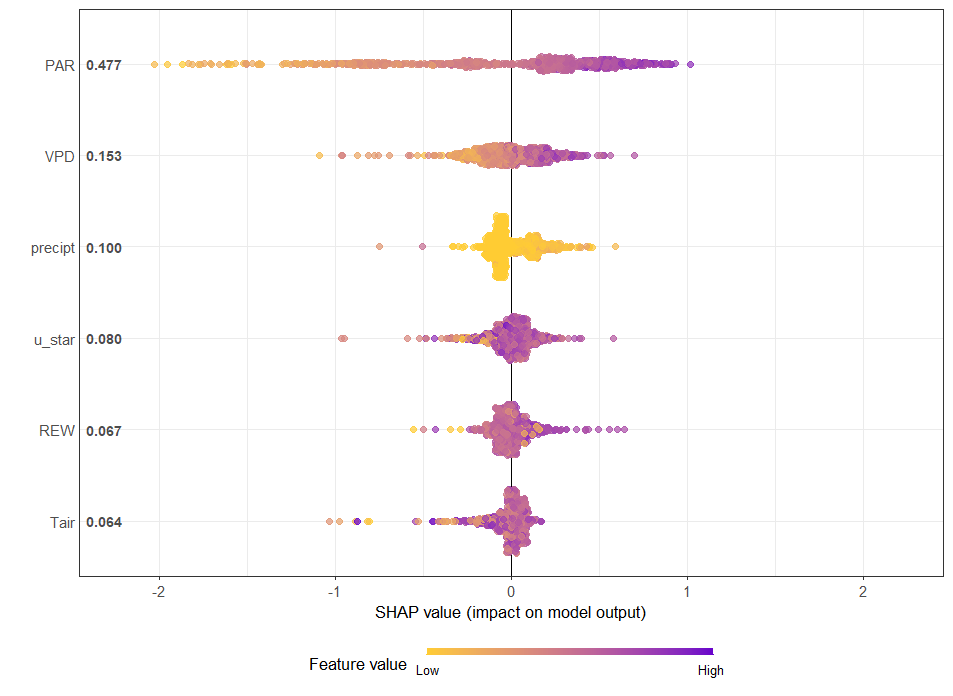 | |
| RFN: High (ET) | RFC: High (ET) |
| 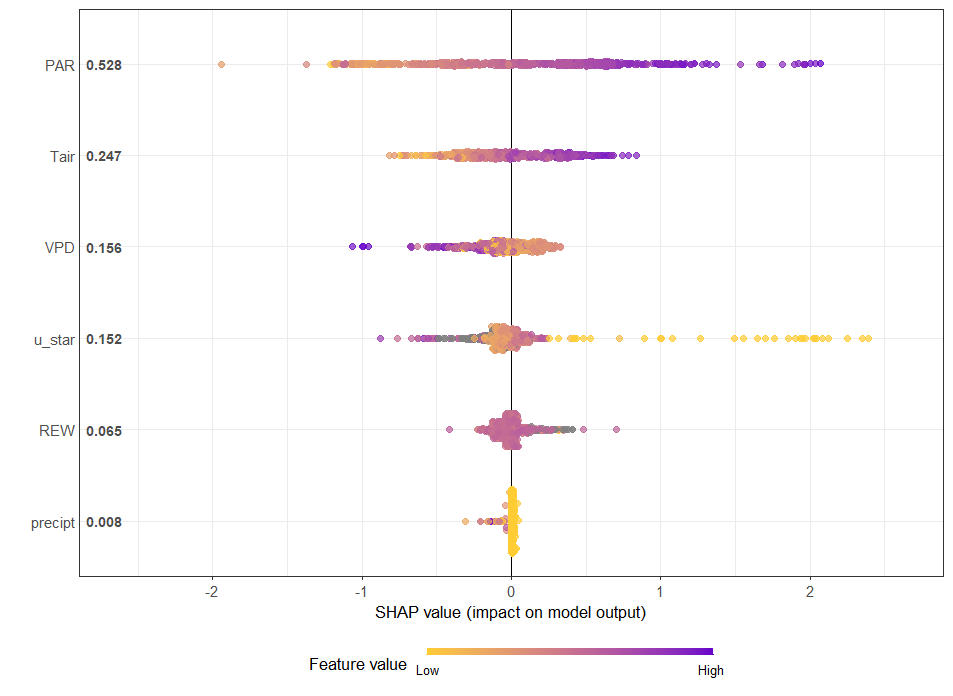 | 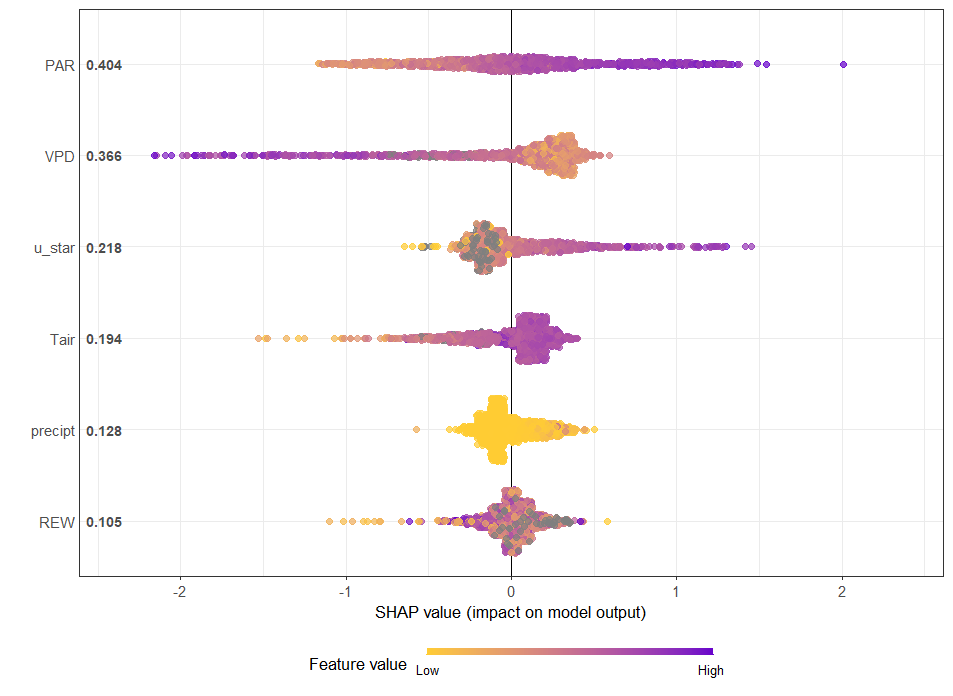 |

| a) | b) |
| --- | --- |
| 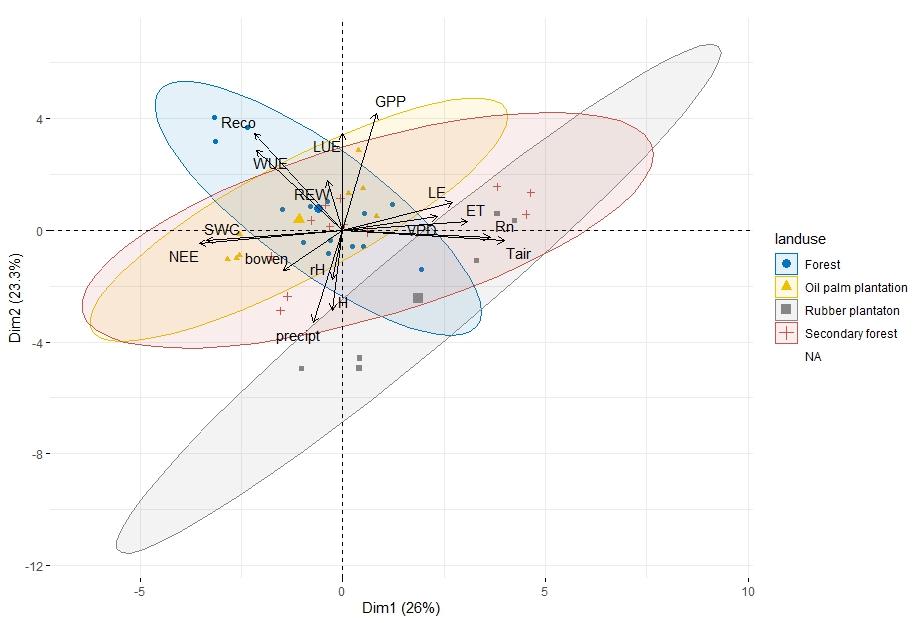 | 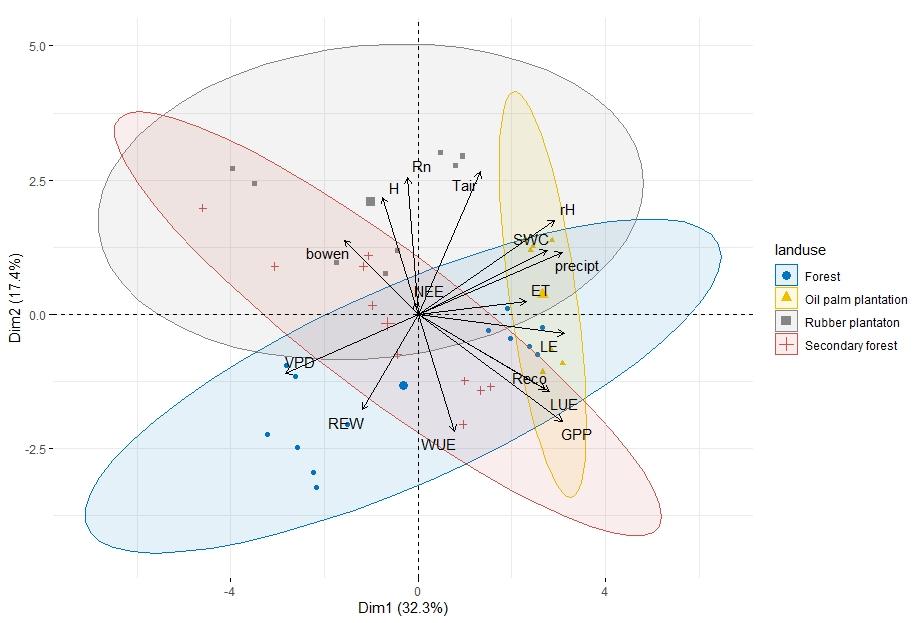 |
| c) | |
| 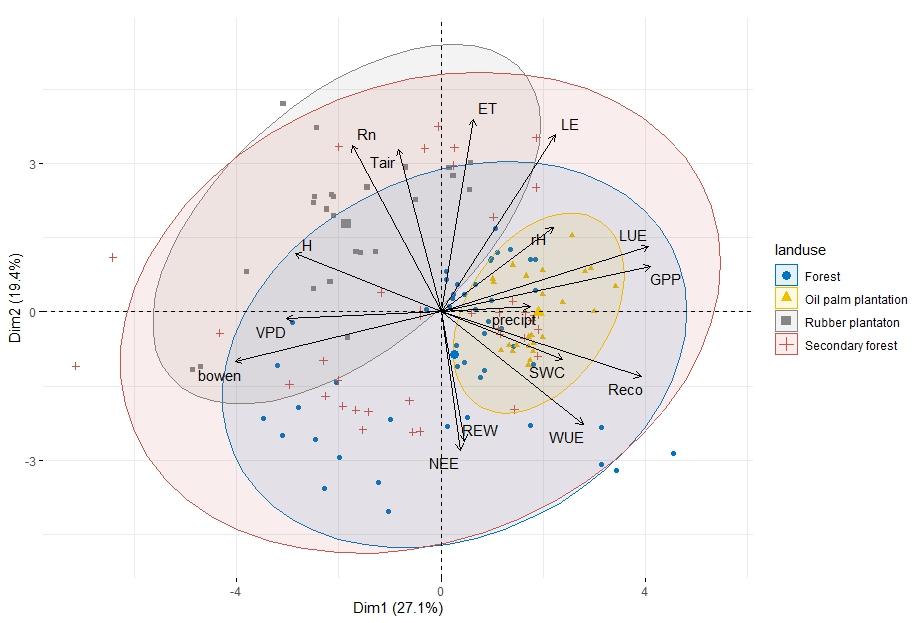 | |

Fig S4. Principal component analysis (PCA) bi-plot of carbon (NEE, GPP, Reco), energy (Rn, LE, H, LUE, water (WUE, REW, SWC), and meteorology (Tair, precipitation, ET, VPD) parameters for each land use intensity gradient in a) dry seasons, b) wet seasons, and c) all seasons.

# References

Aguilos, M. et al., 2018. Interannual and Seasonal Variations in Ecosystem Transpiration and Water Use Efficiency in a Tropical Rainforest. Forests, 10(1).

Aguirre, A.M.L. and Trilleras, A.B., 2024. Variability of Water Use Efficiency of Gmelina arborea Plantations in the Tropical Dry Forest of Colombia. Forests, 15(7).

Alves, M.P. et al., 2024. Carbon and Energy Balance in a Primary Amazonian Forest and Its Relationship with Remote Sensing Estimates. Remote Sensing, 16(19).

Besnard, S. et al., 2018. Quantifying the effect of forest age in annual net forest carbon balance. Environmental Research Letters, 13(12).

Chinchilla-Soto, C., Durán-Quesada, A.M., Monge-Muñoz, M. and Gutiérrez-Soto, M.V., 2021. Quantifying the Annual Cycle of Water Use Efficiency, Energy and CO2 Fluxes Using Micrometeorological and Physiological Techniques for a Coffee Field in Costa Rica. Forests, 12(7).

Costa, G.B. et al., 2022. WUE and CO2 Estimations by Eddy Covariance and Remote Sensing in Different Tropical Biomes. Remote Sensing, 14(14).

Deshmukh, C.S. et al., 2023. Net greenhouse gas balance of fibre wood plantation on peat in Indonesia. Nature, 616(7958): 740-746.

Fu, Z. et al., 2018. The surface-atmosphere exchange of carbon dioxide in tropical rainforests: Sensitivity to environmental drivers and flux measurement methodology. Agricultural and Forest Meteorology, 263: 292-307.

Hirano, T. et al., 2024. Large variation in carbon dioxide emissions from tropical peat swamp forests due to disturbances. Communications Earth & Environment, 5(1).

Kato, T. and Tang, Y., 2008. Spatial variability and major controlling factors of CO2 sink strength in Asian terrestrial ecosystems: evidence from eddy covariance data. Global Change Biology, 14(10): 2333-2348.

Kosugi, Y. et al., 2008. CO2 exchange of atropical rainforest at Pasoh in Peninsular Malaysia. Agricultural and Forest Meteorology, 148: 439-452.

Kosugi, Y. et al., 2012. Effect of inter-annual climate variability on evapotranspiration and canopy CO2 exchange of a tropical rainforest in Peninsular Malaysia. Journal of Forest Research, 17(3): 227-240.

Kunert, N., El-Madany, T.S., Aparecido, L.M.T., Wolf, S. and Potvin, C., 2019. Understanding the controls over forest carbon use efficiency on small spatial scales: Effects of forest disturbance and tree diversity. Agricultural and Forest Meteorology, 269-270: 136-144.

Lin, Y. et al., 2018. Water-use efficiency and its relationship with environmental and biological factors in a rubber plantation. Journal of Hydrology, 563: 273-282.

Maier, C.A. et al., 2017. Comparative water use in short-rotation Eucalyptus benthamii and Pinus taeda trees in the Southern United States. Forest Ecology and Management, 397: 126-138.

McCalmont, J. et al., 2023. Oil palm (Elaeis guineensis) plantation on tropical peatland in South East Asia: Photosynthetic response to soil drainage level for mitigation of soil carbon emissions. Sci Total Environ, 858(Pt 1): 159356.

Meijide, A. et al., 2020. Measured greenhouse gas budgets challenge emission savings from palm-oil biodiesel. Nat Commun, 11(1): 1089.

Meijide, A. et al., 2017. Controls of water and energy fluxes in oil palm plantations: Environmental variables and oil palm age. Agricultural and Forest Meteorology, 239: 71-85.

Mendes, K.R. et al., 2020. Seasonal variation in net ecosystem CO(2) exchange of a Brazilian seasonally dry tropical forest. Sci Rep, 10(1): 9454.

Papale, D. and Valentini, R., 2003. A new assessment of European forests carbon exchanges by eddy fluxes and artificial neural network spatialization. Global Change Biology, 9(4): 525-535.

Pastorello, G. et al., 2020. The FLUXNET2015 dataset and the ONEFlux processing pipeline for eddy covariance data. Sci Data, 7(1): 225.

Reichstein, M. et al., 2005. On the separation of net ecosystem exchange into assimilation and ecosystem respiration: review and improved algorithm. Global Change Biology, 11(9): 1424-1439.

Saigusa, N. et al., 2013. Dataset of CarboEastAsia and uncertainties in the CO2 budget evaluation caused by different data processing. Journal of Forest Research, 18(1): 41-48.

Takamura, N. et al., 2023. El Niño-Southern Oscillation forcing on carbon and water cycling in a Bornean tropical rainforest. Proceedings of the National Academy of Sciences, 120(42).

Ueyama, M. et al., 2012. Influences of various calculation options on heat, water and carbon fluxes determined by open- and closed-path eddy covariance methods. Tellus B: Chemical and Physical Meteorology, 64(1).

Wang, X. et al., 2022. Carbon and Water Cycling in Two Rubber Plantations and a Natural Forest in Mainland Southeast Asia. Journal of Geophysical Research: Biogeosciences, 127(5).

Wutzler, T. et al., 2018. Basic and extensible post-processing of eddy covariance flux data with REddyProc. Biogeosciences, 15(16): 5015-5030.

Xu, T. et al., 2018. Evaluating Different Machine Learning Methods for Upscaling Evapotranspiration from Flux Towers to the Regional Scale. Journal of Geophysical Research: Atmospheres, 123(16): 8674-8690.
